# Factorial Mendelian Randomization of Lp(a) Lowering, LDL-C Lowering, and Lifestyle Improvements: Joint Associations with Cardiovascular Risk

**DOI:** 10.1093/ije/dyaf020

**Published:** 2025-03-10

**Authors:** Lijuan Wang, Fangyuan Jiang, Jing Sun, Jianhui Zhao, Yazhou He, Dipender Gill, Stephen Burgess, Susanna C. Larsson, Shuai Yuan, Xue Li

**Affiliations:** 1 The Second Affiliated Hospital and School of Public Health, Zhejiang University School of Medicine, Hangzhou, China; 2 Department of oncology, West China School of Public Health and West China Fourth Hospital, Sichuan University, Chengdu, China; 3 Department of Epidemiology and Biostatistics, School of Public Health, Imperial College London, London, UK; 4 Sequoia Genetics, London, UK; 5 MRC Biostatistics Unit, University of Cambridge, Cambridge, UK; 6 Department of Public Health and Primary Care, University of Cambridge, Cambridge, UK; 7 Unit of Cardiovascular and Nutritional Epidemiology, Institute of Environmental Medicine, Karolinska Institute, Stockholm, Sweden; 8 Unit of Medical Epidemiology, Department of Surgical Sciences, Uppsala University, Uppsala, Sweden; 9 Department of Surgery, University of Pennsylvania Perelman School of Medicine, Philadelphia, PA, USA; 10 Corporal Michael J. Crescenz VA Medical Center, Philadelphia, PA, USA

**Keywords:** cardiovascular disease, cardiovascular mortality, lipoprotein(a), lifestyle factor, factorial Mendelian randomization

## Abstract

**Background:**

High levels of lipoprotein(a) [Lp(a)] have been associated with an increased risk of cardiovascular disease (CVD); however, the effects of Lp(a) lowering therapy in combination with low-density lipoprotein cholesterol (LDL-C) lowering treatment or lifestyle improvements on CVD risk remain unexplored.

**Methods:**

We conducted a factorial Mendelian randomization study among 385,917 participants in the UK Biobank. Separate genetic scores were constructed to proxy the effects of Lp(a) lowering, LDL-C lowering through different targets (HMGCR, NPC1L1, PCSK9, and LDLR), as well as improvements in body mass index (BMI), systolic blood pressure (SBP) and lifestyle factors (cigarette smoking, alcohol consumption, and physical activity).

**Results:**

Genetically-predicted lower Lp(a) levels were associated with a decreased risk of CVD and CVD-specific mortality. Per 50-mg/dL, the hazard ratio (HR) ranged from 0.73 [95% confidence interval (CI): 0.73, 0.73] for peripheral artery disease (PAD) to 0.95 (95% CI: 0.92, 0.99) for venous thromboembolism (VTE). In factorial analyses exploring combined exposure to low-level Lp(a) and low-level LDL-C, there was no consistent evidence for departure from an additive model for any outcome (*P*_interaction_ > 0.05), with the exception of the analysis using the *LDLR* score and PAD (*P*_interaction_ = 0.006). In factorial analyses exploring combination therapies integrating Lp(a) lowering with interventions on BMI, SBP and lifestyle factors, there was no evidence for departure from an additive model in any analysis (*P*_interaction_ > 0.05).

**Conclusions:**

Our study suggests that Lp(a) lowering will have a similar magnitude for reducing cardiovascular events whether it is considered alone, or in conjunction with LDL-C reduction or lifestyle improvements.

## Introduction

Cardiovascular disease (CVD), which affects more than 500 million people worldwide, poses a heavy burden on the health system.^1^ High levels of low-density lipoprotein cholesterol (LDL-C) and unhealthy lifestyle factors have been listed as top-ranked contributors to this global cardiovascular pandemic and related high mortality and disease burden.^1, 2^ High lipoprotein(a) [Lp(a)] levels have been also found to be associated with an increased risk of CVD and proposed as another emerging risk factor for CVD onset.^3^

Both Lp(a) and LDL-C are lipoproteins involved in cholesterol transport in the blood and have been linked to CVD, thus they may interact with each other and treatments targeting the two markers may exhibit synergistic effects. For example, earlier prospective studies identified that the associations of Lp(a) with the risk of CVDs may be more marked among individuals with elevated LDL cholesterol,^4, 5^ and treating elevated LDL-C in those with elevated Lp(a) has been shown to attenuate the increased risk of coronary artery disease (CAD).^6^ In addition, body weight, blood pressure and certain lifestyle factors may interact with Lp(a) through their effects on lipid metabolism and cardiovascular health. Briefly, body weight plays a crucial role, as obesity is always linked to higher LDL-C and potentially elevated Lp(a).^7^ Although elevated blood pressure itself may not increase Lp(a), both conditions can coexist and interact to amplify cardiovascular risk.^8^ Smoking and alcohol consumption can impact lipid profiles by affecting liver function and increasing oxidative stress, respectively.^9^ While regular physical activity enhances lipid clearance from the bloodstream, thereby potentially lowering both LDL-C and Lp(a).^10^

Lipid lowering therapies and lifestyle modifications have been recommended to lower the risk of CVD.^11, 12^ Lp(a) is a novel causal risk factor for CVD^13^ and has been identified as a potential target for lowering CVD risk, although no directed therapies have been approved in the United States or Europe.^14^ Pelacarsen, an antisense oligonucleotide targeting apolipoprotein(a), shows promise as a therapy for Lp(a) lowering.^15^ However, its effects on cardiovascular outcomes are unknown and are being investigated in the Lp(a) HORIZON trial (phase 3).^16^ Besides, the interactions across Lp(a) lowering, LDL-C lowering, and lifestyle improvements in modifying CVD risk remain unexplored, yet understanding this interplay is crucial for improving our understanding of the etiological basis of CVD and optimizing prevention and treatment strategies, particularly for high-risk patients.

The Mendelian randomization (MR) paradigm is an epidemiological approach based on observational genetic data with the merit of reinforcing causal inference by minimizing risk of confounding and reverse causation.^17^ Several previous MR studies have established causal associations of Lp(a) lowering and LDL-C lowering therapies with CVD risk by using genetic variants as instruments to mimic the therapeutic effects.^13, 18–20^ The factorial MR analysis, designed to mimic a randomized controlled trial (RCT), has been developed to examine the interactions of two interventions.^21, 22^ Here, we conducted a factorial MR study to assess the effects of genetically predicted low-level Lp(a) on a broad range of CVD outcomes as well as to explore its interactions with genetically predicted low-level LDL-C and interventions on BMI, SBP and lifestyles.

## Methods

### Study design

**Figure 1:**
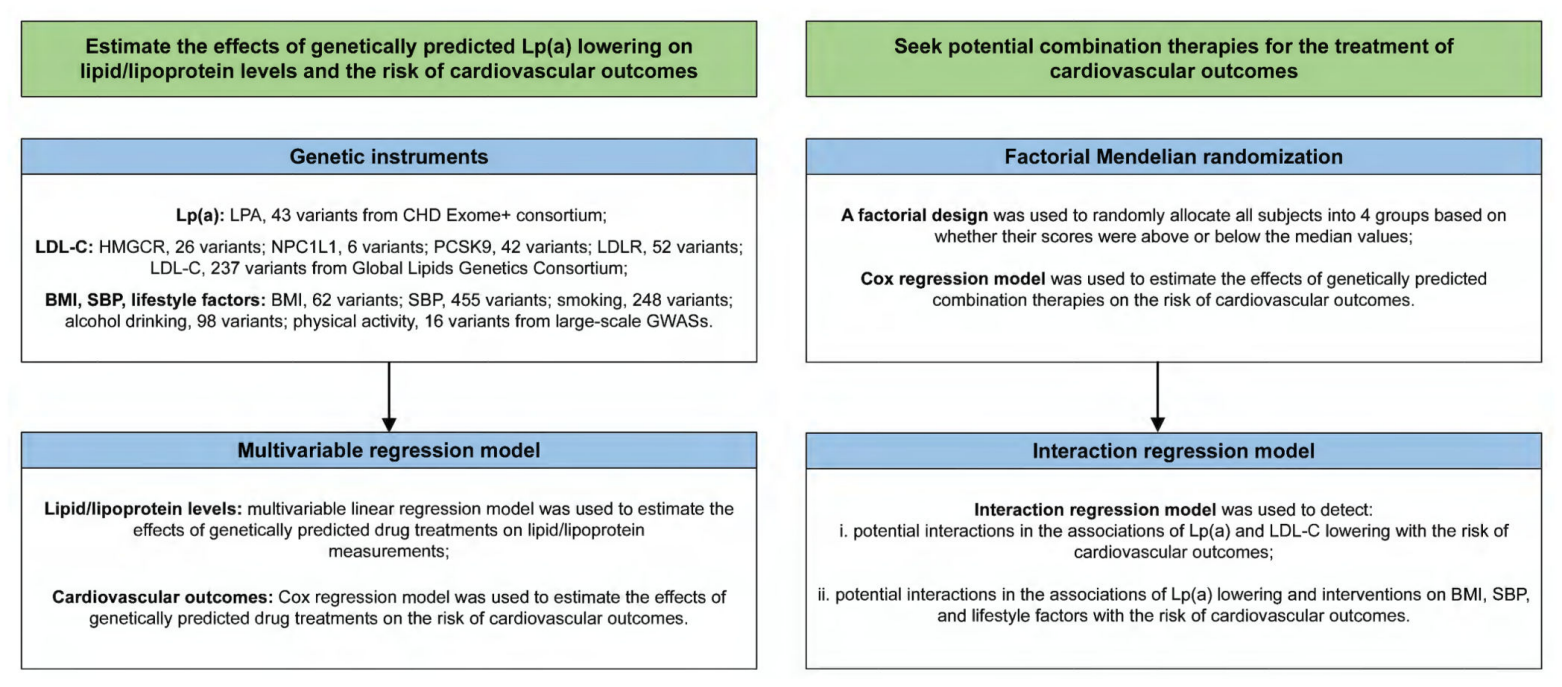
Study design overview. BMI, body mass index; CHD, coronary heart disease; GWASs, genome-wide association studies; HMGCR, 3-hydroxy-3-methylglutaryl-CoA reductase; LDL-C, low-density lipoprotein cholesterol; LDLR, low density lipoprotein receptor; LPA, lipoprotein(a); Lp(a), lipoprotein(a); NPC1L1, NPC1 like intracellular cholesterol transporter 1; PCSK9, proprotein convertase subtilisin/kexin type 9; SBP, systolic blood pressure. **Alt text:** Two columns of boxes showing how the researchers aimed to estimate the effects of genetically predicted lipoprotein(a) lowering using genetic instuments and multivariable regression, and to seek combination therapies to treat cardiovascular outcomes using factorial Mendelian randomisation and interaction regression.

We first explored the associations of genetically proxied Lp(a) lowering and LDL-C lowering with the levels of blood lipids and lipoproteins to examine the validity of selected instruments. Then, we estimated the associations of genetically predicted low-level Lp(a) with major CVDs, cardiovascular mortality, and all-cause mortality. Finally, we conducted factorial MR to investigate the joint effects of genetically predicted Lp(a) lowering and LDL-C lowering or lifestyle improvements in the risk of CVDs and mortality (Figure 1).

### Study population

The UK Biobank study is a population-based prospective cohort study, which recruited over 500,000 adults aged between 40 and 69 years in 2006-2010^23^ To minimize the influence of diverse population structure, we excluded participants with high heterozygosity, or with high missing rate, sex mismatch, or putative aneuploidy in sex chromosome, or non-White ancestry, or with high relatedness. After filtering ineligible samples, the current study was based on 385,917 UK Biobank participants.

### Outcome ascertainment

Major cardiovascular outcomes included coronary artery disease (CAD), peripheral arterial disease (PAD), stroke and its subtypes [i.e., ischemic stroke (IS), intracerebral hemorrhage (ICH), and subarachnoid hemorrhage (SAH)], atrial fibrillation (AF), heart failure (HF), and venous thromboembolism (VTE). Two additional composites [i.e., 3- and 4-point major adverse cardiovascular events (MACE)^24, 25^], and cardiovascular and all-cause mortality were also considered. Detailed definitions of these studied outcomes are shown in Supplementary Table S1. We included both prevalent and incident cases, with participants followed up until December 31, 2021.

### Genetic instruments

*LPA* gene region was ultra-finely mapped among 48,333 individuals from the CHD Exome+ consortium using a customized version of the Illumina Exome Beadchip array.^26^ We selected the variants associated with Lp(a) levels at the genome-wide levels of significant (*P* < 5×10^-8^) and generated a list of 43 single nucleotide polymorphisms (SNPs) which was demonstrated to be strong and validated genetic instruments (IVs) for Lp(a)^26^. Then, a weighted genetic score that reflected genetically predicted levels of Lp(a) was calculated using individual-level data in the UK Biobank weighted by each variant’s association with the change in Lp(a) levels in milligrams per deciliter.

We used summary data of European populations from a genome-wide meta-analysis of LDL-C levels in the Global Lipids Genetics Consortium^27^ to generate genetic instruments to proxy LDL-C lowering via HMG-CoA reductase (HMGCR), NPC1 like intracellular cholesterol transporter 1 (NPC1L1), proprotein convertase subtilisin/kexin type 9 (PCSK9) and low density lipoprotein receptor (LDLR). For each target, we constructed a genetic score by including all variants within 100 kb on either side of each gene that were associated with LDL-C levels at *P* < 5.0×10^−8^ and that were in weak linkage disequilibrium (*r^2^* < 0.2) to increase the proportion of variance explained by the instruments. In total, 26 SNPs for HMGCR, 6 SNPs for NPC1L1, 42 SNPs for PSCK9, and 52 SNPs for LDLR were obtained, respectively.^19, 21, 28^ We also established a genetic score to proxy overall LDL-C levels (not specific to any lipid lowering drug target) that included 237 independent SNPs (*r^2^* < 0.001) associated with LDL-C levels at genome-wide significance level (*P* < 5.0×10^−8^), serving as a positive control.^29^ Detailed information on the genetic instruments is presented in the Supplementary Table S2-S4.

Body mass index (BMI), systolic blood pressure (SBP) and lifestyle factors (smoking, alcohol drinking and physical activity) were also included in this study. For each factor, we selected associated genetic instruments at the genome-wide significance (*P* < 5.0×10^−8^) from external large-scale genome-wide association studies (GWASs),^30–33^ and clumped them using a linkage disequilibrium threshold of r^2^ < 0.001 according to the European reference panel of the 1000 Genomes project. As a result, 62 SNPs for BMI, 455 SNPs for SBP, 248 SNPs for smoking initiation, 98 SNPs for alcohol consumption per week, and 16 SNPs for moderate-to-vigorous intensity physical activity during leisure time (MVPA) were included. Details regarding the related studies and the instruments are presented in Supplemental Table S5 and S6.

### Statistical analysis

We divided the population into higher-level and lower-level groups based on the median values of genetic scores and applied a multivariable linear regression model to estimate the associations between genetically predicted lower levels of Lp(a) and LDL-C and the measurements of 7 blood lipids and lipoproteins, with adjustment of age, sex, assessment center and the first 10 principal components (PCs). Cox proportional hazards regression analysis was conducted to estimate the multivariable-adjusted hazard ratio (HR) for CVD and mortality outcomes. The models were also adjusted for age, sex, assessment center and the first 10 PCs. In factorial MR analysis, the population was naturally randomly allocated into four subgroups based on the median values of genetic scores. We then estimated the change of blood lipids and the risk of CVD and mortality between the groups and the reference group using the multivariable linear and Cox proportional hazard regression, respectively, with adjustment for age, sex, assessment center and the first 10 PCs. Given that taking dichotomized gene scores is generally inefficient for detecting statistical interactions, we thus examined the interaction of the two arms using continuous genetic scores and their product in the interaction regression model.^34^ The tests were two-sided and performed using R software version 4.2.1.

## Results

In this study, we used the UK Biobank genetic data of 488,366 participants. Genetic quality control was done centrally by UK Biobank and a total of 385,917 unrelated European individuals were finally included, containing 177,690 (46.0%) men and 208,227 (54.0%) women. The mean age of the population was 56.7 ± 8.0 years and the mean levels of Lp(a) were 17.6 ± 19.7 milligrams per deciliter. More details regarding socio-demographic information, lifestyle factors and blood lipid concentrations, are presented in Table 1.

### Genetically predicted Lp(a) and LDL-C levels in relation to measured blood lipids

**Figure 2:**
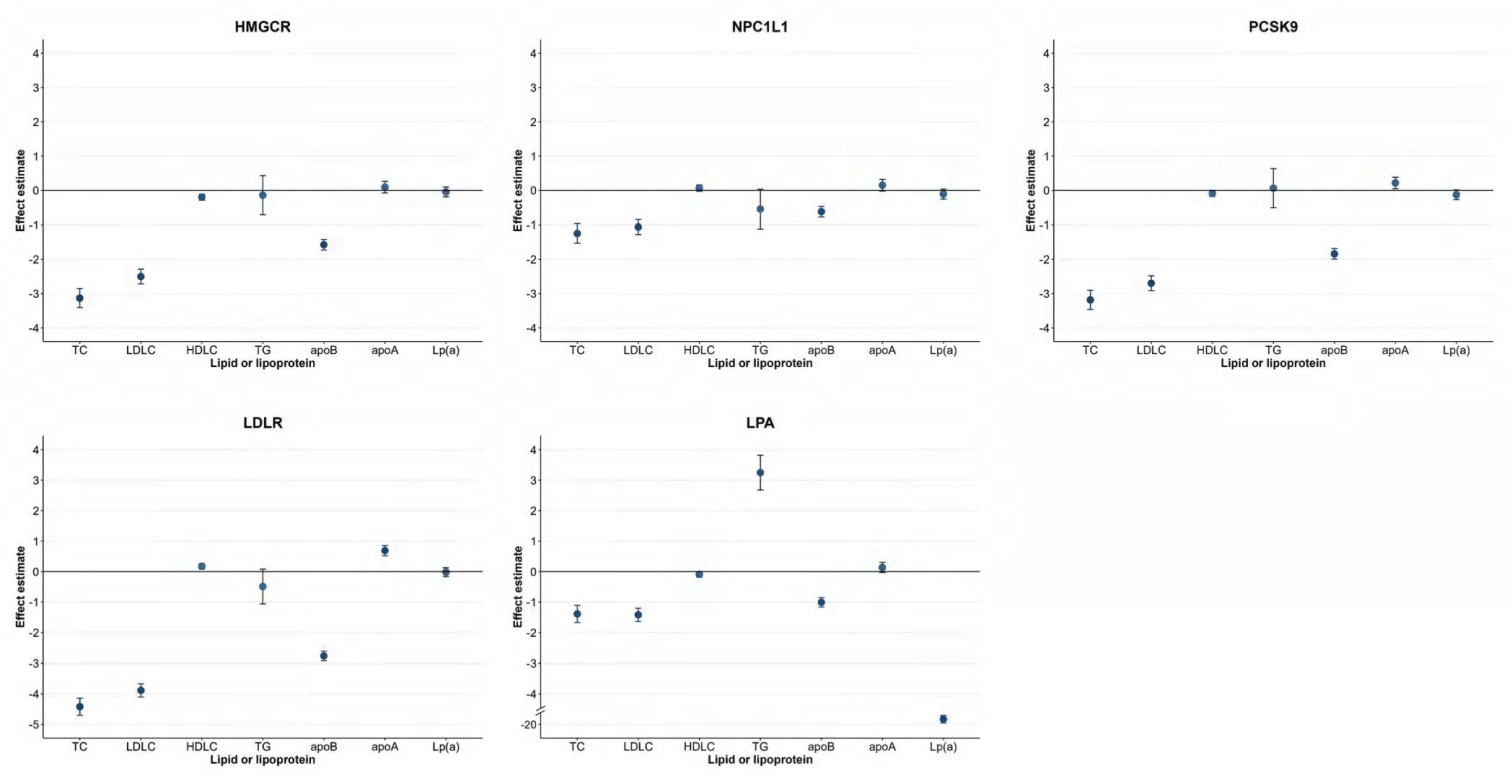
Associations of genetically predicted lipoprotein(a) [Lp(a)] and low-density lipoprotein cholesterol (LDL-C) lowering with blood lipids and lipoproteins. Multivariable linear regression was employed to estimate the effects of genetically predicted lower levels of Lp(a) and LDL-C via any targets on the measured levels of blood lipids and lipoproteins, with adjustment of age, sex, assessment center and the first 10 principal components. apoA, apolipoprotein A; apoB, apolipoprotein B; HDL-C, HDL cholesterol; HMGCR, 3-hydroxy-3-methylglutaryl-CoA reductase; LDL-C, low-density lipoprotein cholesterol; LDLR, low density lipoprotein receptor; LPA, lipoprotein(a); Lp(a), lipoprotein(a); NPC1L1, NPC1 like intracellular cholesterol transporter 1; PCSK9, proprotein convertase subtilisin/kexin type 9; TC, total cholesterol; TG, triglycerides. **Alt text:** Five graphs showing point estimates and 95% confidence interval bars of associations between genetically predicted lipoprotein(a) [Lp(a)] and low-density lipoprotein cholesterol lowering with blood lipids and lipoproteins. Genetic prediction of all studied targets was associated with decreased levels of measured LDL-C, whereas only genetic variants in the *LPA* gene region were associated with reduced levels of measured Lp(a).

Genetic prediction of all studied targets was associated with decreased levels of measured LDL-C [HMGCR: effect size, -2.50 mg/dL; 95% confidence interval (CI): -2.72, -2.29; NPC1L1: effect size, -1.06 mg/dL; 95% CI: -1.28, -0.84; PCSK9: effect size, -2.70 mg/dL; 95% CI: -2.91, -2.48; LDLR: effect size, -3.86 mg/dL; 95% CI: -4.10, -3.67; LPA: effect size, -1.42 mg/dL; 95% CI: -1.64, - 1.20], whereas only genetic variants in the *LPA* gene region were associated with reduced levels of measured Lp(a) (effect size, -19.68 mg/dL; 95% CI: - 19.80, -19.56) (Figure 2). Of note, genetic variants in the *LPA* gene region were associated with increased levels of triglycerides (effect size, 3.25 mg/dL; 95% CI: 2.68, 3.82) (Figure 2). In factorial MR, the levels of LDL-C and apoB were further reduced in individuals with genetically predicted lower levels of Lp(a) and LDL-C (Supplementary Figure S1).

### Genetically predicted Lp(a) levels and cardiovascular risk and mortality

**Figure 3:**
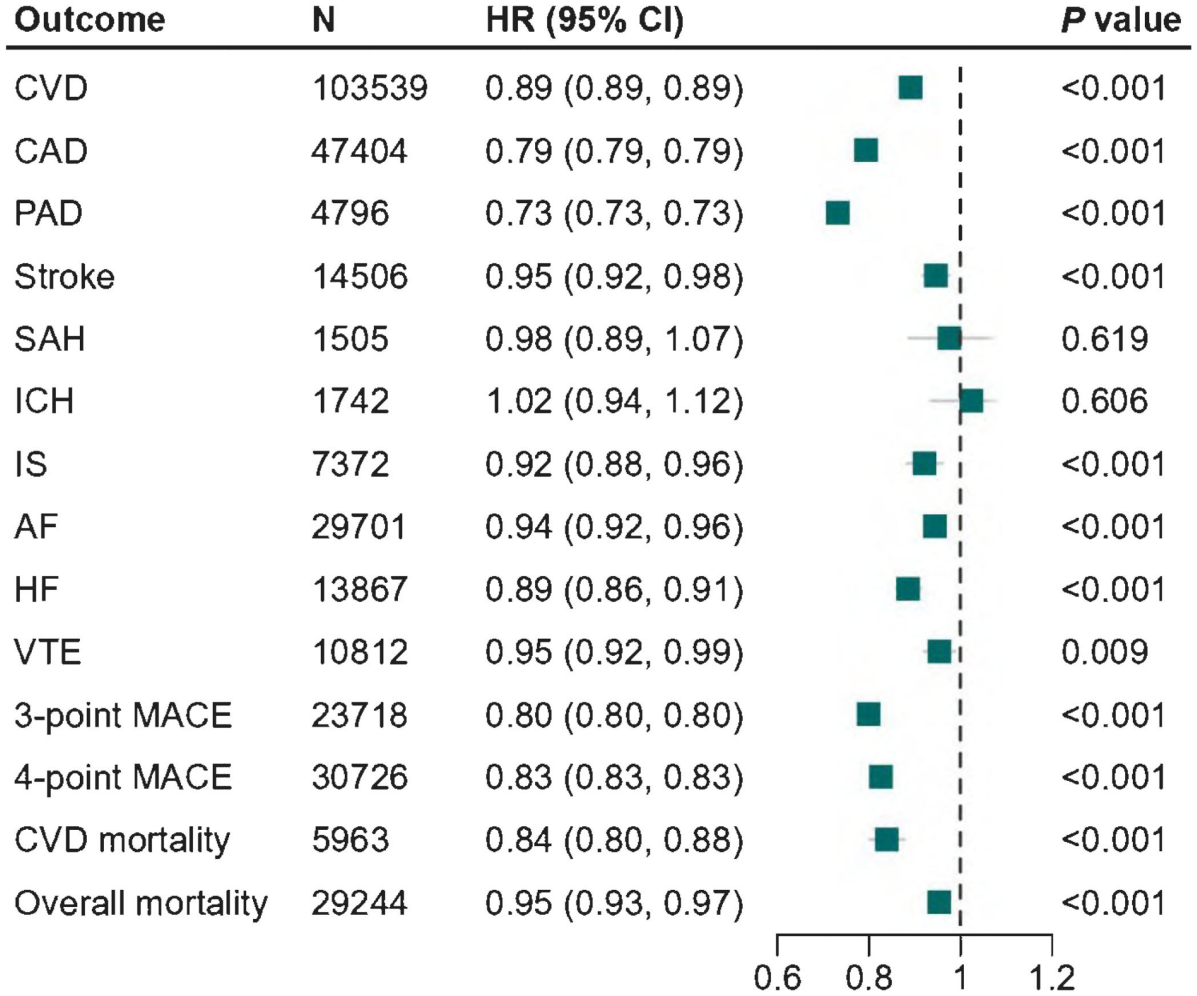
Associations of genetically predicted lipoprotein(a) [Lp(a)] lowering with the risk of cardiovascular events and mortality. Solid squares represent point estimation, and horizontal lines represent 95% confidence intervals. Cox proportional hazards regression analysis was conducted to estimate the hazard ratio (HR), with adjustment for age, sex, assessment center and the first 10 principal components. AF, atrial fibrillation; CAD, coronary artery disease; CI, confidence interval; CVD, cardiovascular disease; HF, heart failure; HR, hazard ratio; ICH, intracerebral hemorrhage; IS, ischemic stroke; MACE, major adverse cardiovascular events; PAD, peripheral arterial disease; SAH, subarachnoid hemorrhage; VTE, venous thromboembolism. **Alt text:** A forest plot showing point estimates and 95% confidence interval bars of associations between genetically predicted lipoprotein(a) [Lp(a)] and low-density lipoprotein cholesterol lowering with the risk of cardiovascular events and mortality. Genetically predicted lower levels of Lp(a) were significantly associated with a reduced risk of cardiovascular disease, coronary artery disease, peripheral artery disease, stroke, ischemic stroke, atrial fibrillation, heart failure, 3- and 4-point major adverse cardiac events, cardiovascular and all-cause mortality.

Genetically predicted lower levels of Lp(a) were significantly associated with a reduced risk of CVD, CAD, PAD, stroke, IS, AF, HF, 3- and 4-point MACE, cardiovascular and all-cause mortality. Per 50-mg/dL decrease in genetically predicted Lp(a) levels, the HR ranged from 0.73 (95% CI: 0.73, 0.73) for PAD to 0.95 (95% CI: 0.92, 0.99) for VTE (Figure 3). We did not observe an association between genetically predicted lower Lp(a) levels and the risk of SAH (HR, 0.98; 95% CI: 0.89, 1.07) and ICH (HR, 1.02; 95% CI: 0.94, 1.12) (Figure 3).

### Joint associations of genetically predicted Lp(a) and LDL-C lowering with cardiovascular risk and mortality

**Figure 4:**
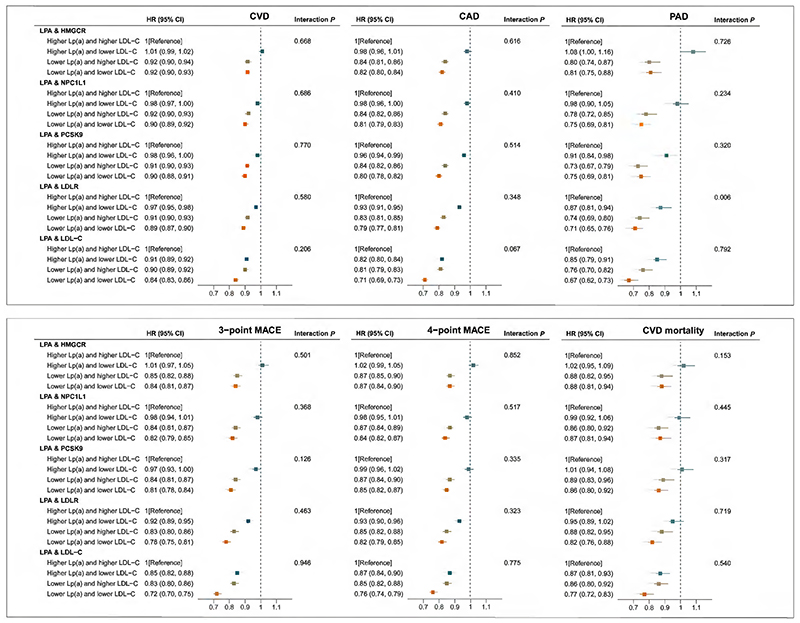
Joint associations of genetically predicted lipoprotein(a) [Lp(a)] and low-density lipoprotein cholesterol (LDL-C) lowering with the risk of major cardiovascular events. Solid squares represent point estimation, and horizontal lines represent 95% confidence intervals. For each subgroup, cox proportional hazards regression analysis was conducted to estimate the hazard ratio (HR), with adjustment for age, sex, assessment center and the first 10 principal components. The interaction *P* value was calculated by adding genetic scores as continuous variables into the model. CAD, coronary artery disease; CI, confidence interval; CVD, cardiovascular disease; HMGCR, 3-hydroxy-3-methylglutaryl-CoA reductase; HR, hazard ratio; LDL-C, low-density lipoprotein cholesterol; LDLR, low density lipoprotein receptor; LPA, lipoprotein(a); Lp(a), lipoprotein(a); MACE, major adverse cardiovascular events; NPC1L1, NPC1 like intracellular cholesterol transporter 1; PAD, peripheral arterial disease; PCSK9, proprotein convertase subtilisin/kexin type 9. **Alt text:** Six forest plots showing point estimates and 95% confidence interval bars of associations between genetically predicted lipoprotein(a) and low-density lipoprotein cholesterol lowering and risks of major cardiovascular events. Compared to the reference group, the risk of cardiovascular disease, coronary artery disease, and cardiovascular mortality was lower in the group with genetically predicted lower levels of lipoprotein(a) and low-density lipoprotein cholesterol.

Compared to the reference group, the risk of CVD, CAD, PAD, 3-point MACE, 4-point MACE, and cardiovascular mortality was lower in the group with genetically predicted lower levels of Lp(a) and LDL-C (Figure 4). The HR of overall CVD was 0.92 (95% CI: 0.90, 0.93) for genetically predicted lower levels of Lp(a) and LDL-C via HMGCR target, 0.90 (95% CI: 0.89, 0.92) for genetically predicted lower levels of Lp(a) and LDL-C via NPC1L1 target, 0.90 (95% CI: 0.88, 0.91) for genetically predicted lower levels of Lp(a) and LDL-C via PCSK9 target, and 0.89 (95% CI: 0.87, 0.90) for genetically predicted lower levels of Lp(a) and LDL-C via LDLR target, compared to the reference group.

Interactions were not observed except for PAD outcome when combining lower levels of Lp(a) with LDL-C via LDLR target (HR, 0.71; 95% CI: 0.65, 0.76; *P_interaction_* = 0.006) (Figure 4). This represents a sub-additive interaction, as the estimate for Lp(a) and LDL-C lowering is less than the combination of estimates for Lp(a) and LDL-C lowering. The results for stroke and its subtypes, AF, HF, VTE, and all-cause mortality are shown in Supplementary Figure S2-S6. However, we did not observe any interactions between the two arms in the analyses of these outcomes (*P_interaction_* > 0.05).

### Joint associations of genetically predicted Lp(a) lowering and interventions on BMI, SBP and lifestyles with cardiovascular risk and mortality

**Figure 5:**
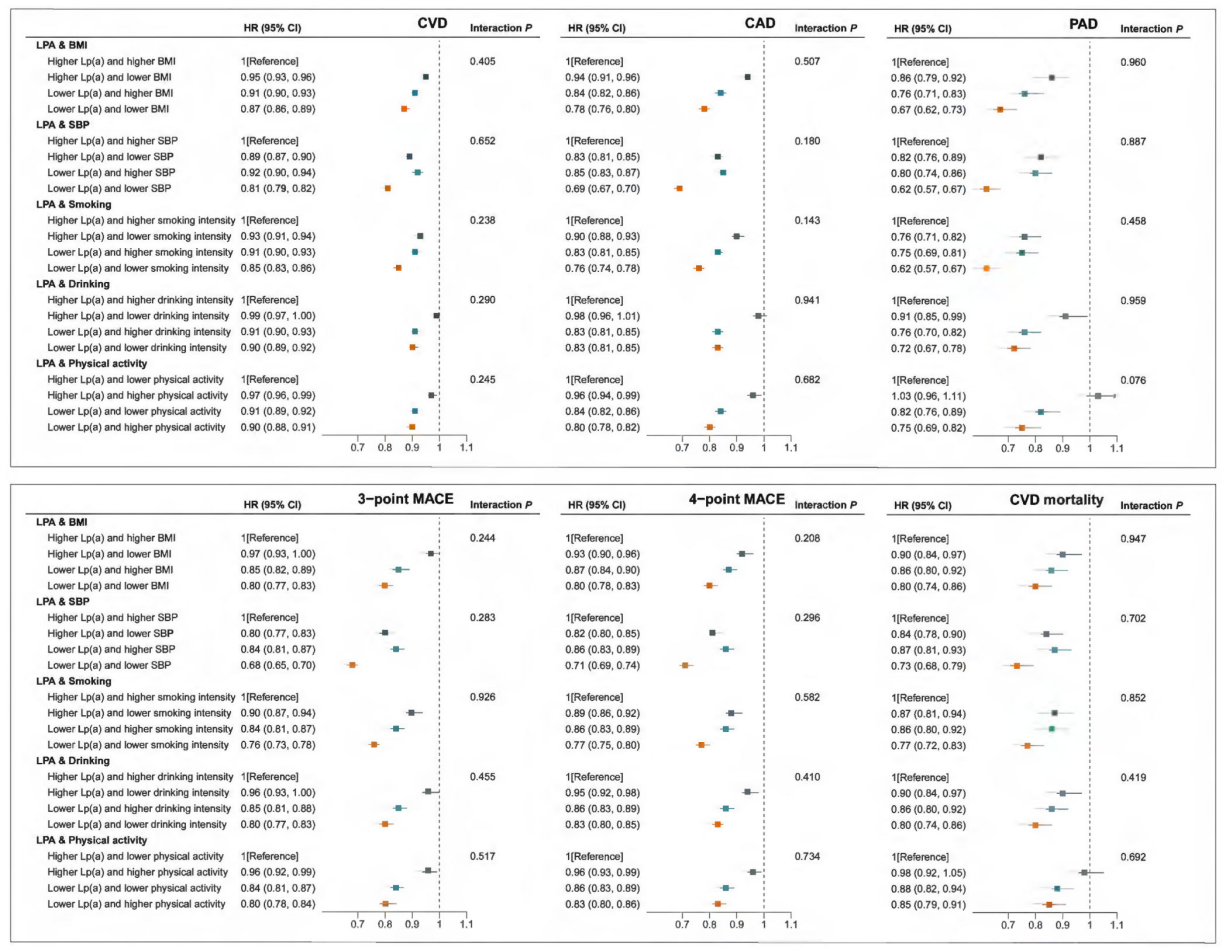
Joint associations of genetically predicted lipoprotein(a) [Lp(a)] lowering and interventions on body mass index (BMI), systolic blood pressure (SBP) and lifestyles with major cardiovascular events. Solid squares represent point estimation, and horizontal lines represent 95% confidence intervals. For each subgroup, cox proportional hazards regression analysis was conducted to estimate the hazard ratio (HR), with adjustment for age, sex, assessment center and the first 10 principal components. The interaction *P* value was calculated by adding genetic scores as continuous variables into the model. BMI, body mass index; CAD, coronary artery disease; CI, confidence interval; CVD, cardiovascular disease; HR, hazard ratio; LPA, lipoprotein(a); Lp(a), lipoprotein(a); MACE, major adverse cardiovascular events; PAD, peripheral arterial disease; SBP, systolic blood pressure. **Alt text:** Six forest plots showing point estimates and 95% confidence interval bars of joint associations between genetically predicted lipoprotein(a) [Lp(a)] lowering and interventions on body mass index, systolic blood pressure and lifestyles and major cardiovascular events. Compared to the reference group, the risk of cardiovascular disease, coronary artery disease, peripheral artery disease, 3- and 4-point major adverse cardiac events was reduced in the group with genetically predicted lower Lp(a) levels and lifestyle improvements.

Compared to the reference group, the risk of CVD, CAD, PAD, 3-point MACE, 4-point MACE, and cardiovascular mortality was reduced in the group with genetically predicted lower Lp(a) levels and lifestyle improvements (Figure 5). For CVD outcome, we observed a HR of 0.87 (95% CI: 0.86, 0.89) for genetically predicted lower Lp(a) levels and lower BMI, 0.81 (95% CI: 0.79, 0.82) for genetically predicted lower Lp(a) levels and lower SBP, 0.85 (95% CI: 0.83, 0.86) for genetically predicted lower Lp(a) levels and lower smoking intensity, 0.90 (95% CI: 0.89, 0.92) for genetically predicted lower Lp(a) levels and lower drinking intensity, and 0.90 (95% CI: 0.88, 0.91) for genetically predicted lower Lp(a) levels and higher physical activity intensity (Figure 5). The similar pattern of the associations was identified for stroke and its subtypes, AF, HF, VTE, and overall mortality that lower risk was observed among the group with genetically predicted lower levels of Lp(a) and lifestyle improvements (Supplementary Figure S7-S11). Again, no interactions were identified between the two arms in the analyses of these outcomes (*P_interaction_* > 0.05).

## Discussion

This study found that genetically predicted lower levels of Lp(a) were associated with a reduced risk of various CVDs, particularly PAD. The factorial MR analysis observed no clear evidence for interactions between Lp(a) lowering and LDL-C lowering or lifestyle improvements in CVD risk reduction, except for the PAD outcome when combining low-level Lp(a) with low-level LDL-C.

The causal potential of the association between Lp(a) and the risk of CVDs has been explored in previous cohort and MR studies and clinical trials.^3, 13, 35–40^ Our findings are in line with these studies in support of a positive association between Lp(a) levels and the risk of CVDs, in particular atherosclerotic outcomes, and therefore potentially beneficial effects on CVD risk reduction after Lp(a) lowering treatment. As far as we know, there is no approved treatment targeting Lp(a). Apolipoprotein(a) antisense oligonucleotide (pelacarsen) has been found to be a promising target for Lp(a) lowering,^15^ and whether it can generate clinical benefits on cardiovasulcar endpoints is unknown and under investigation in a phase 3 clinical trial. ^16^ In addition, whether this approach should be recommended in a general population, like for the primary prevention,^3^ or merely among patients at a high risk^41^ needs to be assessed by comprehensively considering the cost-effectiveness and safety of the treatment.

Polypill strategy including lipid lowering pills, antihypertensive drugs, aspirin, etc., has been found to lower incidence of cardiovascular events compared to the usual care.^42, 43^ For example, the PolyIran study, a two-group, pragmatic, cluster-randomised trial, aimed to assess the effectiveness and safety of a four-component polypill for primary and secondary prevention of cardiovascular disease. This study found that using the polypill could effectively prevent major cardiovascular events, achieve high medication adherence, and result in a low frequency of adverse events.^43^ In addition, some MR studies explored the potential effects of polypill inhibiting PCSK9 and HMGCR,^21^ PCSK9 and CETP,^22^ and HMGCR and NPC1L1^44^ on the risk of developing CVDs, and consistently revealed joint effects of genetically predicted LDL-C lowering treatments with different targets on CVD risk reduction^21, 22, 44^. Our study examined the interactions of Lp(a) lowering with LDL-C lowering via four targets in CVD and generated similar findings that Lp(a) lowering might had generally synergistic effects on CVD risk when combined with LDL-C lowering. Even though effect sizes were incomparable between LDL-C lowering targets due to varying instrument strengths for each LDL-C lowering target, the study supported a potential better combination of genetically predicted lower levels of Lp(a) and LDLR in lowering the levels of apoB, which may drive the associations between non-high-density lipoprotein cholesterol and CVD.^45, 46^

Although the evidence for lifestyle interventions in reducing CVD risk is compelling,^47^ it remains unclear if the combination of medications and lifestyle modifications exhibits a synergistic effect in reducing cardiovascular risk. A cohort study among 41,225 participants found that the initiation of preventive antihypertensive or statin therapy could lead to both favorable and unfavorable lifestyle changes,^48^ indicating that there may exist interaction effects. Given the limited research on the interactions between Lp(a) and lifestyle factors, our study conducted factorial MR, and identified suggestive joint effects of genetically predicted lower levels of Lp(a) in reducing CVD risk when combined with healthy lifestyle factors, particularly lower smoking intensity. Although BMI and SBP are not lifestyle factors, they represent anthropometric and physiological changes that can be greatly influenced by lifestyle, and this study also identified potential interaction effects between Lp(a) reduction and blood pressure lowering or weight loss in modifying CVD risk. These findings imply the importance of advising lifestyle modifications in lowering CVD risk even among individuals receiving Lp(a) lowering therapy.

This study had several strengths. We used genetic variants to proxy the effect of Lp(a) lowering therapies, which can diminish the biases caused by confounding and reverse causality since genetic variants are usually not associated with confounders and unmodifiable by the onset of disease. In addition, we included a wide range of CVD endpoints and mortality to reveal a comprehensive cardiovascular effect of Lp(a) lowering therapies individually or when combined with other treatments. Horizontal pleiotropy (the genetic variants used as the instrumental variable influence the outcome not merely via the exposure) may be minimized for drug targets given that all used genetic instruments were selected from corresponding gene regions for studied therapeutic targets.

Limitations of this study need to be discussed. First, the genetically proxied effects of lowering Lp(a) or LDL-C by targeting *HMGCR, NPC1L1, PCSK9* and *LDLR* genes could only reflect the target pathways, which therefore might confine the comparability of our findings to results of trials since off-target pathways were not considered in the analysis. Second, the genetically predicted effects are small and lifelong, in contrast to the larger effects of shorter duration observed by pharmaceutically inhibiting a target. Third, our analysis included only participants of European ancestry, which confines the generalizability of our findings to the populations with different ethnic backgrounds. Last, some other lifestyle factors, like diet, were not included in the analysis due to no robust genetic instruments for these traits.

## Conclusions

In summary, our study suggests that lowering Lp(a) levels may reduce the risk of various CVDs, as well as cardiovascular and all-cause mortality. Lp(a) lowering may have a sub-additive effect on reducing the risk of PAD when combined with LDL-C lowering. Further clinical studies are warranted to validate our findings among CVD patients.

## Supplementary Material

Supplementary material

Supplementary material

## Figures and Tables

**Table 1:** Baseline characteristics of participants in the UK Biobank.

Baseline characteristic	All participants (n=385,917)
**Sex, n (%)**	
Female	208227 (54.0)
Male	177690 (46.0)
**Age (years), mean (SD)**	56.7 (8.0)
**BMI (kg/m^2^), mean (SD)**	27.4 (4.8)
**Townsend deprivation index,** **mean (SD)**	-1.5 (3.0)
**Smoking status, n (%)**	
Current	40039 (10.4)
Former	136651 (35.4)
Never	207296 (53.7)
Unknown	1931 (0.5)
**Alcohol drinker status, n (%)**	
Current	359366 (93.1)
Former	13353 (3.5)
Never	12246 (3.2)
Unknown	952 (0.2)
**Physical activity, n (%)**	
Light (0-2)	130774 (33.9)
Medium (3-5)	146642 (38.0)
Heavy (6-7)	89607 (23.2)
Unknown	18894 (4.9)
**Blood lipid concentrations (mg/dL), mean (SD)**	
Total cholesterol	220.8 (44.1)
LDL cholesterol	138.0 (33.6)
HDL cholesterol	56.2 (14.8)
Triglycerides	154.9 (90.6)
Apolipoprotein B	103.4 (23.8)
Apolipoprotein A	154.2 (27.1)
Lipoprotein(a)	17.6 (19.7)

BMI, body mass index; HDL, high-density lipoprotein; LDL, low-density lipoprotein; SD, standard deviation.

## Data Availability

This research has been conducted using the UK Biobank Resource under Application Number 10775. Data are available from the UK Biobank (https://www.ukbiobank.ac.uk/) for researchers who meet the criteria and gain approvals to access the research database from the UK Biobank.

## Key messages

- The application of lipoprotein(a) [Lp(a)] lowering treatment for cardiovascular events, as well as its potential interactions with other treatments like low-density lipoprotein cholesterol (LDL-C) lowering therapy and lifestyle improvements, remains uncertain.
- A sub-additive interaction between Lp(a) lowering and LDL-C lowering in reducing the risk of peripheral artery disease (PAD) was observed, however, no clear evidence that the effect of varying Lp(a) in isolation will differ from the effect of varying Lp(a) in conjunction with lifestyle interventions.
- Combination therapy is not recommended for Lp(a) lowering treatment in reducing cardiovascular risk.
