# Supplementary material for: Factorial Mendelian Randomization of Lp(a) Lowering, LDL-C Lowering, and Lifestyle Improvements: Joint Associations with Cardiovascular Risk
Source: Int J Epidemiol. Author manuscript; Available in PMC 2025 Mar 10. (PMC11893152; doi:10.1093/ije/dyaf020)
Supplement: Supplementary material [file EMS203892-supplement-Supplementary_material.pdf]

Supplementary Figures

Supplementary Figure S1. Joint associations of genetically predicted lipoprotein(a) [Lp(a)] and low-density lipoprotein cholesterol (LDL-C) lowering with levels of blood lipids and lipoproteins..3

Supplementary Figure S2. Joint associations of genetically predicted lipoprotein(a) [Lp(a)] and low-density lipoprotein cholesterol (LDL-C) lowering with the risk of stroke and its subtypes..4

Supplementary Figure S3. Joint associations of genetically predicted lipoprotein(a) [Lp(a)] and low-density lipoprotein cholesterol (LDL-C) lowering with the risk of atrial fibrillation...5

Supplementary Figure S4. Joint associations of genetically predicted lipoprotein(a) [Lp(a)] and low-density lipoprotein cholesterol (LDL-C) lowering with the risk of heart failure...6

Supplementary Figure S5. Joint associations of genetically predicted lipoprotein(a) [Lp(a)] and low-density lipoprotein cholesterol (LDL-C) lowering with the risk of venous thromboembolism...7

Supplementary Figure S6. Joint associations of genetically predicted lipoprotein(a) [Lp(a)] and low-density lipoprotein cholesterol (LDL-C) lowering with the risk of all-cause mortality...8

Supplementary Figure S7. Joint associations of genetically predicted lipoprotein(a) [Lp(a)] lowering and interventions on body mass index (BMI), systolic blood pressure (SBP), and lifestyle factors with the risk of stroke and its subtypes...9

Supplementary Figure S8. Joint associations of genetically predicted lipoprotein(a) [Lp(a)] lowering and interventions on body mass index (BMI), systolic blood pressure (SBP), and lifestyle factors with the risk of atrial fibrillation...10

Supplementary Figure S9. Joint associations of genetically predicted lipoprotein(a) [Lp(a)] lowering and interventions on body mass index (BMI), systolic blood pressure (SBP), and lifestyle factors with the risk of heart failure...11

Supplementary Figure S10. Joint associations of genetically predicted lipoprotein(a) [Lp(a)] lowering and interventions on body mass index (BMI), systolic blood pressure (SBP), and lifestyle factors with the risk of venous thromboembolism...12

Supplementary Figure S11. Joint associations of genetically predicted

lipoprotein(a) [Lp(a)] lowering and interventions on body mass index (BMI), systolic blood pressure (SBP), and lifestyle factors with the risk of all-cause mortality...13

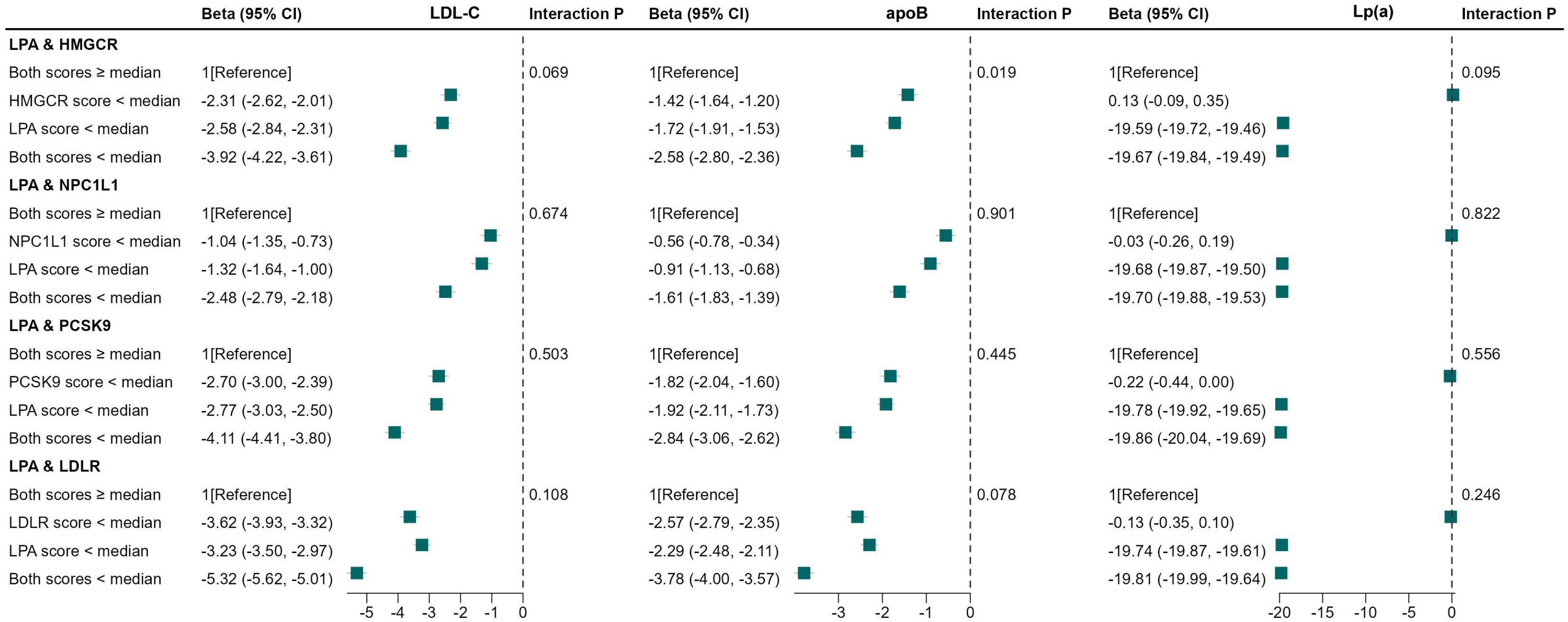

	LDL-C Beta (95% CI)	LDL-C Interaction P	apoB Beta (95% CI)	apoB Interaction P	Lp(a) Beta (95% CI)	Lp(a) Interaction P
**LPA & HMGCR**						
Both scores ≥ median	1[Reference]	0.069	1[Reference]	0.019	1[Reference]	0.095
HMGCR score < median	-2.31 (-2.62, -2.01)		-1.42 (-1.64, -1.20)		0.13 (-0.09, 0.35)	
LPA score < median	-2.58 (-2.84, -2.31)		-1.72 (-1.91, -1.53)		-19.59 (-19.72, -19.46)	
Both scores < median	-3.92 (-4.22, -3.61)		-2.58 (-2.80, -2.36)		-19.67 (-19.84, -19.49)	
**LPA & NPC1L1**						
Both scores ≥ median	1[Reference]	0.674	1[Reference]	0.901	1[Reference]	0.822
NPC1L1 score < median	-1.04 (-1.35, -0.73)		-0.56 (-0.78, -0.34)		-0.03 (-0.26, 0.19)	
LPA score < median	-1.32 (-1.64, -1.00)		-0.91 (-1.13, -0.68)		-19.68 (-19.87, -19.50)	
Both scores < median	-2.48 (-2.79, -2.18)		-1.61 (-1.83, -1.39)		-19.70 (-19.88, -19.53)	
**LPA & PCSK9**						
Both scores ≥ median	1[Reference]	0.503	1[Reference]	0.445	1[Reference]	0.556
PCSK9 score < median	-2.70 (-3.00, -2.39)		-1.82 (-2.04, -1.60)		-0.22 (-0.44, 0.00)	
LPA score < median	-2.77 (-3.03, -2.50)		-1.92 (-2.11, -1.73)		-19.78 (-19.92, -19.65)	
Both scores < median	-4.11 (-4.41, -3.80)		-2.84 (-3.06, -2.62)		-19.86 (-20.04, -19.69)	
**LPA & LDLR**						
Both scores ≥ median	1[Reference]	0.108	1[Reference]	0.078	1[Reference]	0.246
LDLR score < median	-3.62 (-3.93, -3.32)		-2.57 (-2.79, -2.35)		-0.13 (-0.35, 0.10)	
LPA score < median	-3.23 (-3.50, -2.97)		-2.29 (-2.48, -2.11)		-19.74 (-19.87, -19.61)	
Both scores < median	-5.32 (-5.62, -5.01)		-3.78 (-4.00, -3.57)		-19.81 (-19.99, -19.64)	

Supplementary Figure S1. Joint associations of genetically predicted lipoprotein(a) [Lp(a)] and low-density lipoprotein cholesterol (LDL-C) lowering with levels of blood lipids and lipoproteins. Solid squares represent point estimation, and horizontal lines represent 95% confidence intervals. For each subgroup, multivariable linear regression was employed to estimate the effects of genetically predicted lower levels of Lp(a) and LDL-C via any targets on the measured levels of blood lipids and lipoproteins, with adjustment of age, sex, assessment center and the first 10 principal components. The interaction P value was calculated by adding genetic scores as continuous variables into the model. apoB, apolipoprotein B; CI, confidence interval; HMGCR, 3-hydroxy-3-methylglutaryl-CoA reductase; LDL-C, low-density lipoprotein cholesterol; LDLR, low density lipoprotein receptor; LPA, lipoprotein(a); Lp(a), lipoprotein(a); NPC1L1, NPC1 like intracellular cholesterol transporter 1; PCSK9, proprotein convertase subtilisin/kexin type 9.

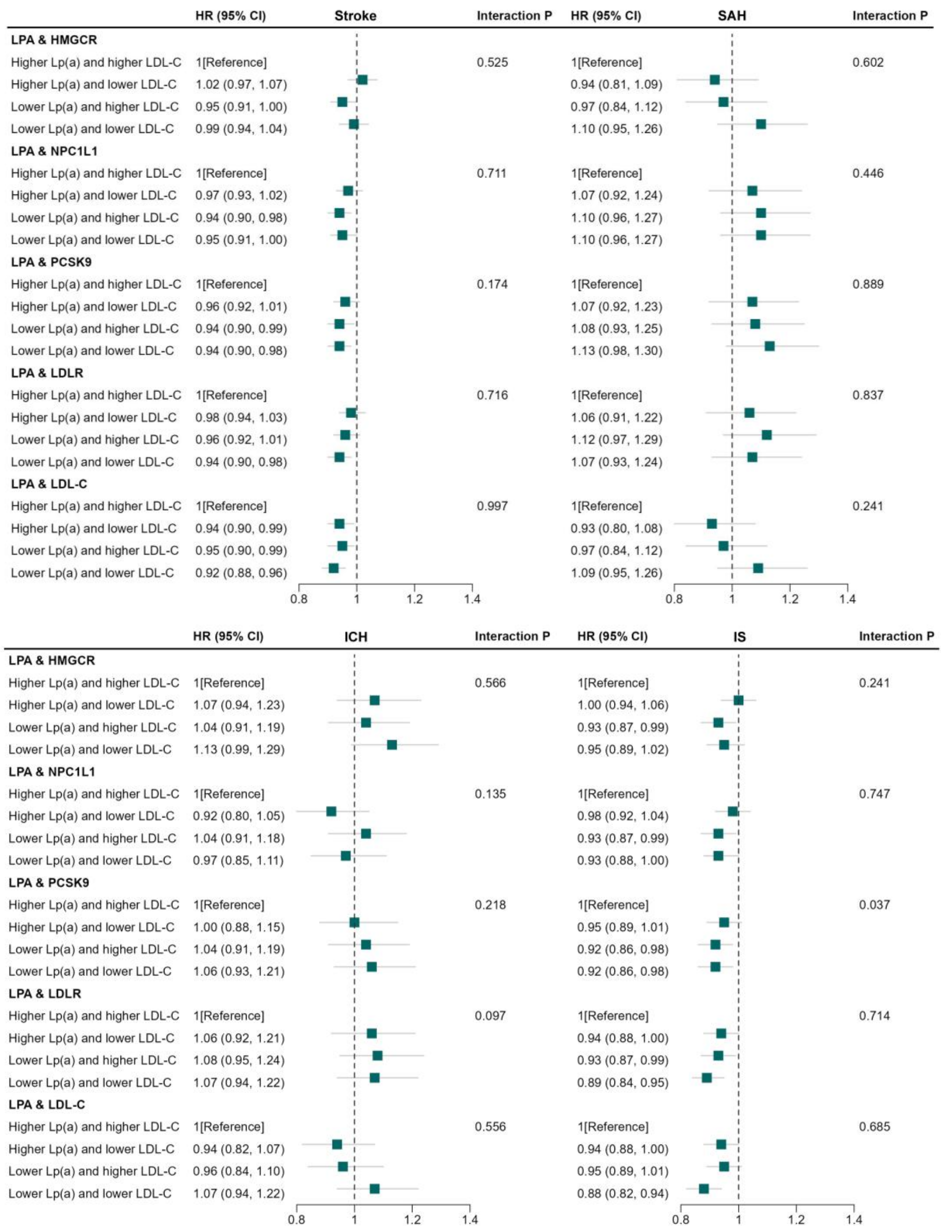

Supplementary Figure S2. Joint associations of genetically predicted lipoprotein(a) [Lp(a)] and low-density lipoprotein cholesterol (LDL-C) lowering with the risk of stroke and its subtypes. Solid squares represent point estimation, and horizontal lines represent 95% confidence intervals. For each subgroup, cox proportional hazards regression analysis was conducted to estimate the hazard ratio (HR), with adjustment for age, sex, assessment center and the first 10 principal components. The interaction P value was calculated by adding genetic scores as continuous variables into the model. CI, confidence interval; HMGCR, 3-hydroxy-3-methylglutaryl-CoA reductase; HR, hazard ratio; ICH, intracerebral hemorrhage; IS, ischemic stroke; LDL-C, low-density lipoprotein cholesterol; LDLR, low density lipoprotein receptor; LPA, lipoprotein(a); Lp(a), lipoprotein(a); NPC1L1, NPC1 like intracellular cholesterol transporter 1; PCSK9, proprotein convertase subtilisin/kexin type 9; SAH, subarachnoid hemorrhage.

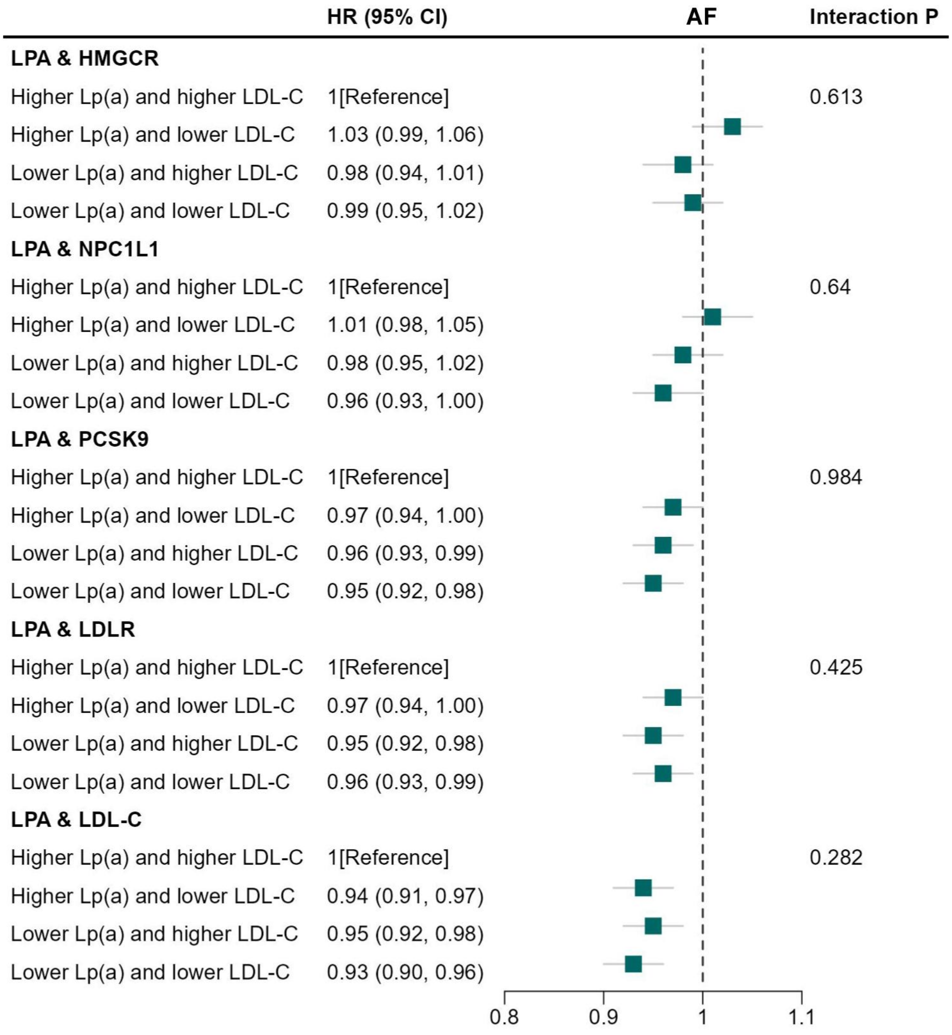

Supplementary Figure S3. Joint associations of genetically predicted lipoprotein(a) [Lp(a)] and low-density lipoprotein cholesterol (LDL-C) lowering with the risk of atrial fibrillation. Solid squares represent point estimation, and horizontal lines represent 95% confidence intervals. For each subgroup, cox proportional hazards regression analysis was conducted to estimate the hazard ratio (HR), with adjustment for age, sex, assessment center and the first 10 principal components. The interaction P value was calculated by adding genetic scores as continuous variables into the model. AF, atrial fibrillation; CI, confidence interval; HMGCR, 3-hydroxy-3-methylglutaryl-CoA reductase; HR, hazard ratio; LDL-C, low-density lipoprotein cholesterol; LDLR, low density lipoprotein receptor; LPA, lipoprotein(a); Lp(a), lipoprotein(a); NPC1L1, NPC1 like intracellular cholesterol transporter 1; PCSK9, proprotein convertase subtilisin/kexin type 9.

	HR (95% CI)	Interaction P
**LPA & HMGCR**		
Higher Lp(a) and higher LDL-C	1[Reference]	0.863
Higher Lp(a) and lower LDL-C	1.03 (0.98, 1.07)	
Lower Lp(a) and higher LDL-C	0.91 (0.87, 0.96)	
Lower Lp(a) and lower LDL-C	0.94 (0.90, 0.99)	
**LPA & NPC1L1**		
Higher Lp(a) and higher LDL-C	1[Reference]	0.526
Higher Lp(a) and lower LDL-C	0.98 (0.93, 1.02)	
Lower Lp(a) and higher LDL-C	0.93 (0.88, 0.97)	
Lower Lp(a) and lower LDL-C	0.88 (0.84, 0.93)	
**LPA & PCSK9**		
Higher Lp(a) and higher LDL-C	1[Reference]	0.642
Higher Lp(a) and lower LDL-C	1.01 (0.97, 1.06)	
Lower Lp(a) and higher LDL-C	0.93 (0.88, 0.97)	
Lower Lp(a) and lower LDL-C	0.92 (0.87, 0.96)	
**LPA & LDLR**		
Higher Lp(a) and higher LDL-C	1[Reference]	0.147
Higher Lp(a) and lower LDL-C	0.95 (0.91, 1.00)	
Lower Lp(a) and higher LDL-C	0.90 (0.86, 0.94)	
Lower Lp(a) and lower LDL-C	0.89 (0.84, 0.93)	
**LPA & LDL-C**		
Higher Lp(a) and higher LDL-C	1[Reference]	0.77
Higher Lp(a) and lower LDL-C	0.89 (0.85, 0.94)	
Lower Lp(a) and higher LDL-C	0.89 (0.85, 0.93)	
Lower Lp(a) and lower LDL-C	0.84 (0.81, 0.88)	

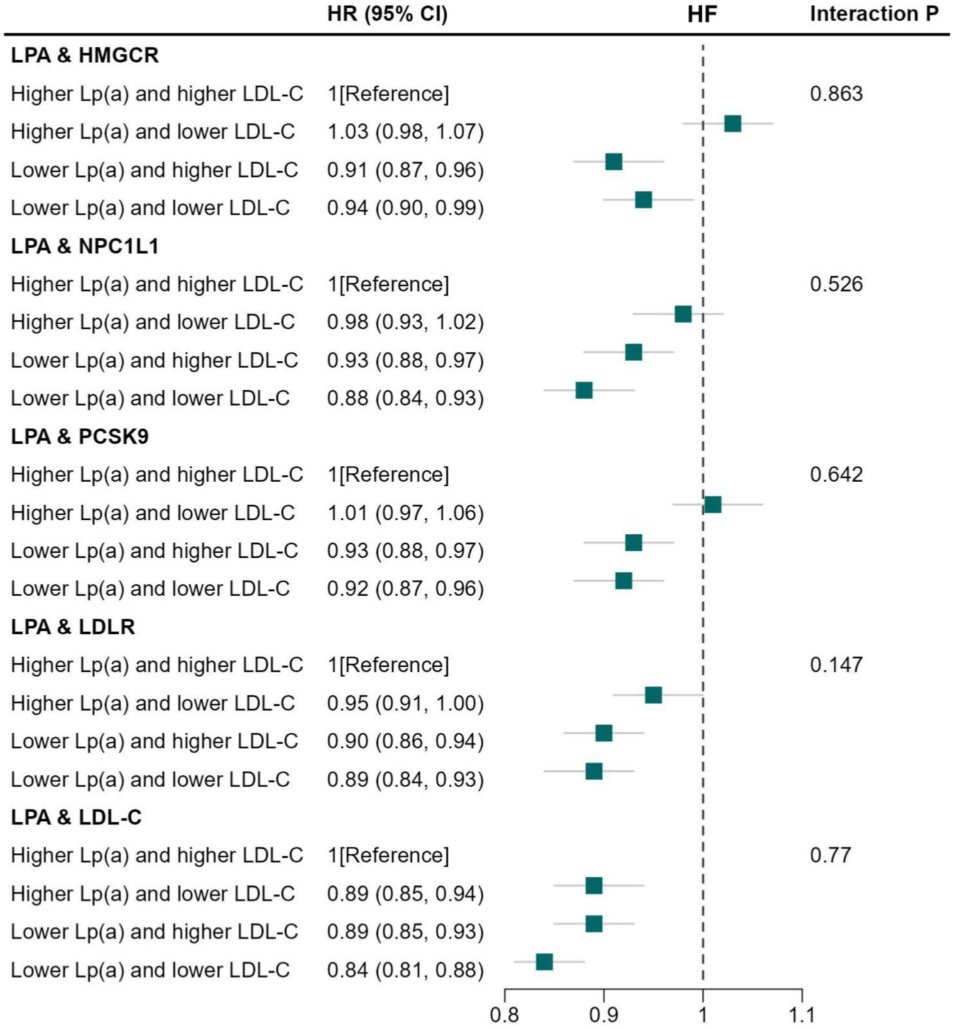

Supplementary Figure S4. Joint associations of genetically predicted lipoprotein(a) [Lp(a)] and low-density lipoprotein cholesterol (LDL-C) lowering with the risk of heart failure. Solid squares represent point estimation, and horizontal lines represent 95% confidence intervals. For each subgroup, cox proportional hazards regression analysis was conducted to estimate the hazard ratio (HR), with adjustment for age, sex, assessment center and the first 10 principal components. The interaction P value was calculated by adding genetic scores as continuous variables into the model. CI, confidence interval; HF, heart failure; HMGCR, 3-hydroxy-3-methylglutaryl-CoA reductase; HR, hazard ratio; LDL-C, low-density lipoprotein cholesterol; LDLR, low density lipoprotein receptor; LPA, lipoprotein(a); Lp(a), lipoprotein(a); NPC1L1, NPC1 like intracellular cholesterol transporter 1; PCSK9, proprotein convertase subtilisin/kexin type 9.

	HR (95% CI)	Interaction P
**LPA & HMGCR**		
Higher Lp(a) and higher LDL-C	1[Reference]	0.779
Higher Lp(a) and lower LDL-C	0.99 (0.94, 1.04)	
Lower Lp(a) and higher LDL-C	0.96 (0.91, 1.01)	
Lower Lp(a) and lower LDL-C	0.96 (0.91, 1.01)	
**LPA & NPC1L1**		
Higher Lp(a) and higher LDL-C	1[Reference]	0.58
Higher Lp(a) and lower LDL-C	1.00 (0.95, 1.05)	
Lower Lp(a) and higher LDL-C	0.95 (0.90, 1.00)	
Lower Lp(a) and lower LDL-C	0.97 (0.92, 1.03)	
**LPA & PCSK9**		
Higher Lp(a) and higher LDL-C	1[Reference]	0.785
Higher Lp(a) and lower LDL-C	0.96 (0.91, 1.01)	
Lower Lp(a) and higher LDL-C	0.94 (0.89, 0.99)	
Lower Lp(a) and lower LDL-C	0.95 (0.90, 1.00)	
**LPA & LDLR**		
Higher Lp(a) and higher LDL-C	1[Reference]	0.984
Higher Lp(a) and lower LDL-C	0.93 (0.88, 0.98)	
Lower Lp(a) and higher LDL-C	0.95 (0.90, 1.00)	
Lower Lp(a) and lower LDL-C	0.90 (0.86, 0.95)	
**LPA & LDL-C**		
Higher Lp(a) and higher LDL-C	1[Reference]	0.58
Higher Lp(a) and lower LDL-C	0.96 (0.91, 1.01)	
Lower Lp(a) and higher LDL-C	0.96 (0.91, 1.01)	
Lower Lp(a) and lower LDL-C	0.93 (0.88, 0.98)	

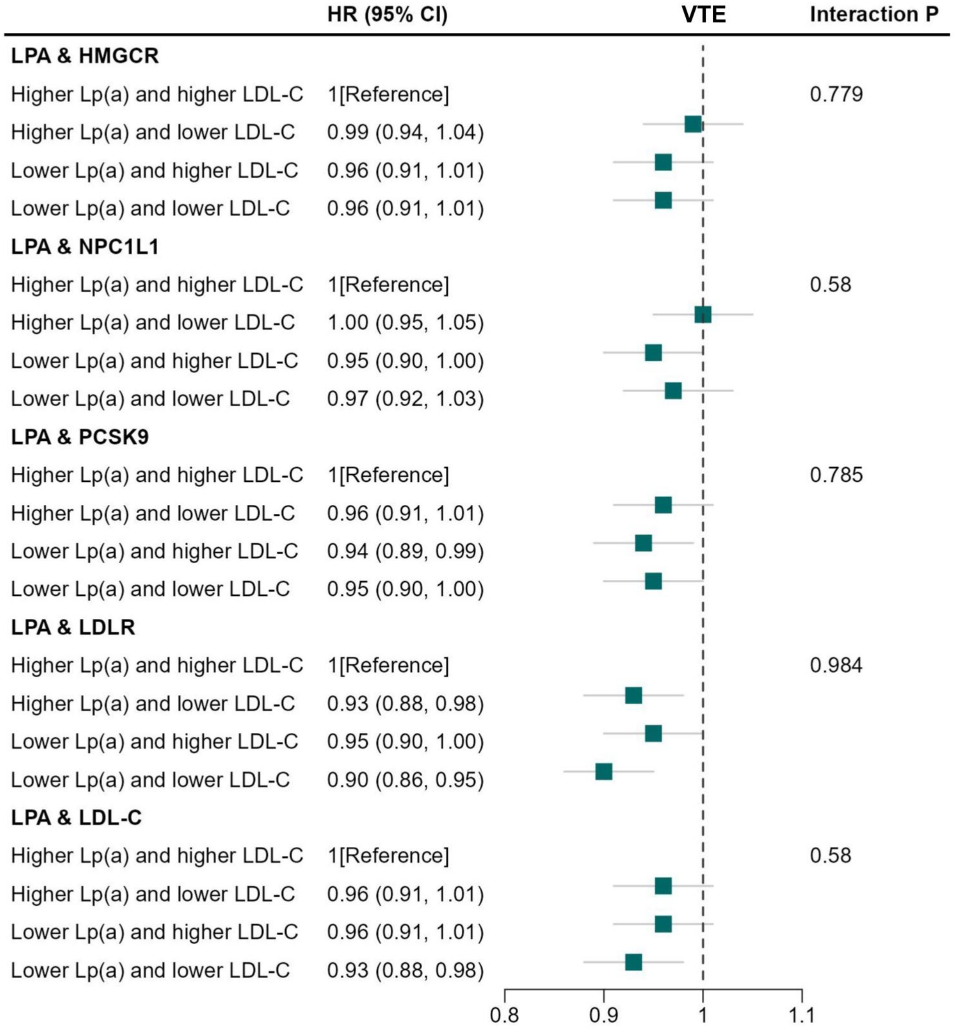

Supplementary Figure S5. Joint associations of genetically predicted lipoprotein(a) [Lp(a)] and low-density lipoprotein cholesterol (LDL-C) lowering with the risk of venous thromboembolism. Solid squares represent point estimation, and horizontal lines represent 95% confidence intervals. For each subgroup, cox proportional hazards regression analysis was conducted to estimate the hazard ratio (HR), with adjustment for age, sex, assessment center and the first 10 principal components. The interaction P value was calculated by adding genetic scores as continuous variables into the model. CI, confidence interval; HMGCR, 3-hydroxy-3-methylglutaryl-CoA reductase; HR, hazard ratio; LDL-C, low-density lipoprotein cholesterol; LDLR, low density lipoprotein receptor; LPA, lipoprotein(a); Lp(a), lipoprotein(a); NPC1L1, NPC1 like intracellular cholesterol transporter 1; PCSK9, proprotein convertase subtilisin/kexin type 9; VTE, venous thromboembolism.

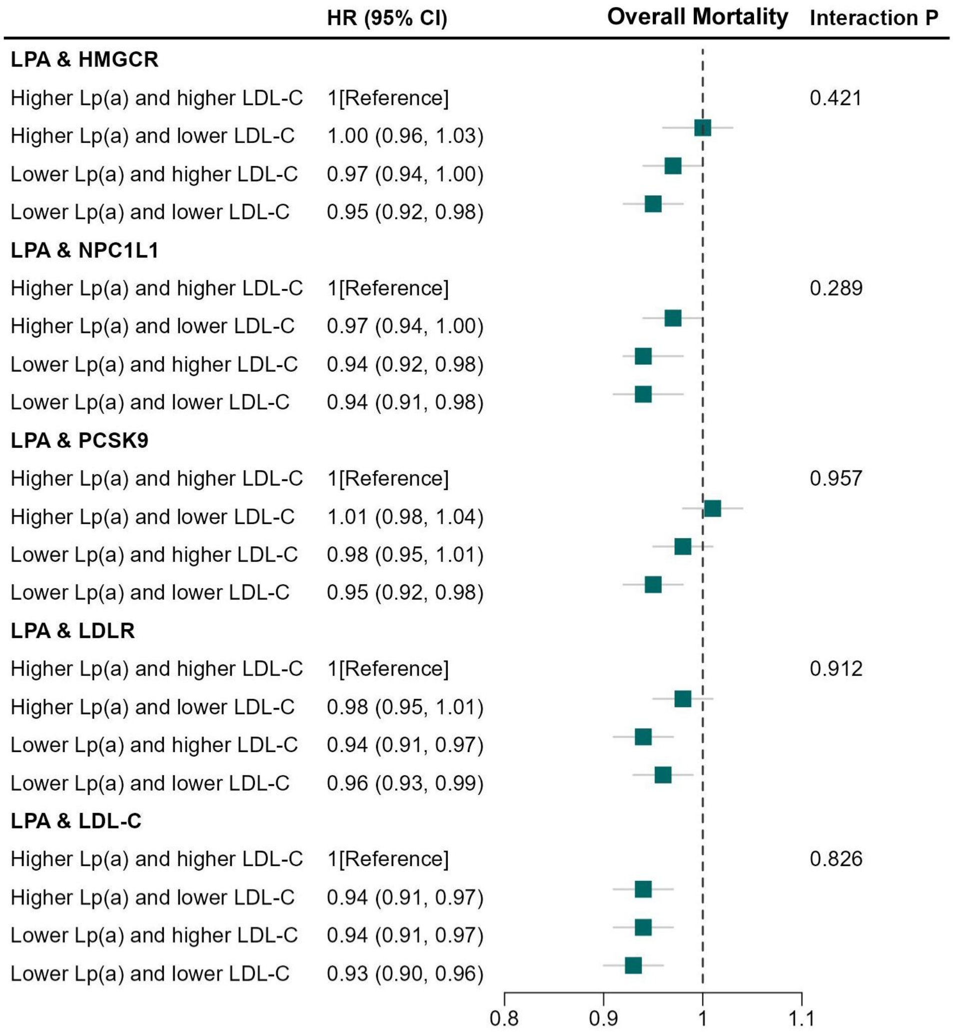

Supplementary Figure S6. Joint associations of genetically predicted lipoprotein(a) [Lp(a)] and low-density lipoprotein cholesterol (LDL-C) lowering with the risk of all-cause mortality. Solid squares represent point estimation, and horizontal lines represent 95% confidence intervals. For each subgroup, cox proportional hazards regression analysis was conducted to estimate the hazard ratio (HR), with adjustment for age, sex, assessment center and the first 10 principal components. The interaction P value was calculated by adding genetic scores as continuous variables into the model. CI, confidence interval; HMGCR, 3-hydroxy-3-methylglutaryl-CoA reductase; HR, hazard ratio; LDL-C, low-density lipoprotein cholesterol; LDLR, low density lipoprotein receptor; LPA, lipoprotein(a); Lp(a), lipoprotein(a); NPC1L1, NPC1 like intracellular cholesterol transporter 1; PCSK9, proprotein convertase subtilisin/kexin type 9.

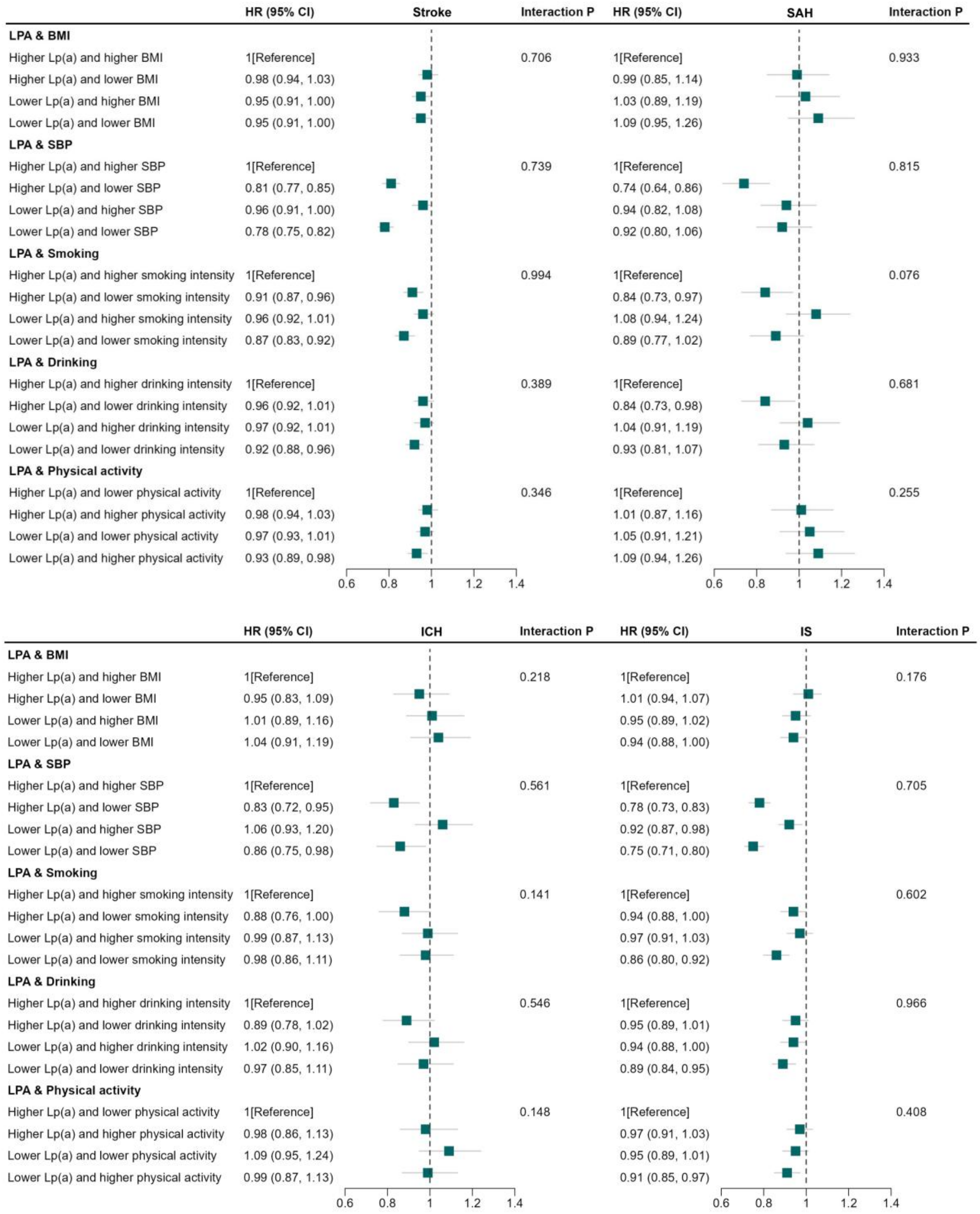

Supplementary Figure S7. Joint associations of genetically predicted lipoprotein(a) [Lp(a)] lowering and interventions on body mass index (BMI), systolic blood pressure (SBP), and lifestyle factors with the risk of stroke and its subtypes. Solid squares represent point estimation, and horizontal lines represent 95% confidence intervals. For each subgroup, cox proportional hazards regression analysis was conducted to estimate the hazard ratio (HR), with adjustment for age, sex, assessment center and the first 10 principal components. The interaction P value was calculated by adding genetic scores as continuous variables into the model. BMI, body mass index; CI, confidence interval; HR, hazard ratio; ICH, intracerebral hemorrhage; IS, ischemic stroke; LPA, lipoprotein(a); Lp(a), lipoprotein(a); SAH, subarachnoid hemorrhage; SBP, systolic blood pressure.

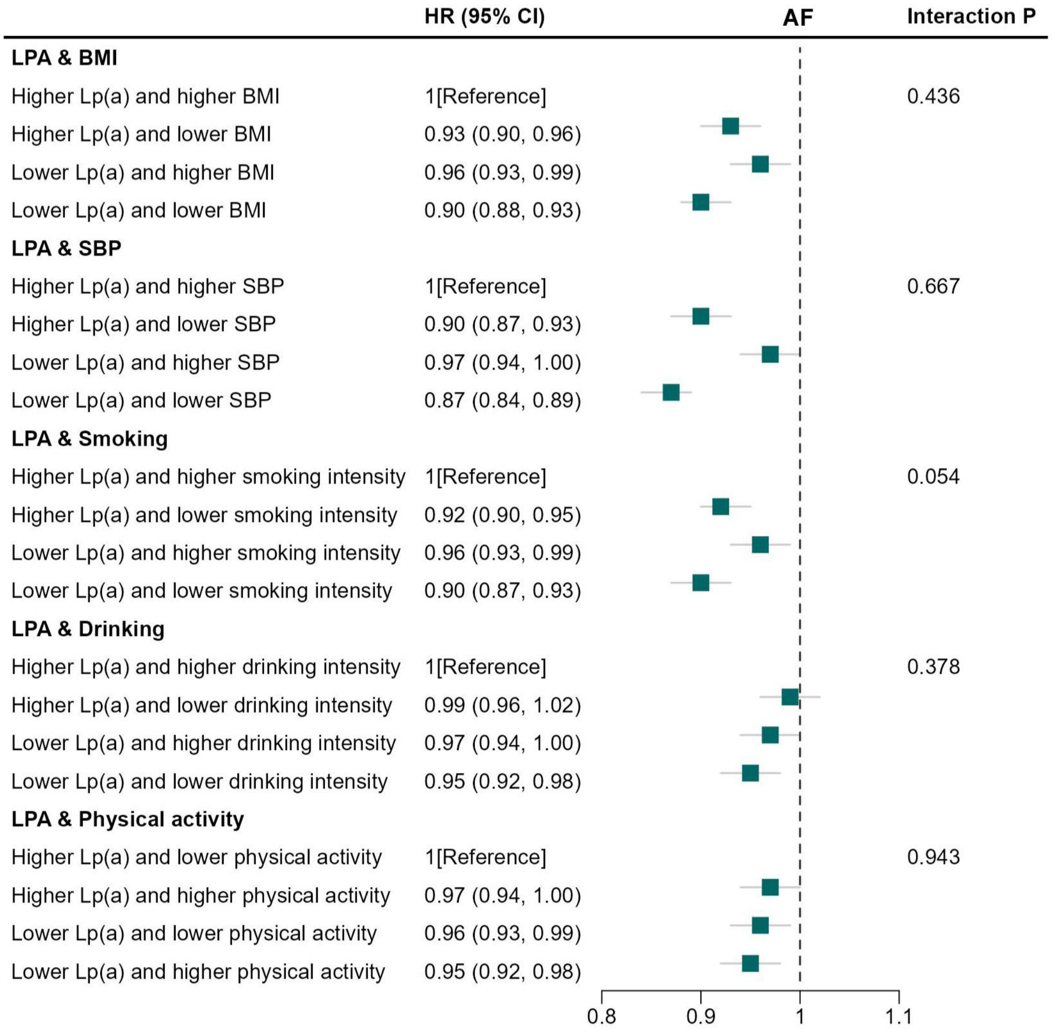

Supplementary Figure S8. Joint associations of genetically predicted lipoprotein(a) [Lp(a)] lowering and interventions on body mass index (BMI), systolic blood pressure (SBP), and lifestyle factors with the risk of atrial fibrillation. Solid squares represent point estimation, and horizontal lines represent 95% confidence intervals. For each subgroup, cox proportional hazards regression analysis was conducted to estimate the hazard ratio (HR), with adjustment for age, sex, assessment center and the first 10 principal components. The interaction P value was calculated by adding genetic scores as continuous variables into the model. AF, atrial fibrillation; BMI, body mass index; CI, confidence interval; HR, hazard ratio; LPA, lipoprotein(a); Lp(a), lipoprotein(a); SBP, systolic blood pressure.

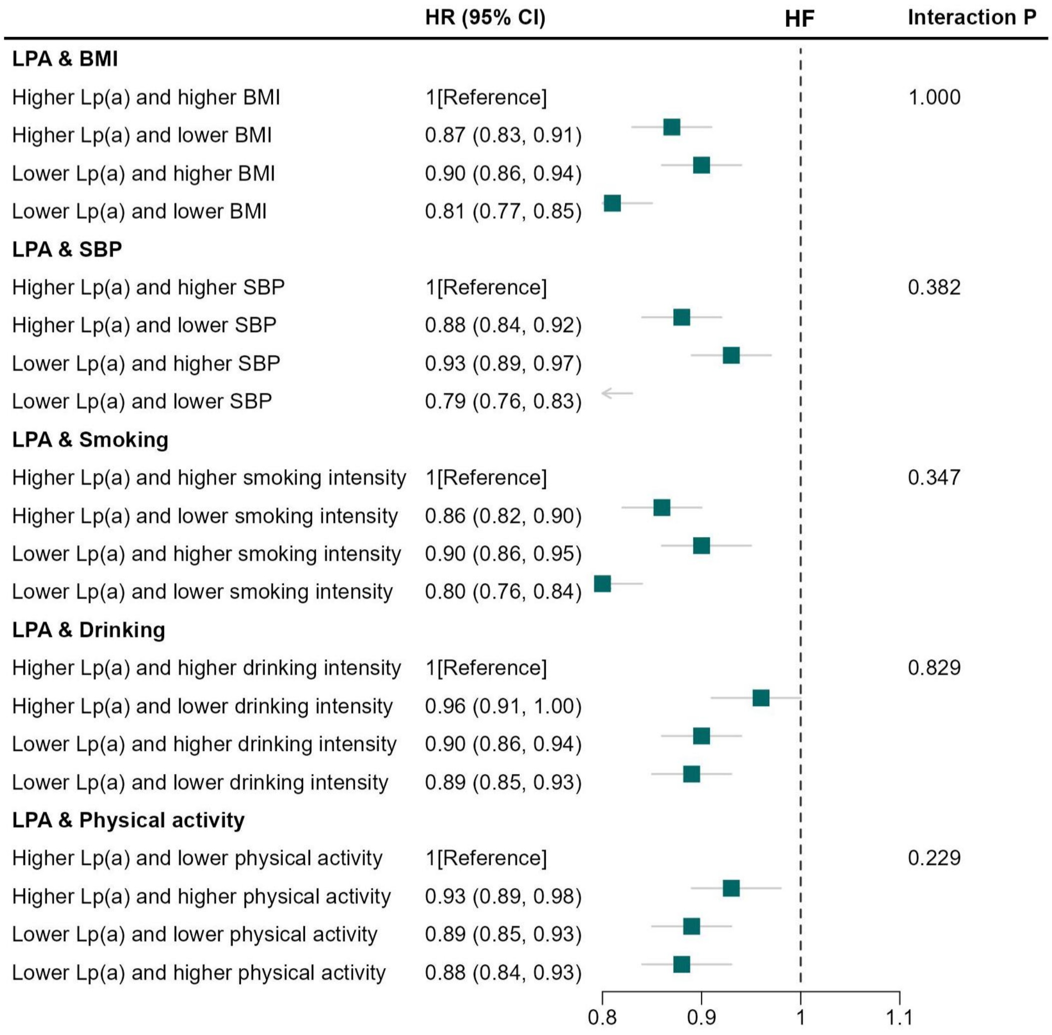

Supplementary Figure S9. Joint associations of genetically predicted lipoprotein(a) [Lp(a)] lowering and interventions on body mass index (BMI), systolic blood pressure (SBP), and lifestyle factors with the risk of heart failure. Solid squares represent point estimation, and horizontal lines represent 95% confidence intervals. For each subgroup, cox proportional hazards regression analysis was conducted to estimate the hazard ratio (HR), with adjustment for age, sex, assessment center and the first 10 principal components. The interaction P value was calculated by adding genetic scores as continuous variables into the model. BMI, body mass index; CI, confidence interval; HF, heart failure; HR, hazard ratio; LPA, lipoprotein(a); Lp(a), lipoprotein(a); SBP, systolic blood pressure.

	HR (95% CI)	Interaction P
**LPA & BMI**		
Higher Lp(a) and higher BMI	1[Reference]	0.202
Higher Lp(a) and lower BMI	0.91 (0.87, 0.96)	
Lower Lp(a) and higher BMI	0.96 (0.91, 1.01)	
Lower Lp(a) and lower BMI	0.88 (0.84, 0.93)	
**LPA & SBP**		
Higher Lp(a) and higher SBP	1[Reference]	0.024
Higher Lp(a) and lower SBP	1.04 (0.98, 1.09)	
Lower Lp(a) and higher SBP	1.01 (0.96, 1.07)	
Lower Lp(a) and lower SBP	0.95 (0.90, 1.00)	
**LPA & Smoking**		
Higher Lp(a) and higher smoking intensity	1[Reference]	0.252
Higher Lp(a) and lower smoking intensity	0.87 (0.83, 0.92)	
Lower Lp(a) and higher smoking intensity	0.96 (0.91, 1.01)	
Lower Lp(a) and lower smoking intensity	0.84 (0.80, 0.89)	
**LPA & Drinking**		
Higher Lp(a) and higher drinking intensity	1[Reference]	0.735
Higher Lp(a) and lower drinking intensity	1.02 (0.97, 1.08)	
Lower Lp(a) and higher drinking intensity	0.95 (0.90, 1.01)	
Lower Lp(a) and lower drinking intensity	1.00 (0.94, 1.05)	
**LPA & Physical activity**		
Higher Lp(a) and lower physical activity	1[Reference]	0.984
Higher Lp(a) and higher physical activity	1.01 (0.96, 1.06)	
Lower Lp(a) and lower physical activity	0.97 (0.92, 1.02)	
Lower Lp(a) and higher physical activity	0.96 (0.91, 1.02)	

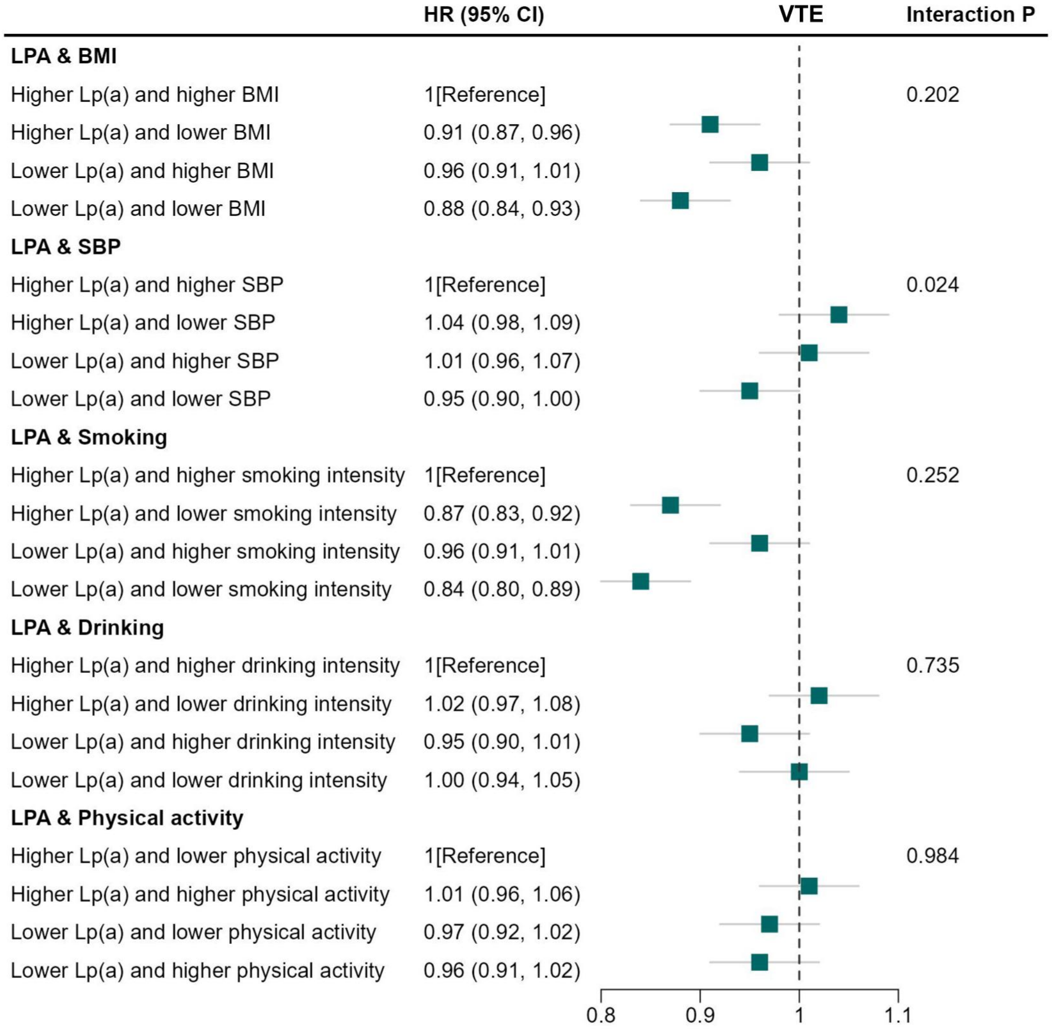

Supplementary Figure S10. Joint associations of genetically predicted lipoprotein(a) [Lp(a)] lowering and interventions on body mass index (BMI), systolic blood pressure (SBP), and lifestyle factors with the risk of venous thromboembolism. Solid squares represent point estimation, and horizontal lines represent 95% confidence intervals. For each subgroup, cox proportional hazards regression analysis was conducted to estimate the hazard ratio (HR), with adjustment for age, sex, assessment center and the first 10 principal components. The interaction P value was calculated by adding genetic scores as continuous variables into the model. BMI, body mass index; CI, confidence interval; HR, hazard ratio; LPA, lipoprotein(a); Lp(a), lipoprotein(a); SBP, systolic blood pressure; VTE, venous thromboembolism.

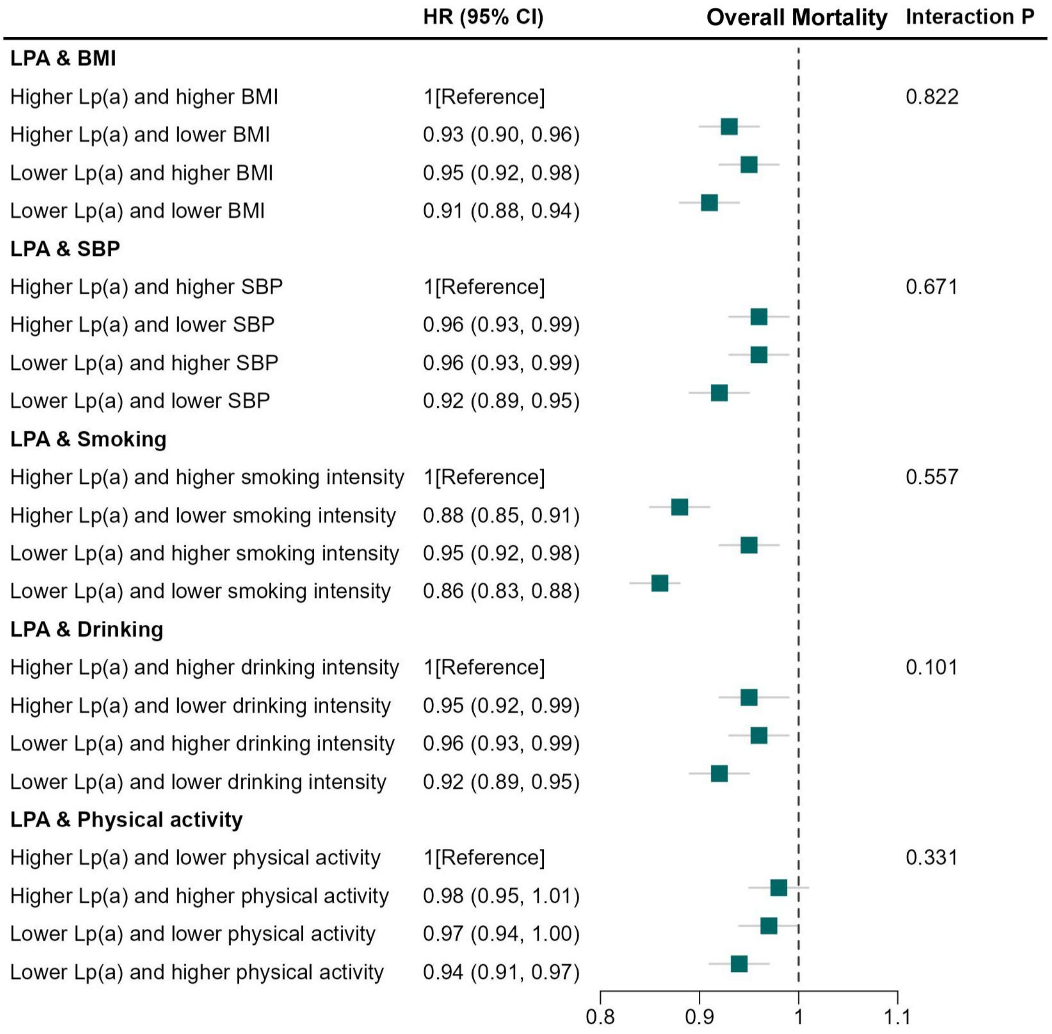

Supplementary Figure S11. Joint associations of genetically predicted lipoprotein(a) [Lp(a)] lowering and interventions on body mass index (BMI), systolic blood pressure (SBP), and lifestyle factors with the risk of all-cause mortality. Solid squares represent point estimation, and horizontal lines represent 95% confidence intervals. For each subgroup, cox proportional hazards regression analysis was conducted to estimate the hazard ratio (HR), with adjustment for age, sex, assessment center and the first 10 principal components. The interaction P value was calculated by adding genetic scores as continuous variables into the model. BMI, body mass index; CI, confidence interval; HR, hazard ratio; LPA, lipoprotein(a); Lp(a), lipoprotein(a); SBP, systolic blood pressure.
