# Supplementary material for: Factorial Mendelian Randomization of Lp(a) Lowering, LDL-C Lowering, and Lifestyle Improvements: Joint Associations with Cardiovascular Risk
Source: Int J Epidemiol. Author manuscript; Available in PMC 2025 Mar 10. (PMC11893152; doi:10.1093/ije/dyaf020)
Supplement: Supplementary material [file EMS203892-supplement-Supplementary_material.docx]

**Supplementary Tables**

**Supplementary Table S1.** Definitions of studied outcomes.............................2

**Supplementary Table S2.** Genetic variants included in lipoprotein(a) [Lp(a)] genetic score.....................................................................................................3

**Supplementary Table S3.** Drug target variants included in the genetic score and their associations with low-density lipoprotein cholesterol (LDL-C) levels in the Global Lipids Genetics Consortium.............................................................5

**Supplementary Table S4.** Genetic variants included in low-density lipoprotein cholesterol (LDL-C) genetic score.....................................................................9

**Supplementary Table S5.** Detailed information about genetic instruments used to proxy body mass index (BMI), systolic blood pressure (SBP), and lifestyle factors................................................................................................15

**Supplementary Table S6.** Genetic instruments used to calculate genetic scores for body mass index (BMI), systolic blood pressure (SBP), and lifestyle factors.............................................................................................................16

**Supplementary Table S1.** Definitions of studied outcomes.

| **Outcome** | **ICD-9** | **ICD-10** |
| --- | --- | --- |
| Cardiovascular disease (CVD) | 390-398, 410-417, 420-438, 440, 443, 444 | I00-I09, I20-I70, I73-I74 |
| Coronary artery disease (CAD) | 410-415, 428, 429 | I20-25 |
| Peripheral arterial disease (PAD) | 443 | I731, I738, I739 |
| Stroke | 430, 431, 434, 436 | I60-I63 |
| Subarachnoid hemorrhage (SAH) | 430 | I60 |
| Intracerebral hemorrhage (ICH) | 431 | I61 |
| Ischemic stroke (IS) | 434, 436 | I63, I64 |
| Atrial fibrillation (AF) | 427.3 | I48 |
| Heart failure (HF) | 428 | I50 |
| Venous thromboembolism (VTE) | 415.1, 444 | I26, I74 |
| Cardiovascular-specific death | 390-398, 410-417, 420-438, 440, 443, 444 | I00-I09, I20-I70, I73-I74 |
| 3-point major adverse cardiovascular events (MACE) | 410, 430, 431-434, 436 | I00-I09, I20-I70, I73-I74 |
| 4-point major adverse cardiovascular events (MACE) | 410, 428, 430, 431-434, 436 | I00-I09, I20-I70, I73-I74 |

**Supplementary Table S2.** Genetic variants included in lipoprotein(a) [Lp(a)] genetic score.

| **SNP** | **Chr** | **Position** | **EA** | **NEA** | **Beta** | **SE** |
| --- | --- | --- | --- | --- | --- | --- |
| rs200144324 | 6 | 160493099 | T | C | 81.5 | 11.3 |
| rs200684404 | 6 | 160543317 | T | C | 67.7 | 9.2 |
| rs183815886 | 6 | 160720804 | C | G | 14.8 | 1.8 |
| rs143365644 | 6 | 160825930 | T | A | 3.7 | 0.5 |
| rs151135411 | 6 | 160831796 | A | G | 69.5 | 2.7 |
| rs117446263 | 6 | 160847571 | A | G | -5.2 | 0.6 |
| rs6916433 | 6 | 160890350 | T | A | -4.7 | 0.3 |
| rs182443492 | 6 | 160891897 | A | C | 36.8 | 1.0 |
| rs139609547 | 6 | 160899049 | - | A | 4.4 | 0.4 |
| rs141834709 | 6 | 160909667 | A | T | 8.7 | 1.0 |
| rs142126734 | 6 | 160942926 | A | G | 7.5 | 0.5 |
| rs41266379 | 6 | 160953137 | C | T | 7.1 | 0.7 |
| rs41267809 | 6 | 160953642 | G | A | -6.6 | 0.6 |
| rs143461353 | 6 | 160954800 | T | C | 13.1 | 1.0 |
| rs139145675 | 6 | 160966559 | A | G | -22.5 | 2.4 |
| rs74617384 | 6 | 160997118 | T | A | 42.4 | 0.5 |
| rs41267813 | 6 | 160998199 | A | G | -58.8 | 2.9 |
| rs200376184 | 6 | 161011999 | C | G | 17.5 | 2.7 |
| rs9456551 | 6 | 161012805 | C | T | 3.6 | 0.2 |
| rs140570886 | 6 | 161013013 | C | T | 80.2 | 0.8 |
| rs73596816 | 6 | 161017363 | A | G | 19.2 | 0.6 |
| rs41259144 | 6 | 161022107 | T | C | -9.6 | 0.8 |
| rs191690882 | 6 | 161031132 | A | G | -13.2 | 1.9 |
| rs369686024 | 6 | 161032800 | A | G | 19.2 | 0.8 |
| rs41269876 | 6 | 161070653 | A | C | -8.2 | 0.6 |
| rs79246098 | 6 | 161078894 | C | T | 6.2 | 0.9 |
| rs77337569 | 6 | 161087652 | G | T | 5.2 | 0.8 |
| rs75274517 | 6 | 161088956 | A | G | -6.5 | 1.0 |
| rs56393506 | 6 | 161089307 | T | C | 12.4 | 0.4 |
| rs139389770 | 6 | 161135746 | G | T | -5.2 | 0.9 |
| rs783147 | 6 | 161137990 | A | G | -2.0 | 0.3 |
| rs4252152 | 6 | 161159366 | G | T | 9.1 | 0.9 |
| rs1835346 | 6 | 161162290 | G | A | 5.2 | 0.7 |
| rs4252170 | 6 | 161162406 | C | T | 3.2 | 0.4 |
| rs117534432 | 6 | 161177443 | T | C | 3.3 | 0.5 |
| rs11753588 | 6 | 161189071 | A | G | -2.4 | 0.3 |
| rs186418835 | 6 | 161214526 | A | G | -9.7 | 1.5 |
| rs140606700 | 6 | 161250301 | G | A | 6.4 | 1.2 |
| rs138491411 | 6 | 161251940 | G | A | 5.0 | 0.8 |
| rs182349273 | 6 | 161255668 | G | A | 34.4 | 5.7 |
| rs34371670 | 6 | 161257953 | T | C | -8.4 | 0.7 |
| rs4709474 | 6 | 161285760 | G | A | 1.7 | 0.2 |
| rs145099029 | 6 | 161292838 | C | A | 17.8 | 1.8 |

EA, effect allele; NEA, non-effect allele; SE, standard error; SNP, single nucleotide polymorphism.

**Supplementary Table S3.** Drug target variants included in the genetic score and their associations with low-density lipoprotein cholesterol (LDL-C) levels in the Global Lipids Genetics Consortium.

| **Target** | **SNP** | **Chr** | **Position** | **EA** | **NEA** | **Beta** | **SE** | **P value** |
| --- | --- | --- | --- | --- | --- | --- | --- | --- |
| HMGCR | rs1308220 | 5 | 74537301 | G | A | 0.049 | 0.006 | 5.55E-14 |
| HMGCR | rs11741997 | 5 | 74560542 | G | A | -0.053 | 0.008 | 6.14E-09 |
| HMGCR | rs2006760 | 5 | 74562029 | G | C | 0.048 | 0.002 | 4.33E-90 |
| HMGCR | rs115845757 | 5 | 74563700 | A | G | 0.063 | 0.006 | 6.18E-19 |
| HMGCR | rs147694838 | 5 | 74578124 | T | A | 0.042 | 0.006 | 7.62E-11 |
| HMGCR | rs4703665 | 5 | 74602898 | T | C | -0.034 | 0.003 | 2.55E-25 |
| HMGCR | rs35122945 | 5 | 74610293 | C | A | -0.043 | 0.004 | 1.85E-25 |
| HMGCR | rs141642272 | 5 | 74615209 | C | G | 0.080 | 0.006 | 1.86E-33 |
| HMGCR | rs111353455 | 5 | 74623949 | A | G | 0.019 | 0.003 | 1.28E-08 |
| HMGCR | rs75240579 | 5 | 74624484 | T | C | -0.048 | 0.005 | 1.27E-18 |
| HMGCR | rs112672253 | 5 | 74628706 | T | A | 0.065 | 0.010 | 1.06E-08 |
| HMGCR | rs17244939 | 5 | 74631096 | C | A | -0.057 | 0.008 | 4.01E-09 |
| HMGCR | rs115169875 | 5 | 74633264 | A | G | -0.039 | 0.006 | 1.22E-09 |
| HMGCR | rs12916 | 5 | 74656539 | C | T | 0.072 | 0.002 | 1.94E-314 |
| HMGCR | rs151264833 | 5 | 74660755 | G | C | 0.036 | 0.006 | 1.11E-08 |
| HMGCR | rs62366588 | 5 | 74664987 | A | C | -0.043 | 0.004 | 6.84E-22 |
| HMGCR | rs17562686 | 5 | 74668589 | T | C | 0.056 | 0.003 | 6.50E-73 |
| HMGCR | rs16872526 | 5 | 74675717 | G | T | 0.036 | 0.003 | 4.86E-25 |
| HMGCR | rs74695562 | 5 | 74675951 | G | T | -0.039 | 0.005 | 9.18E-14 |
| HMGCR | rs144083983 | 5 | 74683306 | T | C | -0.048 | 0.004 | 1.02E-29 |
| HMGCR | rs181668591 | 5 | 74689045 | T | C | 0.070 | 0.008 | 1.23E-13 |
| HMGCR | rs114253542 | 5 | 74692810 | C | T | 0.079 | 0.011 | 4.47E-11 |
| HMGCR | rs72633966 | 5 | 74701662 | G | C | 0.064 | 0.003 | 1.72E-113 |
| HMGCR | rs180755046 | 5 | 74703989 | A | C | -0.054 | 0.008 | 2.11E-08 |
| HMGCR | rs182826525 | 5 | 74719210 | G | A | 0.104 | 0.009 | 2.20E-26 |
| HMGCR | rs200823803 | 5 | 74753738 | C | T | 0.060 | 0.007 | 3.41E-14 |
| NPC1L1 | rs3187907 | 7 | 44552209 | C | T | 0.017 | 0.002 | 4.48E-13 |
| NPC1L1 | rs10260606 | 7 | 44584551 | C | G | 0.043 | 0.002 | 1.44E-71 |
| NPC1L1 | rs73107478 | 7 | 44596644 | C | A | 0.040 | 0.003 | 4.98E-28 |
| NPC1L1 | rs217381 | 7 | 44606217 | G | C | -0.031 | 0.002 | 1.07E-58 |
| NPC1L1 | rs79854399 | 7 | 44640315 | T | C | -0.047 | 0.006 | 2.19E-11 |
| NPC1L1 | rs2268310 | 7 | 44677098 | T | C | 0.030 | 0.003 | 3.87E-24 |
| PCSK9 | rs11580527 | 1 | 55411580 | A | G | -0.186 | 0.007 | 4.10E-118 |
| PCSK9 | rs1863668 | 1 | 55411708 | T | G | -0.013 | 0.002 | 2.68E-11 |
| PCSK9 | rs12043403 | 1 | 55431933 | C | T | -0.028 | 0.003 | 2.79E-17 |
| PCSK9 | rs12409233 | 1 | 55436599 | C | G | -0.025 | 0.003 | 5.78E-14 |
| PCSK9 | rs890576 | 1 | 55452897 | G | C | -0.015 | 0.002 | 9.57E-11 |
| PCSK9 | rs146273942 | 1 | 55453841 | A | G | -0.049 | 0.006 | 1.66E-12 |
| PCSK9 | rs145075626 | 1 | 55479043 | G | C | -0.046 | 0.007 | 5.29E-10 |
| PCSK9 | rs2479396 | 1 | 55482091 | G | A | -0.013 | 0.002 | 3.92E-09 |
| PCSK9 | rs146480899 | 1 | 55483477 | G | A | -0.048 | 0.006 | 8.54E-12 |
| PCSK9 | rs77875082 | 1 | 55485042 | A | G | 0.045 | 0.005 | 7.20E-15 |
| PCSK9 | rs2479393 | 1 | 55488369 | A | G | 0.034 | 0.002 | 5.72E-58 |
| PCSK9 | rs34232196 | 1 | 55489542 | T | C | -0.069 | 0.002 | 7.76E-215 |
| PCSK9 | rs374459115 | 1 | 55489968 | A | G | 0.074 | 0.008 | 2.41E-15 |
| PCSK9 | rs28775984 | 1 | 55491161 | C | T | -0.019 | 0.003 | 2.19E-08 |
| PCSK9 | rs181331606 | 1 | 55493672 | G | C | 0.027 | 0.004 | 6.94E-11 |
| PCSK9 | rs187758512 | 1 | 55493674 | G | A | 0.026 | 0.002 | 3.99E-26 |
| PCSK9 | rs72909541 | 1 | 55494301 | T | C | -0.059 | 0.004 | 3.53E-37 |
| PCSK9 | rs12739979 | 1 | 55496648 | T | C | -0.032 | 0.002 | 1.56E-36 |
| PCSK9 | rs11810371 | 1 | 55496861 | A | G | -0.046 | 0.004 | 2.18E-19 |
| PCSK9 | rs2094470 | 1 | 55497302 | C | T | 0.028 | 0.003 | 5.54E-14 |
| PCSK9 | rs72660548 | 1 | 55500978 | G | C | 0.074 | 0.006 | 1.47E-29 |
| PCSK9 | rs2479409 | 1 | 55504650 | G | A | 0.048 | 0.002 | 1.30E-124 |
| PCSK9 | rs11591147 | 1 | 55505647 | T | G | -0.432 | 0.006 | 0.00E+00 |
| PCSK9 | rs11206513 | 1 | 55507649 | C | T | -0.042 | 0.002 | 3.51E-97 |
| PCSK9 | rs74700387 | 1 | 55510015 | T | C | -0.043 | 0.006 | 4.23E-09 |
| PCSK9 | rs7546522 | 1 | 55516713 | T | C | -0.025 | 0.002 | 6.86E-20 |
| PCSK9 | rs572512 | 1 | 55517344 | T | C | 0.026 | 0.002 | 9.97E-34 |
| PCSK9 | rs41294825 | 1 | 55519068 | T | A | -0.042 | 0.004 | 5.98E-20 |
| PCSK9 | rs45508296 | 1 | 55520547 | G | A | -0.069 | 0.006 | 1.88E-22 |
| PCSK9 | rs150119739 | 1 | 55520938 | A | G | 0.067 | 0.004 | 6.48E-41 |
| PCSK9 | rs693668 | 1 | 55521109 | G | A | -0.058 | 0.002 | 1.94E-192 |
| PCSK9 | rs7525503 | 1 | 55522558 | T | G | 0.065 | 0.006 | 8.31E-23 |
| PCSK9 | rs11206517 | 1 | 55526428 | G | T | 0.081 | 0.004 | 2.93E-62 |
| PCSK9 | rs41297885 | 1 | 55526840 | G | C | -0.038 | 0.004 | 1.08E-13 |
| PCSK9 | rs28385715 | 1 | 55532142 | G | T | 0.050 | 0.006 | 3.07E-13 |
| PCSK9 | rs77011887 | 1 | 55534271 | T | C | 0.058 | 0.007 | 1.48E-12 |
| PCSK9 | rs142116310 | 1 | 55536386 | A | G | 0.057 | 0.009 | 1.90E-08 |
| PCSK9 | rs10493176 | 1 | 55538552 | G | T | -0.077 | 0.003 | 7.82E-120 |
| PCSK9 | rs115465289 | 1 | 55580914 | A | G | -0.047 | 0.005 | 1.19E-15 |
| PCSK9 | rs12031153 | 1 | 55601339 | A | G | 0.039 | 0.004 | 1.81E-15 |
| PCSK9 | rs55817205 | 1 | 55609056 | A | G | 0.069 | 0.009 | 1.76E-12 |
| PCSK9 | rs1165227 | 1 | 55616754 | T | C | -0.023 | 0.002 | 1.82E-18 |
| LDLR | rs12983316 | 19 | 11114352 | G | A | 0.043 | 0.002 | 6.32E-61 |
| LDLR | rs13345127 | 19 | 11116266 | T | C | 0.018 | 0.003 | 9.01E-09 |
| LDLR | rs116945003 | 19 | 11119233 | A | G | 0.077 | 0.011 | 1.93E-10 |
| LDLR | rs148724334 | 19 | 11122019 | C | G | 0.055 | 0.007 | 5.59E-11 |
| LDLR | rs73013166 | 19 | 11126160 | C | T | -0.127 | 0.004 | 5.12E-202 |
| LDLR | rs117210556 | 19 | 11126197 | C | T | 0.038 | 0.003 | 1.99E-23 |
| LDLR | rs145033590 | 19 | 11135189 | A | G | 0.067 | 0.010 | 3.08E-09 |
| LDLR | rs3786721 | 19 | 11146499 | T | C | 0.046 | 0.002 | 1.47E-130 |
| LDLR | rs73013176 | 19 | 11147526 | C | T | -0.217 | 0.008 | 1.31E-123 |
| LDLR | rs144834811 | 19 | 11151125 | G | A | 0.042 | 0.004 | 3.55E-17 |
| LDLR | rs144624209 | 19 | 11152888 | A | G | -0.071 | 0.004 | 1.26E-62 |
| LDLR | rs10420325 | 19 | 11158047 | A | T | 0.016 | 0.002 | 8.83E-10 |
| LDLR | rs4804564 | 19 | 11158055 | C | T | -0.047 | 0.004 | 1.46E-27 |
| LDLR | rs36049922 | 19 | 11169305 | C | T | 0.043 | 0.005 | 8.97E-15 |
| LDLR | rs56315738 | 19 | 11175823 | T | C | -0.111 | 0.010 | 1.21E-21 |
| LDLR | rs73015007 | 19 | 11183837 | A | G | -0.073 | 0.002 | 2.06E-240 |
| LDLR | rs10423733 | 19 | 11185919 | C | T | -0.122 | 0.002 | 0.00E+00 |
| LDLR | rs142274292 | 19 | 11191624 | T | C | 0.106 | 0.010 | 5.96E-22 |
| LDLR | rs112159161 | 19 | 11192603 | T | C | -0.179 | 0.007 | 1.76E-106 |
| LDLR | rs146335137 | 19 | 11200514 | T | C | 0.066 | 0.010 | 2.04E-09 |
| LDLR | rs17242353 | 19 | 11200806 | T | C | 0.106 | 0.005 | 3.22E-72 |
| LDLR | rs17242367 | 19 | 11204627 | T | C | 0.023 | 0.003 | 9.49E-09 |
| LDLR | rs17248748 | 19 | 11206040 | T | C | -0.071 | 0.007 | 5.37E-21 |
| LDLR | rs6511721 | 19 | 11206575 | G | A | 0.059 | 0.002 | 1.12E-198 |
| LDLR | rs73015030 | 19 | 11207516 | A | G | -0.134 | 0.005 | 6.43E-127 |
| LDLR | rs28493980 | 19 | 11212179 | G | C | 0.054 | 0.004 | 6.17E-41 |
| LDLR | rs116886614 | 19 | 11221951 | G | A | 0.067 | 0.008 | 1.56E-13 |
| LDLR | rs2738447 | 19 | 11227480 | A | C | -0.056 | 0.002 | 8.61E-190 |
| LDLR | rs41301949 | 19 | 11231335 | G | A | 0.049 | 0.005 | 9.80E-18 |
| LDLR | rs147223423 | 19 | 11232261 | T | A | 0.094 | 0.008 | 1.37E-23 |
| LDLR | rs17249001 | 19 | 11241428 | A | G | 0.073 | 0.004 | 2.98E-68 |
| LDLR | rs3826810 | 19 | 11242133 | A | G | 0.028 | 0.004 | 1.42E-09 |
| LDLR | rs5742911 | 19 | 11243445 | G | A | -0.057 | 0.002 | 1.06E-170 |
| LDLR | rs147540853 | 19 | 11248104 | A | G | -0.082 | 0.005 | 6.63E-46 |
| LDLR | rs138059072 | 19 | 11250617 | T | C | 0.091 | 0.012 | 2.89E-11 |
| LDLR | rs11557092 | 19 | 11257018 | T | C | -0.022 | 0.002 | 1.48E-24 |
| LDLR | rs141618758 | 19 | 11257499 | C | T | 0.058 | 0.006 | 8.50E-17 |
| LDLR | rs62129138 | 19 | 11259528 | A | G | 0.034 | 0.002 | 1.06E-41 |
| LDLR | rs140181075 | 19 | 11264618 | T | C | 0.060 | 0.004 | 6.88E-34 |
| LDLR | rs189143685 | 19 | 11274168 | T | C | 0.056 | 0.008 | 1.38E-09 |
| LDLR | rs4804573 | 19 | 11277232 | A | G | -0.059 | 0.002 | 2.15E-200 |
| LDLR | rs117339792 | 19 | 11282298 | A | G | -0.081 | 0.008 | 1.01E-20 |
| LDLR | rs146576912 | 19 | 11283222 | T | C | -0.145 | 0.004 | 3.23E-198 |
| LDLR | rs4804149 | 19 | 11284028 | C | T | 0.025 | 0.002 | 1.45E-28 |
| LDLR | rs379309 | 19 | 11284302 | C | T | 0.027 | 0.002 | 1.45E-44 |
| LDLR | rs139198665 | 19 | 11284392 | T | C | 0.069 | 0.006 | 1.58E-25 |
| LDLR | rs34824923 | 19 | 11291592 | A | G | 0.029 | 0.004 | 2.29E-11 |
| LDLR | rs34243815 | 19 | 11324965 | T | C | -0.026 | 0.004 | 1.23E-10 |
| LDLR | rs4804150 | 19 | 11327571 | T | C | -0.019 | 0.002 | 1.52E-24 |
| LDLR | rs66466742 | 19 | 11336444 | T | C | -0.045 | 0.004 | 1.10E-22 |
| LDLR | rs1433091 | 19 | 11336626 | A | G | 0.030 | 0.003 | 3.56E-17 |
| LDLR | rs1560699 | 19 | 11337454 | T | C | 0.058 | 0.009 | 1.90E-08 |

EA, effect allele; HMGCR, 3-hydroxy-3-methylglutaryl-CoA reductase; LDLR, low density lipoprotein receptor; NEA, non-effect allele; NPC1L1, NPC1 like intracellular cholesterol transporter 1; PCSK9, proprotein convertase subtilisin/kexin type 9; SE, standard error; SNP, single nucleotide polymorphism.

**Supplementary Table S4.** Genetic variants included in low-density lipoprotein cholesterol (LDL-C) genetic score.

| **SNP** | **Chr** | **Position** | **EA** | **NEA** | **Beta** | **SE** | **P value** |
| --- | --- | --- | --- | --- | --- | --- | --- |
| rs1123571 | 1 | 2326009 | A | G | -0.011 | 0.002 | 7.21E-09 |
| rs75907879 | 1 | 16170903 | T | C | 0.020 | 0.003 | 1.62E-11 |
| rs10903129 | 1 | 25768937 | A | G | -0.025 | 0.002 | 2.28E-40 |
| rs79778025 | 1 | 26449337 | G | A | -0.016 | 0.002 | 2.47E-09 |
| rs79598313 | 1 | 27284913 | T | C | 0.074 | 0.005 | 1.47E-35 |
| rs8681 | 1 | 45468606 | A | G | -0.013 | 0.002 | 3.50E-09 |
| rs2495496 | 1 | 55496449 | T | A | 0.019 | 0.003 | 8.93E-10 |
| rs11591147 | 1 | 55505647 | T | G | -0.432 | 0.006 | 0.00E+00 |
| rs693668 | 1 | 55521109 | G | A | -0.058 | 0.002 | 1.94E-192 |
| rs11206517 | 1 | 55526428 | G | T | 0.081 | 0.004 | 2.93E-62 |
| rs80098465 | 1 | 56323794 | A | G | -0.043 | 0.005 | 4.32E-16 |
| rs7534572 | 1 | 62999675 | C | G | -0.040 | 0.002 | 5.10E-78 |
| rs7515577 | 1 | 93009438 | C | A | -0.025 | 0.002 | 1.49E-27 |
| rs74896173 | 1 | 109167705 | C | T | -0.061 | 0.010 | 1.96E-08 |
| rs12746961 | 1 | 109800321 | T | C | -0.128 | 0.003 | 0.00E+00 |
| rs604349 | 1 | 109839392 | A | G | -0.101 | 0.003 | 2.07E-222 |
| rs116363925 | 1 | 109926599 | T | G | 0.037 | 0.004 | 4.04E-17 |
| rs116056646 | 1 | 109938536 | G | A | 0.036 | 0.005 | 2.77E-09 |
| rs267733 | 1 | 150958836 | G | A | -0.019 | 0.002 | 4.31E-14 |
| rs4390169 | 1 | 155106054 | A | G | 0.013 | 0.002 | 1.22E-11 |
| rs6682862 | 1 | 177938437 | A | G | -0.014 | 0.002 | 1.03E-08 |
| rs1689801 | 1 | 182165484 | A | G | 0.015 | 0.002 | 8.66E-14 |
| rs2296288 | 1 | 183072590 | T | C | -0.011 | 0.002 | 2.82E-09 |
| rs2642438 | 1 | 220970028 | A | G | -0.025 | 0.002 | 1.81E-35 |
| rs553427 | 1 | 234852760 | C | T | -0.038 | 0.002 | 6.09E-91 |
| rs10910522 | 1 | 235011725 | A | G | -0.011 | 0.002 | 6.48E-09 |
| rs3738622 | 1 | 235110859 | T | G | -0.016 | 0.002 | 4.21E-12 |
| rs3820897 | 2 | 3642361 | T | C | 0.016 | 0.002 | 6.97E-12 |
| rs35633876 | 2 | 20363074 | T | G | -0.011 | 0.002 | 1.74E-08 |
| rs13392272 | 2 | 21217490 | T | C | 0.075 | 0.002 | 0.00E+00 |
| rs72902590 | 2 | 21289068 | A | G | -0.092 | 0.004 | 2.12E-79 |
| rs13396400 | 2 | 21502727 | G | A | 0.031 | 0.002 | 1.32E-56 |
| rs11683091 | 2 | 21942735 | T | C | 0.015 | 0.002 | 4.65E-12 |
| rs2068834 | 2 | 27839539 | C | T | 0.014 | 0.002 | 9.39E-12 |
| rs2374569 | 2 | 43885057 | T | C | -0.011 | 0.002 | 1.47E-08 |
| rs185263492 | 2 | 43996767 | A | T | 0.033 | 0.002 | 4.19E-32 |
| rs4299376 | 2 | 44072576 | G | T | 0.065 | 0.002 | 9.36E-228 |
| rs6709904 | 2 | 44080324 | G | A | -0.045 | 0.003 | 8.79E-52 |
| rs149233695 | 2 | 44839083 | T | G | -0.044 | 0.006 | 1.48E-10 |
| rs360804 | 2 | 62939397 | A | G | -0.022 | 0.002 | 8.38E-28 |
| rs10193634 | 2 | 64933985 | C | A | -0.012 | 0.002 | 2.25E-10 |
| rs2970902 | 2 | 88429183 | C | G | 0.012 | 0.002 | 1.37E-09 |
| rs2465956 | 2 | 109065004 | C | T | 0.019 | 0.002 | 3.90E-16 |
| rs150474434 | 2 | 118845121 | A | G | -0.042 | 0.003 | 4.12E-39 |
| rs17050272 | 2 | 121306440 | A | G | -0.020 | 0.002 | 1.90E-24 |
| rs1375131 | 2 | 135954797 | C | T | 0.021 | 0.002 | 5.03E-18 |
| rs10184376 | 2 | 158442005 | T | C | -0.027 | 0.003 | 1.22E-14 |
| rs10184673 | 2 | 169827796 | G | A | 0.020 | 0.002 | 1.64E-24 |
| rs10932008 | 2 | 204048840 | G | A | -0.025 | 0.002 | 1.31E-22 |
| rs1250259 | 2 | 216300482 | T | A | -0.017 | 0.002 | 3.64E-16 |
| rs887829 | 2 | 234668570 | T | C | -0.016 | 0.002 | 1.20E-15 |
| rs1177809 | 3 | 12466490 | G | A | -0.024 | 0.002 | 3.90E-33 |
| rs6792725 | 3 | 24520283 | A | G | 0.016 | 0.002 | 2.52E-14 |
| rs9837622 | 3 | 32514647 | A | T | -0.035 | 0.003 | 7.70E-22 |
| rs71311871 | 3 | 58420613 | G | A | -0.036 | 0.003 | 4.84E-27 |
| rs55921103 | 3 | 69810294 | G | T | -0.011 | 0.002 | 2.34E-08 |
| rs10936349 | 3 | 100453859 | T | C | 0.011 | 0.002 | 4.24E-08 |
| rs3732359 | 3 | 119536429 | G | A | 0.014 | 0.002 | 1.70E-10 |
| rs12054451 | 3 | 122064369 | G | T | 0.015 | 0.002 | 1.13E-12 |
| rs78946096 | 3 | 132188163 | G | A | -0.041 | 0.004 | 5.82E-21 |
| rs9653945 | 3 | 142660706 | A | G | -0.011 | 0.002 | 5.38E-09 |
| rs1584688 | 3 | 160267858 | C | T | 0.013 | 0.002 | 2.09E-11 |
| rs56118251 | 3 | 171534525 | G | A | 0.015 | 0.002 | 1.16E-09 |
| rs13108218 | 4 | 3443931 | A | G | 0.016 | 0.002 | 8.91E-17 |
| rs34707604 | 4 | 69491456 | C | T | 0.030 | 0.002 | 4.66E-30 |
| rs28497720 | 4 | 100487370 | T | C | -0.018 | 0.002 | 2.29E-17 |
| rs17617028 | 4 | 106064683 | A | G | 0.015 | 0.002 | 4.78E-11 |
| rs793900 | 4 | 185253019 | T | C | 0.011 | 0.002 | 1.22E-08 |
| rs77704739 | 5 | 52080909 | C | T | -0.049 | 0.004 | 1.31E-22 |
| rs3010266 | 5 | 71965007 | A | G | -0.015 | 0.002 | 1.32E-09 |
| rs11957820 | 5 | 74350090 | T | C | 0.025 | 0.003 | 1.51E-11 |
| rs12916 | 5 | 74656539 | C | T | 0.072 | 0.002 | 1.94E-314 |
| rs13189347 | 5 | 122828560 | C | A | -0.017 | 0.002 | 1.55E-18 |
| rs6894249 | 5 | 131797547 | G | A | 0.016 | 0.002 | 1.94E-16 |
| rs13161656 | 5 | 141916867 | C | T | 0.022 | 0.003 | 8.88E-14 |
| rs12657266 | 5 | 156396003 | C | T | -0.033 | 0.002 | 2.50E-64 |
| rs7746081 | 6 | 16126934 | A | G | -0.031 | 0.002 | 4.94E-56 |
| rs80215559 | 6 | 25918225 | C | T | -0.058 | 0.003 | 1.42E-49 |
| rs71536537 | 6 | 32523877 | C | T | -0.026 | 0.004 | 5.28E-10 |
| rs6689 | 6 | 32627700 | G | A | 0.041 | 0.002 | 1.07E-56 |
| rs11754773 | 6 | 34577257 | G | A | -0.027 | 0.003 | 2.33E-18 |
| rs6940814 | 6 | 42924932 | A | G | 0.014 | 0.002 | 3.72E-13 |
| rs17665178 | 6 | 52435243 | G | C | -0.018 | 0.002 | 2.97E-18 |
| rs6458949 | 6 | 53508265 | T | G | 0.013 | 0.002 | 3.99E-10 |
| rs9496567 | 6 | 100602753 | A | G | -0.019 | 0.002 | 2.10E-17 |
| rs240762 | 6 | 100985574 | C | T | 0.013 | 0.002 | 1.23E-11 |
| rs4946713 | 6 | 106374015 | A | C | -0.011 | 0.002 | 1.22E-08 |
| rs1556857 | 6 | 116325559 | C | T | -0.015 | 0.002 | 2.96E-16 |
| rs72971192 | 6 | 127088318 | C | T | -0.013 | 0.002 | 1.80E-08 |
| rs7758845 | 6 | 135428537 | C | A | -0.025 | 0.002 | 8.64E-33 |
| rs75393372 | 6 | 139187883 | C | T | 0.020 | 0.003 | 1.58E-08 |
| rs112170089 | 6 | 160502019 | A | G | 0.055 | 0.008 | 1.86E-10 |
| rs12208357 | 6 | 160543148 | T | C | 0.059 | 0.003 | 2.86E-61 |
| rs146534110 | 6 | 160578069 | T | G | 0.075 | 0.008 | 7.28E-16 |
| rs74907759 | 6 | 160857193 | G | A | -0.059 | 0.009 | 1.05E-08 |
| rs117733303 | 6 | 160922870 | G | A | 0.142 | 0.006 | 1.35E-86 |
| rs10455872 | 6 | 161010118 | G | A | 0.112 | 0.003 | 1.24E-191 |
| rs12055389 | 6 | 161091952 | T | C | -0.028 | 0.003 | 8.15E-13 |
| rs10263252 | 7 | 1049949 | A | G | -0.021 | 0.002 | 1.38E-20 |
| rs55696093 | 7 | 21605973 | G | A | 0.038 | 0.002 | 1.25E-62 |
| rs896311 | 7 | 25934357 | G | A | -0.018 | 0.002 | 5.28E-19 |
| rs56001710 | 7 | 25983400 | A | T | -0.018 | 0.002 | 1.94E-13 |
| rs12533280 | 7 | 36171953 | T | C | 0.016 | 0.002 | 8.20E-12 |
| rs10260606 | 7 | 44584551 | C | G | 0.043 | 0.002 | 1.44E-71 |
| rs42122 | 7 | 72841823 | A | G | 0.015 | 0.002 | 9.42E-09 |
| rs6967728 | 7 | 97915637 | A | G | -0.016 | 0.002 | 1.26E-10 |
| rs564449 | 7 | 100321138 | T | G | 0.027 | 0.003 | 2.51E-20 |
| rs2911971 | 8 | 6607634 | C | G | -0.012 | 0.002 | 3.48E-09 |
| rs9987289 | 8 | 9183358 | A | G | -0.057 | 0.003 | 2.20E-72 |
| rs6601302 | 8 | 9239458 | T | G | 0.015 | 0.002 | 1.90E-12 |
| rs900776 | 8 | 21918089 | C | A | -0.019 | 0.002 | 1.60E-14 |
| rs17526980 | 8 | 28950309 | T | C | 0.030 | 0.005 | 3.75E-08 |
| rs28615248 | 8 | 55451193 | C | T | 0.025 | 0.002 | 1.98E-25 |
| rs9297994 | 8 | 59392324 | G | A | 0.032 | 0.002 | 1.64E-58 |
| rs62509311 | 8 | 74907295 | T | A | -0.015 | 0.002 | 6.67E-13 |
| rs2737252 | 8 | 116663898 | A | G | -0.022 | 0.002 | 1.90E-26 |
| rs28601761 | 8 | 126500031 | G | C | -0.057 | 0.002 | 3.06E-198 |
| rs11787335 | 8 | 145044104 | T | C | 0.024 | 0.002 | 1.32E-33 |
| rs3780181 | 9 | 2640759 | G | A | -0.035 | 0.003 | 3.34E-21 |
| rs28498684 | 9 | 16900695 | A | G | 0.013 | 0.002 | 3.92E-11 |
| rs12551960 | 9 | 19267440 | T | C | 0.033 | 0.003 | 9.38E-21 |
| rs615552 | 9 | 22026077 | C | T | 0.011 | 0.002 | 9.53E-09 |
| rs7864568 | 9 | 78212428 | A | G | -0.016 | 0.002 | 1.97E-13 |
| rs7046887 | 9 | 78736048 | T | C | 0.013 | 0.002 | 1.64E-12 |
| rs9410207 | 9 | 91404799 | C | T | -0.021 | 0.003 | 2.99E-08 |
| rs2297400 | 9 | 107599481 | C | T | 0.016 | 0.003 | 4.90E-08 |
| rs2740488 | 9 | 107661742 | C | A | -0.021 | 0.002 | 1.32E-23 |
| rs6478851 | 9 | 131561110 | A | G | -0.015 | 0.002 | 1.25E-11 |
| rs2519093 | 9 | 136141870 | T | C | 0.073 | 0.002 | 1.90E-217 |
| rs11999532 | 9 | 139371405 | C | G | -0.015 | 0.002 | 1.33E-12 |
| rs7903259 | 10 | 17259642 | G | C | 0.017 | 0.002 | 8.12E-20 |
| rs10761750 | 10 | 65128619 | A | G | 0.015 | 0.002 | 1.56E-15 |
| rs2068888 | 10 | 94839642 | A | G | -0.015 | 0.002 | 2.03E-16 |
| rs61886346 | 10 | 96101364 | T | C | -0.022 | 0.003 | 2.78E-08 |
| rs603424 | 10 | 102075479 | A | G | 0.014 | 0.002 | 2.06E-08 |
| rs2792751 | 10 | 113940329 | T | C | 0.023 | 0.002 | 3.40E-30 |
| rs60847460 | 10 | 113983758 | T | C | -0.018 | 0.002 | 9.75E-12 |
| rs4751995 | 10 | 118397884 | A | G | -0.016 | 0.002 | 9.54E-18 |
| rs9423289 | 10 | 124704695 | C | T | -0.022 | 0.002 | 1.66E-31 |
| rs12271225 | 11 | 5690544 | T | A | 0.016 | 0.002 | 4.36E-09 |
| rs11601507 | 11 | 5701074 | A | C | 0.040 | 0.003 | 3.37E-29 |
| rs10832956 | 11 | 18645668 | T | C | -0.021 | 0.002 | 1.75E-24 |
| rs174546 | 11 | 61569830 | T | C | -0.044 | 0.002 | 2.16E-111 |
| rs642803 | 11 | 65560620 | T | C | 0.012 | 0.002 | 7.88E-11 |
| rs2072560 | 11 | 116661826 | T | C | 0.044 | 0.003 | 5.75E-32 |
| rs45505501 | 11 | 118405305 | A | G | 0.018 | 0.002 | 2.25E-11 |
| rs76970536 | 11 | 126250680 | A | G | 0.059 | 0.003 | 1.77E-61 |
| rs34019521 | 12 | 618790 | C | G | 0.019 | 0.002 | 4.29E-17 |
| rs12320328 | 12 | 25408464 | G | A | -0.026 | 0.003 | 3.17E-14 |
| rs11175540 | 12 | 40586295 | A | T | 0.026 | 0.003 | 1.59E-11 |
| rs2251024 | 12 | 51103633 | T | C | -0.017 | 0.002 | 1.10E-17 |
| rs9795910 | 12 | 51795623 | G | A | -0.012 | 0.002 | 4.28E-10 |
| rs11105294 | 12 | 89858839 | A | G | 0.012 | 0.002 | 6.31E-10 |
| rs7955221 | 12 | 100850750 | C | A | -0.012 | 0.002 | 2.00E-09 |
| rs3184504 | 12 | 111884608 | T | C | -0.025 | 0.002 | 1.23E-41 |
| rs1169288 | 12 | 121416650 | C | A | 0.035 | 0.002 | 6.26E-71 |
| rs11057841 | 12 | 125316743 | T | C | 0.021 | 0.002 | 2.55E-14 |
| rs77502095 | 12 | 133119022 | A | G | 0.016 | 0.002 | 1.26E-08 |
| rs7327867 | 13 | 32968591 | G | A | 0.019 | 0.002 | 8.66E-25 |
| rs207637 | 13 | 33085469 | G | T | 0.011 | 0.002 | 2.87E-08 |
| rs9805560 | 13 | 50076079 | G | A | 0.011 | 0.002 | 5.64E-09 |
| rs9524538 | 13 | 95218192 | A | G | 0.017 | 0.002 | 1.45E-15 |
| rs551473284 | 13 | 111038325 | T | C | -0.015 | 0.002 | 1.83E-10 |
| rs6602909 | 13 | 114551993 | C | T | 0.016 | 0.002 | 2.20E-15 |
| rs12016920 | 13 | 114639025 | C | T | -0.017 | 0.002 | 4.97E-12 |
| rs11621792 | 14 | 24871926 | T | C | 0.021 | 0.002 | 2.21E-27 |
| rs11846704 | 14 | 35186694 | T | C | -0.012 | 0.002 | 3.93E-09 |
| rs11620731 | 14 | 70817141 | T | C | -0.025 | 0.002 | 4.15E-21 |
| rs13379043 | 14 | 74250126 | C | T | -0.016 | 0.002 | 1.18E-14 |
| rs17776811 | 14 | 90066451 | A | C | 0.011 | 0.002 | 2.37E-08 |
| rs28929474 | 14 | 94844947 | T | C | 0.065 | 0.006 | 3.45E-20 |
| rs17580 | 14 | 94847262 | A | T | 0.054 | 0.005 | 7.31E-26 |
| rs79391862 | 15 | 53739426 | C | A | -0.065 | 0.006 | 3.95E-21 |
| rs11635502 | 15 | 63771730 | A | G | 0.013 | 0.002 | 4.76E-10 |
| rs11638576 | 15 | 75260387 | A | G | 0.015 | 0.002 | 2.73E-13 |
| rs8041391 | 15 | 89102448 | T | C | 0.015 | 0.002 | 4.66E-08 |
| rs12445804 | 16 | 11706100 | A | G | 0.029 | 0.003 | 6.13E-16 |
| rs7184567 | 16 | 31021078 | T | C | 0.011 | 0.002 | 8.27E-09 |
| rs247617 | 16 | 56990716 | A | C | -0.035 | 0.002 | 3.16E-68 |
| rs7206039 | 16 | 71645914 | A | C | 0.012 | 0.002 | 1.30E-09 |
| rs12924886 | 16 | 72075593 | T | A | 0.054 | 0.002 | 3.96E-117 |
| rs7202323 | 16 | 72217113 | G | T | -0.021 | 0.002 | 9.11E-21 |
| rs67890964 | 16 | 83979317 | C | T | -0.019 | 0.002 | 3.27E-21 |
| rs55714927 | 17 | 7080316 | T | C | -0.035 | 0.002 | 2.11E-45 |
| rs150688657 | 17 | 7505801 | A | G | 0.021 | 0.003 | 9.94E-12 |
| rs704 | 17 | 26694861 | A | G | 0.020 | 0.002 | 6.89E-28 |
| rs67777803 | 17 | 27323322 | T | G | -0.015 | 0.002 | 5.40E-09 |
| rs6505220 | 17 | 29271319 | T | A | -0.014 | 0.002 | 1.13E-10 |
| rs72836561 | 17 | 41926126 | T | C | -0.037 | 0.005 | 1.03E-11 |
| rs11870935 | 17 | 45732605 | G | A | 0.025 | 0.002 | 1.76E-40 |
| rs2665404 | 17 | 57875396 | C | T | -0.011 | 0.002 | 8.44E-09 |
| rs1801689 | 17 | 64210580 | C | A | 0.095 | 0.005 | 1.83E-58 |
| rs17647249 | 17 | 65195226 | T | C | 0.013 | 0.002 | 2.68E-12 |
| rs77542162 | 17 | 67081278 | G | A | 0.169 | 0.006 | 3.34E-125 |
| rs72631343 | 17 | 67191270 | G | C | -0.041 | 0.002 | 1.32E-50 |
| rs2125345 | 17 | 73782191 | C | T | -0.018 | 0.002 | 1.31E-18 |
| rs12451056 | 17 | 76396188 | T | C | -0.020 | 0.002 | 8.72E-15 |
| rs77960347 | 18 | 47109955 | G | A | 0.064 | 0.007 | 2.63E-14 |
| rs4939883 | 18 | 47167214 | T | C | -0.016 | 0.002 | 2.97E-11 |
| rs141453190 | 19 | 10453122 | T | C | -0.033 | 0.005 | 2.40E-08 |
| rs76127343 | 19 | 11065457 | T | C | 0.038 | 0.005 | 1.58E-12 |
| rs10423733 | 19 | 11185919 | C | T | -0.122 | 0.002 | 0.00E+00 |
| rs35878749 | 19 | 11229765 | A | G | -0.043 | 0.002 | 3.17E-106 |
| rs7250652 | 19 | 11302606 | G | A | 0.025 | 0.002 | 1.21E-39 |
| rs58542926 | 19 | 19379549 | T | C | -0.099 | 0.003 | 3.59E-174 |
| rs7937 | 19 | 41302706 | C | T | -0.011 | 0.002 | 6.79E-09 |
| rs73048351 | 19 | 45160086 | A | C | -0.260 | 0.009 | 1.39E-144 |
| rs146842409 | 19 | 45193373 | A | G | 0.123 | 0.015 | 2.67E-13 |
| rs11673631 | 19 | 45225423 | C | G | 0.052 | 0.004 | 7.06E-25 |
| rs1551891 | 19 | 45231821 | A | G | -0.164 | 0.003 | 0.00E+00 |
| rs10406338 | 19 | 45326217 | C | T | 0.083 | 0.002 | 0.00E+00 |
| rs375972689 | 19 | 45360488 | G | T | 0.182 | 0.008 | 3.40E-89 |
| rs10420434 | 19 | 45451190 | A | G | -0.040 | 0.004 | 3.32E-17 |
| rs7247937 | 19 | 45827863 | C | G | -0.016 | 0.002 | 1.13E-14 |
| rs35313547 | 19 | 58352806 | C | T | -0.015 | 0.002 | 7.82E-09 |
| rs34503352 | 19 | 58651296 | A | G | -0.029 | 0.002 | 3.32E-29 |
| rs73066228 | 19 | 59013196 | G | A | 0.017 | 0.002 | 8.45E-12 |
| rs6107650 | 20 | 5526113 | G | A | -0.011 | 0.002 | 2.00E-08 |
| rs438568 | 20 | 12958687 | A | G | -0.013 | 0.002 | 3.35E-12 |
| rs969075 | 20 | 17792323 | T | C | -0.015 | 0.002 | 4.07E-14 |
| rs2618566 | 20 | 17844684 | G | T | 0.040 | 0.002 | 1.55E-86 |
| rs61016611 | 20 | 34212151 | A | G | -0.032 | 0.003 | 1.06E-25 |
| rs1883711 | 20 | 39179822 | C | G | 0.128 | 0.005 | 4.34E-113 |
| rs6093446 | 20 | 39780932 | A | G | 0.024 | 0.002 | 3.96E-30 |
| rs1800961 | 20 | 43042364 | T | C | -0.049 | 0.004 | 5.76E-22 |
| rs2295027 | 20 | 47582884 | A | G | 0.012 | 0.002 | 6.78E-09 |
| rs35046559 | 20 | 62344480 | G | A | 0.012 | 0.002 | 2.85E-08 |
| rs8121509 | 20 | 62712053 | C | T | -0.014 | 0.002 | 4.16E-12 |
| rs60417583 | 20 | 62890877 | T | G | 0.015 | 0.002 | 4.78E-08 |
| rs16988435 | 21 | 33058571 | T | C | 0.032 | 0.004 | 8.42E-14 |
| rs452377 | 21 | 40537183 | C | T | -0.012 | 0.002 | 1.64E-09 |
| rs5746498 | 22 | 18435794 | C | T | 0.014 | 0.002 | 1.42E-09 |
| rs5752963 | 22 | 30203833 | A | G | 0.029 | 0.004 | 3.40E-09 |
| rs4465 | 22 | 35708790 | C | T | 0.014 | 0.002 | 1.78E-13 |
| rs138335 | 22 | 41227086 | C | G | 0.013 | 0.002 | 3.04E-10 |
| rs3747207 | 22 | 44324855 | A | G | -0.014 | 0.002 | 3.33E-10 |
| rs13057311 | 22 | 50878196 | A | G | -0.012 | 0.002 | 2.23E-08 |

EA, effect allele; NEA, non-effect allele; SE, standard error; SNP, single nucleotide polymorphism.

**Supplementary Table S5.** Detailed information about genetic instruments used to proxy body mass index (BMI), systolic blood pressure (SBP), and lifestyle factors.

| **Modifiable factor** | **IVs** | **Unit** | **Participants** | **PubMed ID** |
| --- | --- | --- | --- | --- |
| Body mass index (BMI) | 62 | SD | 339,224 individuals of multi-ancestries | 25673413 |
| Systolic blood pressure (SBP) | 455 | 10 mmHg | Up to 1,006,863 European-descent individuals | 30224653 |
| Smoking initiation | 248 | SD in prevalence of smoking initiation | 1 ,232,091 European-descent individuals | 30643251 |
| Alcohol drinking | 98 | SD increase of log-transformed alcoholic drinks/week | 941,280 European-descent individuals | 30643251 |
| Moderate-to-vigorous intensity physical activity | 16 | More than 30 min per week | 606,820 individuals of multi-ancestries | 36071172 |

IVs, instrumental variables; SD, standard deviation.

**Supplementary Table S6.** Genetic instruments used to calculate genetic scores for body mass index (BMI), systolic blood pressure (SBP), and lifestyle factors.

| **Modifiable** factor | **SNP** | **Chr** | **Position** | **EA** | **NEA** | **Beta** | **SE** | **P value** |
| --- | --- | --- | --- | --- | --- | --- | --- | --- |
| BMI | rs657452 | 1 | 49589847 | A | G | 0.023 | 0.003 | 5.48E-13 |
| BMI | rs3101336 | 1 | 72751185 | C | T | 0.033 | 0.003 | 2.66E-26 |
| BMI | rs12401738 | 1 | 78446761 | A | G | 0.021 | 0.003 | 1.15E-10 |
| BMI | rs11165643 | 1 | 96924097 | T | C | 0.022 | 0.003 | 2.07E-12 |
| BMI | rs17024393 | 1 | 110154688 | C | T | 0.066 | 0.009 | 7.03E-14 |
| BMI | rs543874 | 1 | 177889480 | G | A | 0.048 | 0.004 | 2.62E-35 |
| BMI | rs2820292 | 1 | 201784287 | C | A | 0.020 | 0.003 | 1.83E-10 |
| BMI | rs13021737 | 2 | 632348 | G | A | 0.060 | 0.004 | 1.11E-50 |
| BMI | rs10182181 | 2 | 25150296 | G | A | 0.031 | 0.003 | 8.78E-24 |
| BMI | rs1016287 | 2 | 59305625 | T | C | 0.023 | 0.003 | 2.25E-11 |
| BMI | rs2121279 | 2 | 143043285 | T | C | 0.025 | 0.004 | 2.31E-08 |
| BMI | rs1528435 | 2 | 181550962 | T | C | 0.018 | 0.003 | 1.20E-08 |
| BMI | rs7599312 | 2 | 213413231 | G | A | 0.022 | 0.003 | 1.17E-10 |
| BMI | rs6804842 | 3 | 25106437 | G | A | 0.019 | 0.003 | 2.48E-09 |
| BMI | rs2365389 | 3 | 61236462 | C | T | 0.020 | 0.003 | 1.63E-10 |
| BMI | rs3849570 | 3 | 81792112 | A | C | 0.019 | 0.003 | 2.60E-08 |
| BMI | rs13078960 | 3 | 85807590 | G | T | 0.030 | 0.004 | 1.74E-14 |
| BMI | rs16851483 | 3 | 141275436 | T | G | 0.048 | 0.008 | 3.55E-10 |
| BMI | rs1516725 | 3 | 185824004 | C | T | 0.045 | 0.005 | 1.89E-22 |
| BMI | rs10938397 | 4 | 45182527 | G | A | 0.040 | 0.003 | 3.21E-38 |
| BMI | rs17001654 | 4 | 77129568 | G | C | 0.031 | 0.005 | 7.76E-09 |
| BMI | rs13107325 | 4 | 103188709 | T | C | 0.048 | 0.007 | 1.83E-12 |
| BMI | rs11727676 | 4 | 145659064 | T | C | 0.036 | 0.006 | 2.55E-08 |
| BMI | rs2112347 | 5 | 75015242 | T | G | 0.026 | 0.003 | 6.19E-17 |
| BMI | rs205262 | 6 | 34563164 | G | A | 0.022 | 0.004 | 1.75E-10 |
| BMI | rs2207139 | 6 | 50845490 | G | A | 0.045 | 0.004 | 4.13E-29 |
| BMI | rs9400239 | 6 | 108977663 | C | T | 0.019 | 0.003 | 1.61E-08 |
| BMI | rs13191362 | 6 | 163033350 | A | G | 0.028 | 0.005 | 7.34E-09 |
| BMI | rs1167827 | 7 | 75163169 | G | A | 0.020 | 0.003 | 6.33E-10 |
| BMI | rs2245368 | 7 | 76608143 | C | T | 0.032 | 0.006 | 3.19E-08 |
| BMI | rs17405819 | 8 | 76806584 | T | C | 0.022 | 0.003 | 2.07E-11 |
| BMI | rs2033732 | 8 | 85079709 | C | T | 0.019 | 0.004 | 4.89E-08 |
| BMI | rs4740619 | 9 | 15634326 | T | C | 0.018 | 0.003 | 4.56E-09 |
| BMI | rs10968576 | 9 | 28414339 | G | A | 0.025 | 0.003 | 6.61E-14 |
| BMI | rs6477694 | 9 | 111932342 | C | T | 0.017 | 0.003 | 2.67E-08 |
| BMI | rs1928295 | 9 | 120378483 | T | C | 0.019 | 0.003 | 7.91E-10 |
| BMI | rs10733682 | 9 | 129460914 | A | G | 0.017 | 0.003 | 1.83E-08 |
| BMI | rs7899106 | 10 | 87410904 | G | A | 0.040 | 0.007 | 2.96E-08 |
| BMI | rs17094222 | 10 | 102395440 | C | T | 0.025 | 0.004 | 5.94E-11 |
| BMI | rs7903146 | 10 | 114758349 | C | T | 0.023 | 0.003 | 1.11E-11 |
| BMI | rs4256980 | 11 | 8673939 | G | C | 0.021 | 0.003 | 2.90E-11 |
| BMI | rs11030104 | 11 | 27684517 | A | G | 0.041 | 0.004 | 5.56E-28 |
| BMI | rs2176598 | 11 | 43864278 | T | C | 0.020 | 0.004 | 2.97E-08 |
| BMI | rs3817334 | 11 | 47650993 | T | C | 0.026 | 0.003 | 5.15E-17 |
| BMI | rs12286929 | 11 | 115022404 | G | A | 0.022 | 0.003 | 1.31E-12 |
| BMI | rs7138803 | 12 | 50247468 | A | G | 0.032 | 0.003 | 8.15E-24 |
| BMI | rs11057405 | 12 | 122781897 | G | A | 0.031 | 0.006 | 2.02E-08 |
| BMI | rs12429545 | 13 | 54102206 | A | G | 0.033 | 0.005 | 1.09E-12 |
| BMI | rs10132280 | 14 | 25928179 | C | A | 0.023 | 0.003 | 1.14E-11 |
| BMI | rs7141420 | 14 | 79899454 | T | C | 0.024 | 0.003 | 1.23E-14 |
| BMI | rs3736485 | 15 | 51748610 | A | G | 0.018 | 0.003 | 7.41E-09 |
| BMI | rs16951275 | 15 | 68077168 | T | C | 0.031 | 0.004 | 1.91E-17 |
| BMI | rs758747 | 16 | 3627358 | T | C | 0.023 | 0.004 | 7.47E-10 |
| BMI | rs3888190 | 16 | 28889486 | A | C | 0.031 | 0.003 | 3.14E-23 |
| BMI | rs1558902 | 16 | 53803574 | A | T | 0.082 | 0.003 | 7.51E-153 |
| BMI | rs1000940 | 17 | 5283252 | G | A | 0.019 | 0.003 | 1.28E-08 |
| BMI | rs12940622 | 17 | 78615571 | G | A | 0.018 | 0.003 | 2.49E-09 |
| BMI | rs1808579 | 18 | 21104888 | C | T | 0.017 | 0.003 | 4.17E-08 |
| BMI | rs6567160 | 18 | 57829135 | C | T | 0.056 | 0.004 | 3.93E-53 |
| BMI | rs17724992 | 19 | 18454825 | A | G | 0.019 | 0.004 | 3.42E-08 |
| BMI | rs29941 | 19 | 34309532 | G | A | 0.018 | 0.003 | 2.41E-08 |
| BMI | rs2287019 | 19 | 46202172 | C | T | 0.036 | 0.004 | 4.59E-18 |
| SBP | rs7796 | 1 | 1684169 | C | G | 0.339 | 0.031 | 5.00E-27 |
| SBP | rs263532 | 1 | 2164116 | T | C | 0.180 | 0.031 | 4.72E-09 |
| SBP | rs2493296 | 1 | 3327032 | T | C | 0.418 | 0.044 | 3.14E-21 |
| SBP | rs2232460 | 1 | 6659505 | A | G | -0.217 | 0.032 | 1.10E-11 |
| SBP | rs488834 | 1 | 10767902 | T | C | -0.380 | 0.037 | 2.35E-25 |
| SBP | rs6699618 | 1 | 11881441 | C | G | 0.912 | 0.041 | 1.68E-109 |
| SBP | rs75461554 | 1 | 15810172 | T | C | -0.302 | 0.038 | 1.18E-15 |
| SBP | rs1889785 | 1 | 16348729 | A | G | 0.178 | 0.030 | 4.35E-09 |
| SBP | rs404100 | 1 | 25366987 | T | C | 0.194 | 0.030 | 1.68E-10 |
| SBP | rs34079867 | 1 | 27407850 | T | C | 0.199 | 0.035 | 1.78E-08 |
| SBP | rs4908348 | 1 | 28706949 | T | G | 0.237 | 0.033 | 8.07E-13 |
| SBP | rs11210029 | 1 | 41865293 | A | G | -0.203 | 0.031 | 8.92E-11 |
| SBP | rs1408945 | 1 | 42364877 | T | G | -0.320 | 0.030 | 8.33E-26 |
| SBP | rs1209384 | 1 | 43765089 | A | G | 0.256 | 0.031 | 2.85E-16 |
| SBP | rs778124 | 1 | 56606206 | A | G | 0.297 | 0.031 | 1.45E-21 |
| SBP | rs61772592 | 1 | 56979681 | A | G | -0.318 | 0.046 | 2.86E-12 |
| SBP | rs12063372 | 1 | 59621911 | A | G | 0.199 | 0.032 | 3.86E-10 |
| SBP | rs12136922 | 1 | 67007389 | A | G | 0.203 | 0.030 | 2.69E-11 |
| SBP | rs658780 | 1 | 78555928 | T | G | -0.203 | 0.035 | 5.29E-09 |
| SBP | rs786923 | 1 | 89242954 | T | C | -0.308 | 0.031 | 2.83E-23 |
| SBP | rs7514579 | 1 | 94051350 | A | C | 0.224 | 0.036 | 5.45E-10 |
| SBP | rs10776752 | 1 | 113044328 | T | G | 0.821 | 0.058 | 4.61E-46 |
| SBP | rs59980837 | 1 | 115827266 | T | G | 1.100 | 0.116 | 3.32E-21 |
| SBP | rs11585169 | 1 | 150572037 | A | T | 0.180 | 0.031 | 5.34E-09 |
| SBP | rs76719272 | 1 | 156129796 | T | C | -0.274 | 0.046 | 2.97E-09 |
| SBP | rs12731646 | 1 | 169090660 | T | C | -0.189 | 0.031 | 7.21E-10 |
| SBP | rs1043069 | 1 | 180859368 | T | G | 0.234 | 0.031 | 5.26E-14 |
| SBP | rs4651224 | 1 | 184585182 | T | C | 0.199 | 0.031 | 9.00E-11 |
| SBP | rs12042924 | 1 | 197297417 | T | C | -0.181 | 0.030 | 2.62E-09 |
| SBP | rs11120093 | 1 | 207211326 | T | C | -0.179 | 0.031 | 5.13E-09 |
| SBP | rs2724377 | 1 | 207974818 | A | G | 0.194 | 0.030 | 1.29E-10 |
| SBP | rs7555285 | 1 | 209970355 | C | G | 0.229 | 0.038 | 1.05E-09 |
| SBP | rs68085857 | 1 | 217737629 | T | C | 0.274 | 0.036 | 1.68E-14 |
| SBP | rs4595370 | 1 | 221298122 | A | G | -0.209 | 0.033 | 1.73E-10 |
| SBP | rs1745417 | 1 | 228204279 | T | C | 0.287 | 0.030 | 1.59E-21 |
| SBP | rs699 | 1 | 230845794 | A | G | -0.375 | 0.031 | 5.59E-34 |
| SBP | rs1565440 | 1 | 243387788 | A | G | 0.175 | 0.031 | 1.94E-08 |
| SBP | rs4926499 | 1 | 249155909 | C | G | 0.297 | 0.044 | 1.33E-11 |
| SBP | rs17760259 | 2 | 19744462 | T | C | -0.265 | 0.030 | 2.25E-18 |
| SBP | rs2384063 | 2 | 25187115 | T | C | 0.327 | 0.036 | 6.33E-20 |
| SBP | rs1275988 | 2 | 26914364 | T | C | -0.541 | 0.031 | 4.42E-69 |
| SBP | rs13420463 | 2 | 37517566 | A | G | 0.314 | 0.036 | 2.72E-18 |
| SBP | rs4952609 | 2 | 40555733 | A | G | 0.212 | 0.035 | 9.60E-10 |
| SBP | rs115262049 | 2 | 43196694 | A | T | 0.589 | 0.055 | 1.29E-26 |
| SBP | rs12464602 | 2 | 43397614 | A | G | -0.244 | 0.032 | 1.02E-14 |
| SBP | rs13016772 | 2 | 55779476 | T | C | 0.252 | 0.036 | 1.23E-12 |
| SBP | rs2249105 | 2 | 65287896 | A | G | 0.293 | 0.031 | 7.63E-21 |
| SBP | rs10188003 | 2 | 66773469 | T | C | 0.188 | 0.031 | 8.80E-10 |
| SBP | rs6731373 | 2 | 68503044 | A | G | 0.191 | 0.033 | 4.18E-09 |
| SBP | rs6732123 | 2 | 69534650 | C | G | -0.174 | 0.031 | 1.52E-08 |
| SBP | rs4577304 | 2 | 73403040 | T | C | -0.177 | 0.030 | 4.99E-09 |
| SBP | rs72847885 | 2 | 86326717 | A | G | 0.241 | 0.032 | 3.08E-14 |
| SBP | rs10207726 | 2 | 112744260 | T | C | -0.214 | 0.033 | 8.06E-11 |
| SBP | rs6737318 | 2 | 114083120 | A | G | 0.235 | 0.036 | 1.13E-10 |
| SBP | rs2580350 | 2 | 121996007 | A | G | 0.177 | 0.031 | 8.39E-09 |
| SBP | rs17257081 | 2 | 135630498 | A | G | 0.227 | 0.039 | 6.35E-09 |
| SBP | rs55944332 | 2 | 145726621 | A | G | -0.261 | 0.036 | 1.79E-13 |
| SBP | rs62170470 | 2 | 146989797 | T | C | 0.197 | 0.032 | 7.69E-10 |
| SBP | rs62187653 | 2 | 162469128 | T | C | 0.329 | 0.051 | 1.23E-10 |
| SBP | rs4667454 | 2 | 164867726 | A | G | 0.264 | 0.032 | 2.63E-16 |
| SBP | rs73029563 | 2 | 165008166 | C | G | -0.514 | 0.030 | 4.20E-64 |
| SBP | rs10048760 | 2 | 174977976 | T | G | -0.186 | 0.030 | 6.56E-10 |
| SBP | rs71421551 | 2 | 177053298 | C | G | 0.237 | 0.033 | 1.05E-12 |
| SBP | rs17610485 | 2 | 177540601 | A | T | 0.174 | 0.030 | 1.06E-08 |
| SBP | rs4894132 | 2 | 180738654 | T | C | 0.247 | 0.034 | 5.51E-13 |
| SBP | rs12473915 | 2 | 182987704 | A | G | -0.295 | 0.038 | 3.42E-15 |
| SBP | rs13412750 | 2 | 191634958 | A | G | -0.289 | 0.034 | 2.33E-17 |
| SBP | rs12693982 | 2 | 204085635 | T | C | 0.258 | 0.031 | 7.49E-17 |
| SBP | rs3845811 | 2 | 208521512 | C | G | -0.294 | 0.031 | 1.88E-21 |
| SBP | rs12694277 | 2 | 213188795 | T | C | -0.202 | 0.034 | 1.80E-09 |
| SBP | rs2161967 | 2 | 218680529 | T | G | 0.284 | 0.031 | 2.87E-20 |
| SBP | rs3828282 | 2 | 218779144 | C | G | 0.186 | 0.032 | 5.29E-09 |
| SBP | rs10804330 | 2 | 227185749 | T | C | 0.235 | 0.031 | 1.62E-14 |
| SBP | rs1044822 | 2 | 230629138 | T | C | -0.248 | 0.042 | 5.16E-09 |
| SBP | rs3754944 | 2 | 231279616 | A | C | 0.177 | 0.031 | 9.30E-09 |
| SBP | rs139354822 | 2 | 242344695 | T | C | 0.612 | 0.098 | 3.51E-10 |
| SBP | rs9848170 | 3 | 11495983 | C | G | 0.323 | 0.031 | 7.01E-26 |
| SBP | rs11925504 | 3 | 14943965 | A | G | -0.290 | 0.031 | 1.78E-21 |
| SBP | rs189267552 | 3 | 20073193 | A | T | -0.866 | 0.139 | 4.55E-10 |
| SBP | rs2643826 | 3 | 27562988 | T | C | 0.447 | 0.031 | 1.74E-48 |
| SBP | rs68115553 | 3 | 27704702 | A | G | -0.645 | 0.114 | 1.74E-08 |
| SBP | rs743395 | 3 | 37598382 | T | C | 0.260 | 0.032 | 2.55E-16 |
| SBP | rs6788984 | 3 | 41107173 | A | G | 0.300 | 0.043 | 3.81E-12 |
| SBP | rs1052501 | 3 | 41925398 | T | C | 0.226 | 0.041 | 4.14E-08 |
| SBP | rs6771917 | 3 | 48108442 | T | C | -0.379 | 0.036 | 1.39E-26 |
| SBP | rs7615099 | 3 | 53143901 | A | G | 0.189 | 0.032 | 3.90E-09 |
| SBP | rs6445583 | 3 | 53562894 | A | G | 0.277 | 0.035 | 1.90E-15 |
| SBP | rs3772219 | 3 | 56771251 | A | C | 0.273 | 0.032 | 3.10E-17 |
| SBP | rs7618284 | 3 | 66422246 | C | G | -0.189 | 0.033 | 1.10E-08 |
| SBP | rs4499560 | 3 | 70920485 | A | T | -0.220 | 0.033 | 1.46E-11 |
| SBP | rs1375564 | 3 | 85656311 | T | C | 0.258 | 0.032 | 2.84E-16 |
| SBP | rs12637573 | 3 | 121682388 | A | G | -0.173 | 0.030 | 9.95E-09 |
| SBP | rs6438857 | 3 | 124557643 | T | C | 0.274 | 0.031 | 3.13E-19 |
| SBP | rs9880098 | 3 | 133949366 | A | G | 0.308 | 0.031 | 1.59E-23 |
| SBP | rs1199330 | 3 | 138101529 | A | G | -0.265 | 0.047 | 1.65E-08 |
| SBP | rs9876694 | 3 | 141152017 | T | C | 0.471 | 0.065 | 4.64E-13 |
| SBP | rs4408839 | 3 | 153729768 | A | G | -0.230 | 0.035 | 2.43E-11 |
| SBP | rs79539362 | 3 | 154680449 | T | C | 0.400 | 0.050 | 2.09E-15 |
| SBP | rs17684859 | 3 | 158213841 | T | C | -0.224 | 0.034 | 4.24E-11 |
| SBP | rs3980686 | 3 | 168697602 | T | G | -0.500 | 0.049 | 1.03E-24 |
| SBP | rs1290784 | 3 | 169096900 | T | C | 0.412 | 0.030 | 2.97E-42 |
| SBP | rs2111557 | 3 | 169325621 | T | C | 0.176 | 0.030 | 5.22E-09 |
| SBP | rs4955575 | 3 | 169534538 | A | C | 0.216 | 0.035 | 5.63E-10 |
| SBP | rs262986 | 3 | 183435713 | A | G | -0.237 | 0.031 | 7.67E-15 |
| SBP | rs13091418 | 3 | 185329756 | C | G | -0.223 | 0.033 | 6.15E-12 |
| SBP | rs9869437 | 3 | 196228360 | A | C | -0.200 | 0.032 | 3.22E-10 |
| SBP | rs34535756 | 4 | 2246927 | T | C | 0.478 | 0.079 | 1.18E-09 |
| SBP | rs1290933 | 4 | 2668217 | A | C | -0.285 | 0.033 | 3.17E-18 |
| SBP | rs2498323 | 4 | 3451109 | A | G | 0.317 | 0.052 | 8.52E-10 |
| SBP | rs2610990 | 4 | 18008232 | A | G | -0.290 | 0.034 | 2.86E-17 |
| SBP | rs55924432 | 4 | 26812737 | T | C | 0.265 | 0.032 | 5.70E-17 |
| SBP | rs2291434 | 4 | 38387244 | T | G | -0.262 | 0.030 | 5.10E-18 |
| SBP | rs12511987 | 4 | 46595623 | T | G | -0.233 | 0.040 | 5.39E-09 |
| SBP | rs62309747 | 4 | 48713862 | A | G | -0.224 | 0.030 | 1.59E-13 |
| SBP | rs60991988 | 4 | 54801228 | T | G | 0.379 | 0.050 | 2.82E-14 |
| SBP | rs13107261 | 4 | 63768826 | A | G | -0.178 | 0.031 | 1.57E-08 |
| SBP | rs5020545 | 4 | 77414988 | T | C | -0.218 | 0.031 | 9.71E-13 |
| SBP | rs12509595 | 4 | 81182554 | T | C | -0.837 | 0.033 | 2.55E-138 |
| SBP | rs6823199 | 4 | 83925895 | T | C | 0.209 | 0.035 | 1.72E-09 |
| SBP | rs17010957 | 4 | 86719165 | T | C | -0.534 | 0.043 | 1.78E-35 |
| SBP | rs13149209 | 4 | 89750668 | T | C | 0.281 | 0.037 | 1.97E-14 |
| SBP | rs13107325 | 4 | 103188709 | T | C | -0.909 | 0.059 | 4.22E-53 |
| SBP | rs11097909 | 4 | 106911321 | T | C | -0.363 | 0.043 | 3.35E-17 |
| SBP | rs1493132 | 4 | 108861082 | T | C | -0.177 | 0.032 | 2.73E-08 |
| SBP | rs1814951 | 4 | 111408718 | A | G | -0.323 | 0.047 | 3.91E-12 |
| SBP | rs4834792 | 4 | 120555696 | A | T | 0.197 | 0.030 | 7.24E-11 |
| SBP | rs7439567 | 4 | 138464842 | T | C | 0.254 | 0.031 | 2.31E-16 |
| SBP | rs72719160 | 4 | 144051276 | A | T | -0.224 | 0.032 | 4.34E-12 |
| SBP | rs2353940 | 4 | 145740898 | T | C | -0.208 | 0.036 | 6.85E-09 |
| SBP | rs73855810 | 4 | 148383424 | A | G | 0.273 | 0.043 | 3.04E-10 |
| SBP | rs7683728 | 4 | 156402654 | T | C | -0.365 | 0.030 | 2.43E-33 |
| SBP | rs12643599 | 4 | 156639846 | A | G | 0.313 | 0.031 | 1.23E-23 |
| SBP | rs17035181 | 4 | 157678511 | T | G | 0.307 | 0.043 | 7.61E-13 |
| SBP | rs869396 | 4 | 169688000 | A | C | -0.212 | 0.031 | 4.12E-12 |
| SBP | rs4957026 | 5 | 361148 | A | G | 0.198 | 0.032 | 8.12E-10 |
| SBP | rs10069690 | 5 | 1279790 | T | C | 0.310 | 0.037 | 4.47E-17 |
| SBP | rs7725413 | 5 | 15695987 | T | C | -0.199 | 0.036 | 3.07E-08 |
| SBP | rs12656497 | 5 | 32831939 | T | C | -0.638 | 0.031 | 7.14E-96 |
| SBP | rs10941043 | 5 | 33194751 | T | G | -0.259 | 0.033 | 6.42E-15 |
| SBP | rs2113077 | 5 | 50799442 | A | G | 0.210 | 0.031 | 6.09E-12 |
| SBP | rs1694068 | 5 | 53283630 | A | T | 0.266 | 0.031 | 1.18E-17 |
| SBP | rs10043077 | 5 | 55692939 | T | C | -0.193 | 0.032 | 2.52E-09 |
| SBP | rs34496659 | 5 | 61798934 | A | G | 0.455 | 0.062 | 1.54E-13 |
| SBP | rs6870654 | 5 | 63831964 | T | C | 0.214 | 0.035 | 7.58E-10 |
| SBP | rs4286632 | 5 | 66291370 | A | G | 0.211 | 0.034 | 7.64E-10 |
| SBP | rs7703560 | 5 | 67678506 | A | G | -0.225 | 0.033 | 1.51E-11 |
| SBP | rs246973 | 5 | 68007803 | T | C | 0.248 | 0.034 | 1.45E-13 |
| SBP | rs6452769 | 5 | 87389027 | A | G | -0.314 | 0.038 | 7.82E-17 |
| SBP | rs76443575 | 5 | 96211594 | C | G | -0.523 | 0.082 | 1.40E-10 |
| SBP | rs1871190 | 5 | 97953719 | T | G | 0.195 | 0.032 | 1.66E-09 |
| SBP | rs11241313 | 5 | 114428167 | T | C | -0.207 | 0.033 | 2.23E-10 |
| SBP | rs1624823 | 5 | 122475438 | A | G | 0.337 | 0.031 | 4.26E-27 |
| SBP | rs9327297 | 5 | 122835051 | C | G | 0.275 | 0.032 | 8.07E-18 |
| SBP | rs758179 | 5 | 127354424 | C | G | -0.209 | 0.037 | 1.19E-08 |
| SBP | rs6892983 | 5 | 127845030 | A | C | 0.343 | 0.031 | 7.11E-29 |
| SBP | rs702395 | 5 | 140086677 | T | C | 0.232 | 0.031 | 3.24E-14 |
| SBP | rs2913920 | 5 | 141726983 | T | C | 0.242 | 0.036 | 1.62E-11 |
| SBP | rs1957563 | 5 | 157474590 | T | C | 0.363 | 0.034 | 2.32E-26 |
| SBP | rs11960210 | 5 | 157817634 | T | C | 0.473 | 0.031 | 1.25E-51 |
| SBP | rs13358657 | 5 | 157938070 | A | G | -0.388 | 0.045 | 2.95E-18 |
| SBP | rs3860770 | 5 | 173301427 | A | G | -0.266 | 0.033 | 1.20E-15 |
| SBP | rs12153395 | 5 | 179411477 | A | G | -0.330 | 0.049 | 1.07E-11 |
| SBP | rs2745599 | 6 | 1613686 | A | G | 0.216 | 0.032 | 8.96E-12 |
| SBP | rs1575290 | 6 | 7715689 | T | C | 0.197 | 0.030 | 5.59E-11 |
| SBP | rs1630736 | 6 | 12295987 | T | C | -0.171 | 0.031 | 3.52E-08 |
| SBP | rs9349379 | 6 | 12903957 | A | G | 0.266 | 0.031 | 1.31E-17 |
| SBP | rs9368222 | 6 | 20686996 | A | C | 0.228 | 0.034 | 1.84E-11 |
| SBP | rs2655445 | 6 | 22377623 | A | G | -0.202 | 0.031 | 9.58E-11 |
| SBP | rs79782817 | 6 | 25882678 | T | G | 0.532 | 0.050 | 1.43E-26 |
| SBP | rs116025100 | 6 | 30935290 | A | G | 0.537 | 0.085 | 2.86E-10 |
| SBP | rs2753960 | 6 | 31762844 | T | G | 0.447 | 0.031 | 2.66E-47 |
| SBP | rs2815063 | 6 | 39262535 | A | C | 0.276 | 0.046 | 1.76E-09 |
| SBP | rs7763558 | 6 | 43349215 | A | G | 0.336 | 0.032 | 1.17E-25 |
| SBP | rs11967262 | 6 | 43760327 | C | G | -0.172 | 0.031 | 3.43E-08 |
| SBP | rs78648104 | 6 | 50683009 | T | C | -0.429 | 0.054 | 2.37E-15 |
| SBP | rs1984195 | 6 | 79657391 | A | G | 0.241 | 0.030 | 1.77E-15 |
| SBP | rs9361836 | 6 | 82235408 | T | C | 0.220 | 0.032 | 1.25E-11 |
| SBP | rs6921291 | 6 | 97066242 | T | C | 0.358 | 0.039 | 1.58E-20 |
| SBP | rs9486916 | 6 | 109013930 | T | C | 0.266 | 0.039 | 5.42E-12 |
| SBP | rs961764 | 6 | 117522156 | C | G | -0.191 | 0.031 | 3.75E-10 |
| SBP | rs10782230 | 6 | 126228512 | A | G | 0.211 | 0.030 | 2.91E-12 |
| SBP | rs9401913 | 6 | 127159982 | A | G | 0.520 | 0.031 | 3.66E-65 |
| SBP | rs2327429 | 6 | 134209837 | T | C | 0.200 | 0.034 | 3.16E-09 |
| SBP | rs8180684 | 6 | 143200936 | T | C | 0.213 | 0.034 | 1.80E-10 |
| SBP | rs7765526 | 6 | 147713764 | A | G | 0.201 | 0.031 | 5.88E-11 |
| SBP | rs17080102 | 6 | 151004770 | C | G | -0.809 | 0.059 | 3.52E-42 |
| SBP | rs1293969 | 6 | 151959945 | T | C | -0.199 | 0.035 | 1.03E-08 |
| SBP | rs509833 | 6 | 159711515 | A | G | 0.329 | 0.044 | 7.08E-14 |
| SBP | rs12661036 | 6 | 163737476 | T | C | -0.210 | 0.037 | 1.82E-08 |
| SBP | rs7744902 | 6 | 166176722 | A | G | -0.409 | 0.059 | 5.64E-12 |
| SBP | rs6959688 | 7 | 1966831 | A | G | -0.234 | 0.031 | 4.22E-14 |
| SBP | rs10282122 | 7 | 2529623 | T | C | -0.302 | 0.033 | 2.46E-20 |
| SBP | rs73049928 | 7 | 4669949 | A | G | -0.238 | 0.039 | 1.20E-09 |
| SBP | rs3807925 | 7 | 18543250 | A | G | -0.186 | 0.032 | 5.39E-09 |
| SBP | rs28688791 | 7 | 19039605 | T | C | -0.322 | 0.038 | 2.34E-17 |
| SBP | rs112509803 | 7 | 24735004 | C | G | -0.264 | 0.048 | 3.18E-08 |
| SBP | rs3735533 | 7 | 27245893 | T | C | -0.910 | 0.058 | 5.29E-56 |
| SBP | rs6961048 | 7 | 27328187 | C | G | -0.530 | 0.050 | 1.43E-26 |
| SBP | rs977184 | 7 | 28650761 | T | C | -0.184 | 0.031 | 4.86E-09 |
| SBP | rs11977526 | 7 | 46008110 | A | G | -0.321 | 0.031 | 6.62E-25 |
| SBP | rs12668436 | 7 | 47548893 | T | C | -0.215 | 0.035 | 7.88E-10 |
| SBP | rs848445 | 7 | 77572461 | T | C | -0.203 | 0.034 | 2.28E-09 |
| SBP | rs42377 | 7 | 92243672 | A | G | -0.315 | 0.033 | 1.69E-21 |
| SBP | rs2392929 | 7 | 106414069 | T | G | -0.751 | 0.038 | 1.96E-87 |
| SBP | rs34072724 | 7 | 130432469 | A | G | -0.242 | 0.030 | 1.37E-15 |
| SBP | rs35680304 | 7 | 130973495 | T | C | 0.269 | 0.031 | 3.76E-18 |
| SBP | rs75672964 | 7 | 131321010 | T | C | 0.589 | 0.084 | 2.35E-12 |
| SBP | rs73727605 | 7 | 149474622 | A | G | 0.362 | 0.062 | 6.60E-09 |
| SBP | rs3918226 | 7 | 150690176 | T | C | 0.664 | 0.058 | 8.46E-31 |
| SBP | rs10224210 | 7 | 151413194 | T | C | -0.383 | 0.034 | 1.60E-29 |
| SBP | rs1870735 | 7 | 155744303 | C | G | 0.206 | 0.031 | 3.61E-11 |
| SBP | rs71499040 | 8 | 1711918 | C | G | 0.222 | 0.034 | 5.63E-11 |
| SBP | rs1821002 | 8 | 10640065 | C | G | 0.379 | 0.031 | 5.19E-35 |
| SBP | rs10866828 | 8 | 23401534 | T | C | 0.248 | 0.036 | 3.19E-12 |
| SBP | rs7821832 | 8 | 25889446 | T | G | 0.422 | 0.035 | 6.67E-34 |
| SBP | rs77375686 | 8 | 26043622 | A | G | -0.347 | 0.049 | 8.38E-13 |
| SBP | rs1906672 | 8 | 38130025 | A | G | 0.297 | 0.036 | 1.20E-16 |
| SBP | rs4873492 | 8 | 51947549 | T | C | 0.343 | 0.040 | 1.61E-17 |
| SBP | rs2354862 | 8 | 64501744 | A | C | 0.251 | 0.032 | 2.42E-15 |
| SBP | rs13253358 | 8 | 68920135 | T | C | 0.213 | 0.033 | 1.13E-10 |
| SBP | rs2126474 | 8 | 76878957 | T | G | -0.260 | 0.031 | 1.87E-17 |
| SBP | rs9918879 | 8 | 77681093 | T | G | -0.298 | 0.050 | 2.28E-09 |
| SBP | rs148401029 | 8 | 81386066 | A | C | -0.462 | 0.085 | 4.97E-08 |
| SBP | rs10091532 | 8 | 82853793 | A | C | -0.207 | 0.031 | 1.33E-11 |
| SBP | rs843093 | 8 | 92528310 | A | G | -0.209 | 0.034 | 6.95E-10 |
| SBP | rs2613203 | 8 | 95253197 | A | T | -0.268 | 0.039 | 5.81E-12 |
| SBP | rs79069610 | 8 | 105921209 | T | C | -0.401 | 0.073 | 3.68E-08 |
| SBP | rs35783704 | 8 | 105966258 | A | G | -0.462 | 0.051 | 8.81E-20 |
| SBP | rs7830607 | 8 | 110097287 | A | G | -0.206 | 0.033 | 3.09E-10 |
| SBP | rs2470004 | 8 | 120358445 | T | C | -0.345 | 0.039 | 1.28E-18 |
| SBP | rs4598218 | 8 | 129483956 | T | C | 0.191 | 0.031 | 1.00E-09 |
| SBP | rs7012866 | 8 | 135616959 | T | G | -0.233 | 0.030 | 1.21E-14 |
| SBP | rs4440615 | 8 | 141057641 | A | G | -0.220 | 0.031 | 1.87E-12 |
| SBP | rs4961293 | 8 | 141812374 | T | C | 0.227 | 0.030 | 7.35E-14 |
| SBP | rs7463212 | 8 | 143991858 | A | T | -0.275 | 0.031 | 1.81E-19 |
| SBP | rs60191654 | 9 | 753648 | A | G | -0.238 | 0.039 | 5.88E-10 |
| SBP | rs927315 | 9 | 4117713 | T | C | 0.169 | 0.030 | 2.44E-08 |
| SBP | rs1332813 | 9 | 9350706 | T | C | 0.220 | 0.031 | 2.32E-12 |
| SBP | rs9886665 | 9 | 22942770 | T | C | 0.205 | 0.034 | 2.47E-09 |
| SBP | rs4553000 | 9 | 34223553 | T | C | -0.204 | 0.030 | 1.09E-11 |
| SBP | rs76452347 | 9 | 35906471 | T | C | -0.297 | 0.040 | 7.13E-14 |
| SBP | rs10746963 | 9 | 77238558 | A | G | -0.218 | 0.039 | 2.05E-08 |
| SBP | rs7045409 | 9 | 95201540 | A | T | -0.186 | 0.031 | 2.55E-09 |
| SBP | rs10980408 | 9 | 113249071 | T | C | -0.761 | 0.083 | 3.83E-20 |
| SBP | rs2900568 | 9 | 116689758 | T | C | -0.189 | 0.030 | 2.96E-10 |
| SBP | rs34025993 | 9 | 123516572 | A | G | 0.223 | 0.031 | 4.71E-13 |
| SBP | rs7854147 | 9 | 125863350 | A | G | 0.306 | 0.046 | 3.29E-11 |
| SBP | rs13289468 | 9 | 128180332 | A | C | 0.249 | 0.031 | 3.93E-16 |
| SBP | rs6271 | 9 | 136522274 | T | C | -0.555 | 0.061 | 1.18E-19 |
| SBP | rs11145807 | 9 | 139520789 | A | G | 0.214 | 0.032 | 3.54E-11 |
| SBP | rs11252324 | 10 | 4124568 | T | G | -0.416 | 0.057 | 3.61E-13 |
| SBP | rs1623474 | 10 | 18471794 | T | C | 0.383 | 0.032 | 7.66E-33 |
| SBP | rs12258967 | 10 | 18727959 | C | G | 0.633 | 0.034 | 1.08E-78 |
| SBP | rs3802517 | 10 | 28233469 | A | T | 0.253 | 0.030 | 4.65E-17 |
| SBP | rs12264186 | 10 | 32289986 | T | C | 0.214 | 0.039 | 3.58E-08 |
| SBP | rs4948643 | 10 | 45379759 | T | C | 0.226 | 0.034 | 2.40E-11 |
| SBP | rs34130368 | 10 | 48411796 | T | G | -0.302 | 0.050 | 1.28E-09 |
| SBP | rs4245599 | 10 | 60365755 | A | G | -0.179 | 0.031 | 4.04E-09 |
| SBP | rs57946343 | 10 | 63499951 | T | C | 0.716 | 0.043 | 2.10E-63 |
| SBP | rs2236295 | 10 | 64564892 | T | G | -0.303 | 0.031 | 1.05E-22 |
| SBP | rs2177843 | 10 | 75409877 | T | C | 0.439 | 0.043 | 2.80E-24 |
| SBP | rs10749572 | 10 | 82136664 | T | G | -0.203 | 0.030 | 1.88E-11 |
| SBP | rs111866816 | 10 | 94441507 | T | C | 0.357 | 0.060 | 2.29E-09 |
| SBP | rs2689690 | 10 | 95899706 | T | C | -0.270 | 0.032 | 1.15E-17 |
| SBP | rs2274224 | 10 | 96039597 | C | G | -0.452 | 0.030 | 5.99E-50 |
| SBP | rs1006545 | 10 | 102553647 | T | G | 0.685 | 0.048 | 3.50E-46 |
| SBP | rs11191580 | 10 | 104906211 | T | C | 1.100 | 0.055 | 7.74E-89 |
| SBP | rs117464403 | 10 | 107158054 | A | G | 0.864 | 0.120 | 5.80E-13 |
| SBP | rs12255372 | 10 | 114808902 | T | G | 0.236 | 0.034 | 1.94E-12 |
| SBP | rs1801253 | 10 | 115805056 | C | G | 0.463 | 0.034 | 2.84E-41 |
| SBP | rs72842207 | 10 | 121433675 | T | C | -0.203 | 0.037 | 3.14E-08 |
| SBP | rs11592107 | 10 | 122968964 | A | G | 0.302 | 0.033 | 1.55E-20 |
| SBP | rs7093894 | 10 | 124234880 | A | C | 0.236 | 0.043 | 3.16E-08 |
| SBP | rs7912283 | 10 | 133773019 | A | G | -0.214 | 0.032 | 2.94E-11 |
| SBP | rs1133400 | 10 | 134459388 | A | G | -0.298 | 0.038 | 2.53E-15 |
| SBP | rs569550 | 11 | 1887068 | T | G | -0.577 | 0.032 | 1.33E-73 |
| SBP | rs74048190 | 11 | 2114221 | T | C | -0.440 | 0.076 | 6.07E-09 |
| SBP | rs360153 | 11 | 9762274 | T | C | -0.345 | 0.031 | 1.73E-29 |
| SBP | rs2014408 | 11 | 16365282 | T | C | 0.517 | 0.037 | 1.26E-43 |
| SBP | rs7926335 | 11 | 16917869 | T | C | 0.314 | 0.034 | 2.52E-20 |
| SBP | rs17762 | 11 | 22492454 | A | G | 0.412 | 0.057 | 5.60E-13 |
| SBP | rs1382472 | 11 | 27273967 | A | G | -0.192 | 0.031 | 4.47E-10 |
| SBP | rs871004 | 11 | 28512458 | A | G | 0.234 | 0.032 | 1.65E-13 |
| SBP | rs10501122 | 11 | 30192151 | T | C | 0.192 | 0.032 | 1.18E-09 |
| SBP | rs11604310 | 11 | 45351420 | T | C | -0.278 | 0.041 | 1.46E-11 |
| SBP | rs7107356 | 11 | 47676170 | A | G | -0.460 | 0.030 | 1.63E-52 |
| SBP | rs2904315 | 11 | 48109948 | A | G | -0.208 | 0.033 | 1.58E-10 |
| SBP | rs4427587 | 11 | 58436648 | T | C | 0.206 | 0.031 | 4.28E-11 |
| SBP | rs7125196 | 11 | 61272565 | T | C | 0.442 | 0.047 | 7.31E-21 |
| SBP | rs2306363 | 11 | 65405600 | T | G | -0.436 | 0.038 | 5.24E-31 |
| SBP | rs7395791 | 11 | 69262916 | A | G | -0.216 | 0.031 | 2.19E-12 |
| SBP | rs10501410 | 11 | 72088806 | A | G | 0.412 | 0.061 | 1.10E-11 |
| SBP | rs7927515 | 11 | 76125330 | A | C | 0.227 | 0.032 | 1.05E-12 |
| SBP | rs2289124 | 11 | 89224477 | A | G | -0.308 | 0.042 | 1.14E-13 |
| SBP | rs604723 | 11 | 100610546 | T | C | -0.655 | 0.034 | 2.55E-83 |
| SBP | rs629864 | 11 | 100699139 | T | C | -0.187 | 0.032 | 4.69E-09 |
| SBP | rs7926110 | 11 | 107086143 | T | G | 0.260 | 0.032 | 5.71E-16 |
| SBP | rs236916 | 11 | 117089628 | A | G | 0.317 | 0.045 | 1.31E-12 |
| SBP | rs573455 | 11 | 117267884 | A | G | 0.199 | 0.030 | 4.77E-11 |
| SBP | rs11222084 | 11 | 130273230 | A | T | -0.336 | 0.032 | 1.80E-26 |
| SBP | rs7944927 | 11 | 130490917 | T | C | 0.224 | 0.039 | 1.23E-08 |
| SBP | rs78998485 | 12 | 434755 | C | G | -0.245 | 0.035 | 1.48E-12 |
| SBP | rs3819532 | 12 | 2436837 | T | C | -0.188 | 0.031 | 9.44E-10 |
| SBP | rs2024385 | 12 | 12888438 | A | T | -0.264 | 0.031 | 5.88E-18 |
| SBP | rs1010064 | 12 | 20000315 | A | C | 0.357 | 0.039 | 3.02E-20 |
| SBP | rs73075659 | 12 | 20373541 | A | G | 0.396 | 0.032 | 5.52E-35 |
| SBP | rs2129869 | 12 | 26457650 | A | T | -0.264 | 0.036 | 2.44E-13 |
| SBP | rs9651825 | 12 | 27159784 | A | G | -0.204 | 0.034 | 1.93E-09 |
| SBP | rs61917655 | 12 | 48210787 | T | C | 0.343 | 0.051 | 2.68E-11 |
| SBP | rs12426261 | 12 | 50573037 | A | G | 0.378 | 0.031 | 2.31E-34 |
| SBP | rs7134440 | 12 | 53450097 | T | C | 0.479 | 0.056 | 1.58E-17 |
| SBP | rs7134677 | 12 | 54441498 | T | C | -0.385 | 0.033 | 4.46E-31 |
| SBP | rs7306710 | 12 | 66376091 | T | C | -0.243 | 0.030 | 1.03E-15 |
| SBP | rs4143175 | 12 | 67782397 | T | C | 0.219 | 0.035 | 5.10E-10 |
| SBP | rs7963801 | 12 | 79685226 | T | C | -0.236 | 0.031 | 2.87E-14 |
| SBP | rs6539467 | 12 | 79955306 | A | G | 0.265 | 0.040 | 5.57E-11 |
| SBP | rs17249754 | 12 | 90060586 | A | G | -0.845 | 0.040 | 1.25E-97 |
| SBP | rs10777213 | 12 | 90349999 | A | G | -0.179 | 0.030 | 2.45E-09 |
| SBP | rs5742643 | 12 | 102837863 | T | C | -0.223 | 0.035 | 1.53E-10 |
| SBP | rs7310615 | 12 | 111865049 | C | G | 0.585 | 0.031 | 1.32E-81 |
| SBP | rs1896326 | 12 | 115342956 | A | G | -0.280 | 0.037 | 4.41E-14 |
| SBP | rs35444 | 12 | 115552437 | A | G | 0.437 | 0.031 | 3.47E-45 |
| SBP | rs6490019 | 12 | 115920472 | A | G | -0.290 | 0.031 | 6.61E-21 |
| SBP | rs1169078 | 12 | 122416254 | C | G | -0.197 | 0.033 | 1.68E-09 |
| SBP | rs117206641 | 12 | 133086888 | T | C | 0.315 | 0.050 | 2.66E-10 |
| SBP | rs483071 | 13 | 22294117 | T | C | 0.271 | 0.031 | 5.09E-18 |
| SBP | rs9507885 | 13 | 27951090 | T | C | -0.321 | 0.054 | 3.23E-09 |
| SBP | rs9508495 | 13 | 30146201 | T | C | -0.356 | 0.035 | 6.34E-24 |
| SBP | rs2065498 | 13 | 41893105 | T | G | -0.293 | 0.040 | 3.36E-13 |
| SBP | rs7491248 | 13 | 47180671 | A | G | 0.216 | 0.036 | 2.38E-09 |
| SBP | rs9526707 | 13 | 51489186 | A | G | -0.204 | 0.032 | 2.77E-10 |
| SBP | rs75961402 | 13 | 56398286 | A | G | 0.266 | 0.042 | 1.95E-10 |
| SBP | rs17245822 | 13 | 73131694 | A | C | -0.190 | 0.031 | 1.15E-09 |
| SBP | rs78474310 | 13 | 73826901 | A | G | -0.470 | 0.073 | 1.51E-10 |
| SBP | rs6562778 | 13 | 74223828 | A | G | 0.178 | 0.030 | 4.96E-09 |
| SBP | rs9549627 | 13 | 113652369 | A | G | 0.285 | 0.050 | 1.25E-08 |
| SBP | rs7331680 | 13 | 115000650 | T | G | 0.410 | 0.042 | 3.35E-22 |
| SBP | rs365990 | 14 | 23861811 | A | G | 0.225 | 0.031 | 5.95E-13 |
| SBP | rs8904 | 14 | 35871217 | A | G | 0.306 | 0.031 | 1.71E-22 |
| SBP | rs7493678 | 14 | 39400917 | A | T | -0.189 | 0.032 | 2.31E-09 |
| SBP | rs72683923 | 14 | 50735947 | T | C | 0.959 | 0.110 | 3.08E-18 |
| SBP | rs35413927 | 14 | 53420358 | A | G | -0.300 | 0.033 | 5.25E-20 |
| SBP | rs57140819 | 14 | 68018247 | C | G | 0.242 | 0.040 | 1.30E-09 |
| SBP | rs11847049 | 14 | 69259406 | C | G | -0.227 | 0.036 | 4.44E-10 |
| SBP | rs11159091 | 14 | 75074316 | A | G | 0.198 | 0.030 | 6.79E-11 |
| SBP | rs7154723 | 14 | 98590629 | A | G | 0.253 | 0.031 | 2.72E-16 |
| SBP | rs17562391 | 14 | 100133250 | T | C | 0.197 | 0.031 | 1.35E-10 |
| SBP | rs75016974 | 14 | 100197940 | T | C | -0.251 | 0.044 | 1.05E-08 |
| SBP | rs12885878 | 14 | 104007555 | A | G | -0.229 | 0.037 | 4.32E-10 |
| SBP | rs8030856 | 15 | 40314967 | C | G | -0.176 | 0.031 | 1.21E-08 |
| SBP | rs28866311 | 15 | 41442195 | T | G | -0.276 | 0.030 | 5.45E-20 |
| SBP | rs4775769 | 15 | 48939888 | T | G | -0.416 | 0.052 | 7.76E-16 |
| SBP | rs3098186 | 15 | 50810621 | T | C | -0.242 | 0.030 | 1.41E-15 |
| SBP | rs2652812 | 15 | 63406170 | T | C | -0.252 | 0.035 | 1.03E-12 |
| SBP | rs28429256 | 15 | 66931617 | A | G | 0.215 | 0.033 | 3.89E-11 |
| SBP | rs11636952 | 15 | 75114322 | T | C | 0.531 | 0.033 | 4.22E-59 |
| SBP | rs2627313 | 15 | 81006712 | T | C | 0.321 | 0.030 | 3.55E-26 |
| SBP | rs2046341 | 15 | 86040872 | A | G | -0.254 | 0.038 | 2.74E-11 |
| SBP | rs77032376 | 15 | 90010780 | T | C | -0.273 | 0.043 | 2.35E-10 |
| SBP | rs4932373 | 15 | 91429287 | A | C | -0.635 | 0.033 | 2.49E-83 |
| SBP | rs12906962 | 15 | 95312071 | T | C | -0.265 | 0.033 | 3.28E-16 |
| SBP | rs2589218 | 15 | 96785017 | T | C | -0.226 | 0.034 | 2.54E-11 |
| SBP | rs4606697 | 15 | 100087596 | A | G | -0.320 | 0.052 | 9.71E-10 |
| SBP | rs11641374 | 16 | 1347717 | A | C | -0.194 | 0.031 | 3.26E-10 |
| SBP | rs12596630 | 16 | 2065666 | T | C | 0.428 | 0.055 | 5.01E-15 |
| SBP | rs72778133 | 16 | 3578718 | T | C | -0.242 | 0.044 | 4.98E-08 |
| SBP | rs111929315 | 16 | 4136871 | A | G | 0.315 | 0.049 | 8.60E-11 |
| SBP | rs12446456 | 16 | 4922201 | T | C | -0.300 | 0.030 | 2.97E-23 |
| SBP | rs77924615 | 16 | 20392332 | A | G | -0.408 | 0.039 | 1.12E-25 |
| SBP | rs7186298 | 16 | 21088031 | T | C | -0.232 | 0.030 | 1.88E-14 |
| SBP | rs8044992 | 16 | 24811207 | T | C | 0.214 | 0.033 | 1.07E-10 |
| SBP | rs34941092 | 16 | 50550137 | A | G | -0.323 | 0.043 | 3.23E-14 |
| SBP | rs4784541 | 16 | 51704452 | T | C | -0.202 | 0.031 | 4.93E-11 |
| SBP | rs2060664 | 16 | 60652439 | T | C | 0.216 | 0.035 | 4.06E-10 |
| SBP | rs146550789 | 16 | 66781040 | T | C | -0.482 | 0.078 | 5.64E-10 |
| SBP | rs62047964 | 16 | 70729954 | T | C | 0.512 | 0.069 | 9.29E-14 |
| SBP | rs1012089 | 16 | 74171973 | C | G | -0.192 | 0.030 | 1.95E-10 |
| SBP | rs4888408 | 16 | 75432824 | A | G | 0.365 | 0.031 | 1.42E-32 |
| SBP | rs12926550 | 16 | 81510155 | A | G | -0.255 | 0.032 | 3.43E-15 |
| SBP | rs3950627 | 16 | 86436343 | A | C | 0.185 | 0.031 | 1.82E-09 |
| SBP | rs6540119 | 16 | 87984477 | A | T | 0.202 | 0.032 | 3.93E-10 |
| SBP | rs908951 | 16 | 89697625 | T | C | -0.226 | 0.032 | 7.14E-13 |
| SBP | rs9303175 | 17 | 1372987 | T | G | -0.205 | 0.033 | 3.65E-10 |
| SBP | rs11653927 | 17 | 2012094 | T | C | -0.280 | 0.031 | 1.17E-19 |
| SBP | rs113086489 | 17 | 7171356 | T | C | 0.325 | 0.031 | 3.80E-26 |
| SBP | rs4511593 | 17 | 7455536 | T | C | -0.288 | 0.032 | 1.28E-19 |
| SBP | rs117285318 | 17 | 7870642 | T | C | 0.441 | 0.059 | 6.93E-14 |
| SBP | rs4925159 | 17 | 18185510 | A | G | 0.217 | 0.031 | 9.66E-13 |
| SBP | rs7218708 | 17 | 19926836 | A | G | -0.178 | 0.030 | 4.38E-09 |
| SBP | rs1551355 | 17 | 30032420 | T | C | 0.210 | 0.036 | 3.89E-09 |
| SBP | rs9899540 | 17 | 30777924 | A | T | 0.201 | 0.032 | 1.87E-10 |
| SBP | rs7213273 | 17 | 43155914 | A | G | -0.400 | 0.032 | 6.24E-37 |
| SBP | rs17608766 | 17 | 45013271 | T | C | -0.690 | 0.043 | 2.48E-57 |
| SBP | rs3764400 | 17 | 46123932 | T | C | 0.375 | 0.045 | 3.69E-17 |
| SBP | rs9897429 | 17 | 47518378 | A | G | 0.265 | 0.032 | 1.19E-16 |
| SBP | rs1000423 | 17 | 59475642 | T | C | 0.414 | 0.035 | 6.50E-33 |
| SBP | rs56288724 | 17 | 60767135 | A | G | -0.218 | 0.031 | 2.01E-12 |
| SBP | rs62076622 | 17 | 61090958 | A | G | 0.236 | 0.038 | 3.79E-10 |
| SBP | rs6504213 | 17 | 62381714 | T | C | -0.298 | 0.031 | 1.25E-21 |
| SBP | rs1436138 | 17 | 75316880 | A | G | 0.312 | 0.032 | 4.73E-23 |
| SBP | rs9302885 | 17 | 76799898 | A | G | 0.224 | 0.030 | 1.03E-13 |
| SBP | rs11655604 | 17 | 79365861 | T | C | -0.203 | 0.033 | 1.09E-09 |
| SBP | rs34413141 | 18 | 777282 | A | T | -0.353 | 0.039 | 2.47E-19 |
| SBP | rs62082230 | 18 | 22676071 | A | T | -0.188 | 0.035 | 4.69E-08 |
| SBP | rs1154214 | 18 | 24546824 | T | G | -0.203 | 0.031 | 3.27E-11 |
| SBP | rs56407827 | 18 | 42179819 | T | C | 0.360 | 0.034 | 2.78E-26 |
| SBP | rs11874246 | 18 | 42596789 | T | C | 0.286 | 0.033 | 3.23E-18 |
| SBP | rs7245140 | 18 | 43095231 | T | C | -0.337 | 0.039 | 7.67E-18 |
| SBP | rs1437649 | 18 | 48132646 | A | G | -0.219 | 0.036 | 8.57E-10 |
| SBP | rs665445 | 18 | 51842682 | A | C | -0.191 | 0.033 | 1.15E-08 |
| SBP | rs10048404 | 18 | 54578482 | T | C | -0.261 | 0.032 | 1.91E-16 |
| SBP | rs10460108 | 18 | 73034151 | A | G | 0.214 | 0.030 | 1.12E-12 |
| SBP | rs698748 | 19 | 1424888 | A | G | 0.187 | 0.033 | 8.90E-09 |
| SBP | rs149339216 | 19 | 2144046 | T | C | -0.691 | 0.078 | 6.93E-19 |
| SBP | rs68096471 | 19 | 5175709 | A | G | -0.210 | 0.034 | 9.26E-10 |
| SBP | rs12985940 | 19 | 7262734 | T | C | 0.464 | 0.043 | 1.08E-26 |
| SBP | rs167479 | 19 | 11526765 | T | G | -0.564 | 0.033 | 7.21E-67 |
| SBP | rs1077795 | 19 | 17222584 | A | G | 0.251 | 0.034 | 3.33E-13 |
| SBP | rs62112908 | 19 | 22213956 | A | G | -0.239 | 0.042 | 1.25E-08 |
| SBP | rs28572357 | 19 | 31867447 | A | C | -0.273 | 0.031 | 6.34E-19 |
| SBP | rs1433121 | 19 | 32591878 | T | C | -0.228 | 0.033 | 2.66E-12 |
| SBP | rs33836 | 19 | 34008600 | T | C | 0.177 | 0.030 | 6.56E-09 |
| SBP | rs10420519 | 19 | 45298461 | T | G | -0.492 | 0.089 | 2.86E-08 |
| SBP | rs7255933 | 19 | 45766729 | A | G | 0.231 | 0.035 | 2.44E-11 |
| SBP | rs11672660 | 19 | 46180184 | T | C | 0.221 | 0.038 | 6.32E-09 |
| SBP | rs571689 | 19 | 49207554 | T | C | 0.228 | 0.030 | 6.77E-14 |
| SBP | rs73046792 | 19 | 49605705 | A | G | -0.355 | 0.043 | 7.23E-17 |
| SBP | rs6054139 | 20 | 6327810 | A | G | 0.209 | 0.031 | 8.23E-12 |
| SBP | rs2423514 | 20 | 10693337 | A | G | 0.301 | 0.030 | 1.77E-23 |
| SBP | rs6108787 | 20 | 10967214 | T | G | -0.427 | 0.030 | 5.38E-46 |
| SBP | rs6078093 | 20 | 11168669 | A | G | -0.185 | 0.030 | 1.20E-09 |
| SBP | rs8125763 | 20 | 17883531 | A | C | 0.176 | 0.030 | 4.84E-09 |
| SBP | rs17812022 | 20 | 19007099 | T | C | -0.361 | 0.053 | 5.65E-12 |
| SBP | rs6058088 | 20 | 30139886 | T | G | 0.283 | 0.042 | 1.14E-11 |
| SBP | rs79384779 | 20 | 31214944 | T | C | 0.318 | 0.043 | 1.08E-13 |
| SBP | rs6029756 | 20 | 40266681 | A | G | -0.271 | 0.033 | 1.88E-16 |
| SBP | rs6031431 | 20 | 42795152 | A | G | -0.262 | 0.030 | 7.05E-18 |
| SBP | rs2598 | 20 | 47241618 | A | G | 0.168 | 0.030 | 2.87E-08 |
| SBP | rs6090907 | 20 | 47410231 | A | G | -0.385 | 0.043 | 1.29E-19 |
| SBP | rs234623 | 20 | 57488964 | A | G | -0.180 | 0.030 | 2.43E-09 |
| SBP | rs6026744 | 20 | 57742388 | A | T | -0.713 | 0.046 | 7.00E-54 |
| SBP | rs28374392 | 20 | 61189717 | T | C | 0.192 | 0.034 | 1.21E-08 |
| SBP | rs6062324 | 20 | 62446351 | A | G | -0.329 | 0.036 | 1.18E-19 |
| SBP | rs2776037 | 21 | 16317933 | T | C | -0.185 | 0.031 | 2.15E-09 |
| SBP | rs1882961 | 21 | 16556367 | T | C | 0.244 | 0.033 | 6.69E-14 |
| SBP | rs2833834 | 21 | 33814378 | A | C | 0.218 | 0.034 | 1.22E-10 |
| SBP | rs12627651 | 21 | 44760603 | A | G | 0.350 | 0.034 | 1.02E-24 |
| SBP | rs34487963 | 21 | 44838330 | A | C | -0.882 | 0.124 | 1.35E-12 |
| SBP | rs7278003 | 21 | 44966069 | T | C | -0.188 | 0.030 | 6.63E-10 |
| SBP | rs2238787 | 22 | 19976406 | A | G | 0.255 | 0.033 | 1.45E-14 |
| SBP | rs12321 | 22 | 29453193 | C | G | -0.229 | 0.030 | 3.81E-14 |
| SBP | rs113264678 | 22 | 30135079 | T | C | 0.406 | 0.073 | 2.26E-08 |
| SBP | rs8142376 | 22 | 32001037 | T | C | 0.168 | 0.030 | 2.20E-08 |
| SBP | rs148140538 | 22 | 50228044 | T | C | -0.325 | 0.056 | 7.39E-09 |
| SBP | rs28578714 | 22 | 50727921 | T | C | 0.207 | 0.033 | 2.53E-10 |
| Smoking | rs2708630 | 1 | 8447404 | T | C | -0.010 | 0.002 | 9.99E-10 |
| Smoking | rs56169608 | 1 | 18439991 | A | G | 0.009 | 0.002 | 1.00E-08 |
| Smoking | rs72664906 | 1 | 32170141 | C | T | 0.014 | 0.002 | 1.85E-09 |
| Smoking | rs12742446 | 1 | 33877057 | T | G | 0.012 | 0.002 | 2.74E-10 |
| Smoking | rs951740 | 1 | 44011737 | A | G | 0.017 | 0.002 | 3.78E-26 |
| Smoking | rs10458563 | 1 | 50612250 | G | A | 0.017 | 0.002 | 2.17E-18 |
| Smoking | rs332827 | 1 | 61743160 | A | G | -0.009 | 0.002 | 2.81E-08 |
| Smoking | rs1937443 | 1 | 66469643 | G | C | 0.014 | 0.002 | 1.27E-18 |
| Smoking | rs1084445 | 1 | 72996521 | C | T | -0.012 | 0.002 | 1.80E-10 |
| Smoking | rs11210229 | 1 | 73860028 | G | A | -0.014 | 0.002 | 4.94E-18 |
| Smoking | rs3895907 | 1 | 75006027 | G | A | -0.013 | 0.002 | 1.56E-14 |
| Smoking | rs11162976 | 1 | 80822282 | C | A | 0.011 | 0.002 | 6.47E-10 |
| Smoking | rs11162019 | 1 | 87913176 | T | C | -0.010 | 0.002 | 3.40E-09 |
| Smoking | rs12133063 | 1 | 91214714 | A | C | 0.012 | 0.002 | 1.15E-12 |
| Smoking | rs12036050 | 1 | 96917491 | C | T | 0.012 | 0.002 | 7.18E-11 |
| Smoking | rs10745324 | 1 | 112708722 | G | A | -0.010 | 0.002 | 1.97E-08 |
| Smoking | rs12027999 | 1 | 154206358 | C | T | -0.018 | 0.002 | 1.54E-13 |
| Smoking | rs10753630 | 1 | 163790451 | T | C | 0.011 | 0.002 | 9.96E-11 |
| Smoking | rs12079063 | 1 | 174079585 | G | A | -0.009 | 0.002 | 3.88E-08 |
| Smoking | rs147052174 | 1 | 179783167 | T | G | 0.034 | 0.006 | 1.31E-08 |
| Smoking | rs12760908 | 1 | 190888339 | T | C | 0.011 | 0.002 | 3.90E-08 |
| Smoking | rs55921136 | 1 | 210359333 | C | T | -0.014 | 0.002 | 1.90E-12 |
| Smoking | rs76132272 | 1 | 227456065 | T | C | 0.018 | 0.003 | 9.43E-09 |
| Smoking | rs3905125 | 1 | 236864459 | T | C | 0.010 | 0.002 | 4.28E-11 |
| Smoking | rs4659805 | 1 | 237859755 | T | G | 0.009 | 0.002 | 8.36E-09 |
| Smoking | rs10927039 | 1 | 243721726 | C | T | -0.013 | 0.002 | 2.39E-10 |
| Smoking | rs62107261 | 2 | 422144 | C | T | -0.026 | 0.004 | 2.13E-11 |
| Smoking | rs6728726 | 2 | 623976 | C | T | 0.019 | 0.002 | 4.27E-19 |
| Smoking | rs61533748 | 2 | 22582968 | C | T | 0.010 | 0.002 | 1.50E-10 |
| Smoking | rs62135525 | 2 | 44299879 | T | C | -0.022 | 0.004 | 4.61E-09 |
| Smoking | rs1004787 | 2 | 45159091 | A | G | 0.016 | 0.002 | 3.91E-24 |
| Smoking | rs2678903 | 2 | 58137930 | G | A | 0.010 | 0.002 | 1.25E-10 |
| Smoking | rs143909875 | 2 | 59294197 | C | T | 0.018 | 0.003 | 3.74E-12 |
| Smoking | rs7585579 | 2 | 60024857 | G | C | 0.013 | 0.002 | 1.29E-14 |
| Smoking | rs17432775 | 2 | 63466463 | T | C | -0.011 | 0.002 | 2.55E-08 |
| Smoking | rs12714017 | 2 | 80999398 | C | T | 0.010 | 0.002 | 6.57E-10 |
| Smoking | rs11695197 | 2 | 97711421 | A | G | 0.014 | 0.002 | 6.28E-09 |
| Smoking | rs9679319 | 2 | 104134432 | G | T | 0.018 | 0.002 | 4.52E-23 |
| Smoking | rs6705147 | 2 | 133196926 | T | C | 0.010 | 0.002 | 5.24E-09 |
| Smoking | rs34367058 | 2 | 137513264 | T | C | 0.014 | 0.002 | 1.36E-13 |
| Smoking | rs13030994 | 2 | 146143090 | A | G | 0.021 | 0.002 | 2.42E-39 |
| Smoking | rs1994247 | 2 | 156023165 | T | G | 0.012 | 0.002 | 1.25E-14 |
| Smoking | rs11693702 | 2 | 162802184 | A | T | 0.014 | 0.002 | 9.33E-19 |
| Smoking | rs13009008 | 2 | 174043233 | G | A | -0.010 | 0.002 | 6.92E-09 |
| Smoking | rs4374330 | 2 | 182058432 | T | C | 0.013 | 0.002 | 3.73E-12 |
| Smoking | rs62181771 | 2 | 201042954 | A | G | 0.010 | 0.002 | 4.47E-10 |
| Smoking | rs13398418 | 2 | 203717108 | A | T | 0.012 | 0.002 | 2.09E-11 |
| Smoking | rs2135160 | 2 | 213214467 | C | T | 0.016 | 0.003 | 8.23E-09 |
| Smoking | rs2163413 | 2 | 226349200 | G | A | -0.014 | 0.002 | 3.06E-11 |
| Smoking | rs1485272 | 3 | 3727589 | C | T | -0.009 | 0.002 | 4.07E-08 |
| Smoking | rs4479577 | 3 | 5723818 | T | C | 0.009 | 0.002 | 2.11E-08 |
| Smoking | rs7629352 | 3 | 16848835 | G | A | 0.010 | 0.002 | 7.82E-09 |
| Smoking | rs11716705 | 3 | 34728753 | G | A | 0.012 | 0.002 | 1.40E-10 |
| Smoking | rs12632110 | 3 | 50224225 | G | A | -0.014 | 0.002 | 1.18E-16 |
| Smoking | rs11130381 | 3 | 53850005 | T | C | -0.009 | 0.002 | 4.83E-08 |
| Smoking | rs11926232 | 3 | 55936417 | G | A | 0.021 | 0.004 | 1.16E-08 |
| Smoking | rs11720703 | 3 | 71060640 | T | C | 0.011 | 0.002 | 5.58E-12 |
| Smoking | rs62258903 | 3 | 75102433 | G | C | -0.016 | 0.002 | 7.20E-12 |
| Smoking | rs62254171 | 3 | 84624766 | A | G | 0.031 | 0.005 | 3.73E-09 |
| Smoking | rs62250713 | 3 | 85513793 | G | A | -0.019 | 0.002 | 1.63E-30 |
| Smoking | rs66680800 | 3 | 85985324 | T | G | -0.013 | 0.002 | 1.13E-14 |
| Smoking | rs4857114 | 3 | 94213083 | T | C | 0.009 | 0.002 | 3.03E-08 |
| Smoking | rs326341 | 3 | 107811142 | A | G | -0.010 | 0.002 | 6.16E-10 |
| Smoking | rs6438208 | 3 | 114170272 | A | G | -0.010 | 0.002 | 7.33E-09 |
| Smoking | rs1499982 | 3 | 117820386 | T | C | 0.022 | 0.002 | 3.01E-23 |
| Smoking | rs62266876 | 3 | 128976451 | G | C | 0.015 | 0.003 | 3.06E-08 |
| Smoking | rs34638471 | 3 | 146367076 | A | G | -0.010 | 0.002 | 2.49E-08 |
| Smoking | rs114050142 | 3 | 150805177 | A | T | -0.023 | 0.003 | 2.56E-11 |
| Smoking | rs963354 | 3 | 157393770 | A | C | 0.011 | 0.002 | 2.35E-10 |
| Smoking | rs12485391 | 3 | 181205593 | A | C | 0.014 | 0.002 | 1.89E-08 |
| Smoking | rs12642744 | 4 | 28027176 | T | G | -0.012 | 0.002 | 5.98E-11 |
| Smoking | rs58400863 | 4 | 31184484 | A | G | -0.011 | 0.002 | 5.03E-12 |
| Smoking | rs77307359 | 4 | 34839767 | C | T | -0.011 | 0.002 | 2.07E-08 |
| Smoking | rs7666804 | 4 | 57750148 | C | T | -0.011 | 0.002 | 1.18E-08 |
| Smoking | rs993700 | 4 | 67825894 | C | T | -0.014 | 0.002 | 1.00E-13 |
| Smoking | rs1503211 | 4 | 94079508 | A | G | 0.010 | 0.002 | 3.43E-10 |
| Smoking | rs3934797 | 4 | 112467612 | A | G | -0.015 | 0.002 | 1.90E-13 |
| Smoking | rs13145728 | 4 | 140927812 | C | G | -0.012 | 0.002 | 6.87E-14 |
| Smoking | rs10001365 | 4 | 147797214 | A | G | -0.015 | 0.002 | 4.32E-21 |
| Smoking | rs1490683 | 4 | 155362876 | C | T | -0.010 | 0.002 | 2.84E-08 |
| Smoking | rs6852117 | 4 | 173076888 | G | C | -0.012 | 0.002 | 1.97E-13 |
| Smoking | rs13162305 | 5 | 12122698 | T | A | 0.010 | 0.002 | 1.15E-08 |
| Smoking | rs6868892 | 5 | 22193780 | T | C | 0.010 | 0.002 | 1.92E-08 |
| Smoking | rs1392446 | 5 | 30831387 | T | C | 0.010 | 0.002 | 5.60E-10 |
| Smoking | rs71627577 | 5 | 43125795 | G | A | -0.017 | 0.002 | 5.15E-12 |
| Smoking | rs6861333 | 5 | 50703933 | G | C | -0.011 | 0.002 | 3.12E-10 |
| Smoking | rs9763225 | 5 | 60257450 | G | A | 0.014 | 0.002 | 5.38E-12 |
| Smoking | rs10062607 | 5 | 79290634 | A | C | 0.010 | 0.002 | 2.28E-10 |
| Smoking | rs4571506 | 5 | 87756918 | T | C | -0.015 | 0.002 | 1.64E-21 |
| Smoking | rs11742625 | 5 | 91396113 | C | T | 0.016 | 0.002 | 2.19E-10 |
| Smoking | rs72789627 | 5 | 106826477 | T | C | -0.017 | 0.002 | 1.54E-13 |
| Smoking | rs288181 | 5 | 107349285 | T | C | -0.009 | 0.002 | 4.11E-08 |
| Smoking | rs2313500 | 5 | 154808532 | T | C | 0.013 | 0.002 | 1.99E-12 |
| Smoking | rs4044321 | 5 | 166989513 | G | A | -0.016 | 0.002 | 3.04E-23 |
| Smoking | rs2173019 | 5 | 167614971 | A | T | 0.013 | 0.002 | 3.47E-10 |
| Smoking | rs39784 | 5 | 170559066 | A | C | 0.012 | 0.002 | 6.41E-11 |
| Smoking | rs853684 | 6 | 28294550 | C | T | -0.012 | 0.002 | 8.55E-13 |
| Smoking | rs12209775 | 6 | 37372370 | G | C | -0.010 | 0.002 | 1.29E-10 |
| Smoking | rs111861749 | 6 | 50889019 | A | G | -0.018 | 0.003 | 3.16E-12 |
| Smoking | rs160631 | 6 | 52895230 | G | T | -0.011 | 0.002 | 3.56E-09 |
| Smoking | rs16896199 | 6 | 65787895 | T | A | 0.012 | 0.002 | 5.61E-09 |
| Smoking | rs146628116 | 6 | 67551428 | A | T | 0.014 | 0.002 | 1.43E-14 |
| Smoking | rs62419578 | 6 | 84356959 | A | T | 0.013 | 0.002 | 1.08E-08 |
| Smoking | rs9375371 | 6 | 98751680 | A | G | 0.013 | 0.002 | 1.41E-13 |
| Smoking | rs11756490 | 6 | 100341774 | A | T | -0.014 | 0.002 | 6.20E-09 |
| Smoking | rs846781 | 6 | 101280434 | C | T | -0.012 | 0.002 | 4.80E-11 |
| Smoking | rs557544 | 6 | 109020332 | C | T | -0.009 | 0.002 | 2.82E-08 |
| Smoking | rs465646 | 6 | 111620758 | A | G | -0.024 | 0.002 | 1.70E-28 |
| Smoking | rs9402093 | 6 | 129353671 | T | G | 0.010 | 0.002 | 3.38E-09 |
| Smoking | rs2876586 | 6 | 144862998 | A | G | 0.009 | 0.002 | 1.88E-08 |
| Smoking | rs4401691 | 6 | 157720414 | G | C | -0.010 | 0.002 | 9.42E-09 |
| Smoking | rs10698713 | 6 | 158882320 | A | G | -0.020 | 0.004 | 1.84E-08 |
| Smoking | rs79222572 | 6 | 165108555 | G | T | 0.010 | 0.002 | 1.53E-08 |
| Smoking | rs6464024 | 7 | 1688369 | T | C | -0.012 | 0.002 | 3.38E-14 |
| Smoking | rs10950410 | 7 | 1909086 | G | C | -0.011 | 0.002 | 1.54E-12 |
| Smoking | rs13246563 | 7 | 3438243 | G | C | -0.013 | 0.002 | 3.99E-14 |
| Smoking | rs2237303 | 7 | 21483605 | A | G | -0.010 | 0.002 | 6.37E-10 |
| Smoking | rs12112638 | 7 | 69735251 | G | A | -0.012 | 0.002 | 8.74E-12 |
| Smoking | rs7788527 | 7 | 70579487 | C | T | 0.010 | 0.002 | 1.58E-08 |
| Smoking | rs2072155 | 7 | 77762457 | C | T | 0.013 | 0.002 | 1.16E-13 |
| Smoking | rs3814994 | 7 | 88427385 | T | G | 0.010 | 0.002 | 7.44E-09 |
| Smoking | rs3801289 | 7 | 96638267 | C | A | -0.012 | 0.002 | 3.64E-12 |
| Smoking | rs7804551 | 7 | 99119110 | G | A | -0.015 | 0.002 | 1.09E-12 |
| Smoking | rs6959670 | 7 | 110909169 | T | C | 0.010 | 0.002 | 7.29E-09 |
| Smoking | rs6973168 | 7 | 114963829 | G | C | -0.009 | 0.002 | 2.70E-09 |
| Smoking | rs10233018 | 7 | 117523709 | G | A | 0.014 | 0.002 | 1.49E-18 |
| Smoking | rs1899689 | 7 | 121964349 | T | C | 0.009 | 0.002 | 1.72E-08 |
| Smoking | rs2402821 | 7 | 126297855 | A | G | 0.010 | 0.002 | 3.51E-09 |
| Smoking | rs10279261 | 7 | 133589846 | A | G | -0.012 | 0.002 | 6.87E-14 |
| Smoking | rs7830359 | 8 | 10834258 | T | C | -0.010 | 0.002 | 3.88E-09 |
| Smoking | rs1565735 | 8 | 27426077 | A | T | -0.023 | 0.002 | 7.49E-32 |
| Smoking | rs7829715 | 8 | 59803836 | C | T | -0.012 | 0.002 | 1.30E-13 |
| Smoking | rs4579569 | 8 | 64913762 | A | G | 0.012 | 0.002 | 1.23E-14 |
| Smoking | rs6472232 | 8 | 66792632 | G | T | -0.009 | 0.002 | 1.43E-08 |
| Smoking | rs13261725 | 8 | 91866297 | C | G | 0.013 | 0.002 | 3.43E-13 |
| Smoking | rs9987376 | 8 | 93190014 | G | T | -0.014 | 0.002 | 1.02E-17 |
| Smoking | rs13255625 | 8 | 144269567 | G | A | 0.012 | 0.002 | 4.03E-12 |
| Smoking | rs4543592 | 9 | 3014254 | C | T | 0.012 | 0.002 | 2.51E-14 |
| Smoking | rs12685816 | 9 | 8290929 | A | G | 0.015 | 0.002 | 1.66E-10 |
| Smoking | rs10121930 | 9 | 11175974 | A | T | 0.010 | 0.002 | 8.01E-10 |
| Smoking | rs56348592 | 9 | 16749265 | G | A | 0.013 | 0.002 | 1.81E-08 |
| Smoking | rs10119117 | 9 | 29740028 | T | C | 0.009 | 0.002 | 1.80E-08 |
| Smoking | rs72733235 | 9 | 38275772 | C | T | 0.013 | 0.002 | 1.86E-09 |
| Smoking | rs1246265 | 9 | 86761745 | C | T | 0.012 | 0.002 | 1.27E-11 |
| Smoking | rs4837631 | 9 | 122061948 | T | C | -0.009 | 0.002 | 3.54E-09 |
| Smoking | rs13301073 | 9 | 128284378 | A | G | 0.011 | 0.002 | 3.13E-11 |
| Smoking | rs11103667 | 9 | 137978360 | T | C | 0.013 | 0.002 | 1.47E-10 |
| Smoking | rs1334557 | 10 | 8790819 | T | C | 0.013 | 0.002 | 1.51E-12 |
| Smoking | rs7092291 | 10 | 10040004 | T | C | -0.010 | 0.002 | 7.74E-10 |
| Smoking | rs1291865 | 10 | 11082192 | T | G | 0.012 | 0.002 | 2.12E-13 |
| Smoking | rs11258417 | 10 | 13533053 | T | C | -0.010 | 0.002 | 3.83E-10 |
| Smoking | rs11012726 | 10 | 21797272 | C | T | 0.014 | 0.002 | 4.25E-17 |
| Smoking | rs1776631 | 10 | 31422577 | C | T | -0.011 | 0.002 | 3.92E-08 |
| Smoking | rs1733756 | 10 | 56700221 | G | A | 0.010 | 0.002 | 1.23E-10 |
| Smoking | rs13377168 | 10 | 63675271 | A | T | -0.015 | 0.002 | 2.16E-20 |
| Smoking | rs60453921 | 10 | 87362579 | A | T | 0.013 | 0.002 | 1.06E-09 |
| Smoking | rs3781295 | 10 | 104140602 | A | G | -0.012 | 0.002 | 1.13E-12 |
| Smoking | rs10786721 | 10 | 104654383 | A | C | 0.018 | 0.002 | 7.91E-27 |
| Smoking | rs11192160 | 10 | 106453550 | C | G | 0.014 | 0.002 | 8.66E-10 |
| Smoking | rs4751614 | 10 | 118696266 | T | A | 0.012 | 0.002 | 6.35E-11 |
| Smoking | rs911781 | 10 | 123966989 | G | A | -0.009 | 0.002 | 4.99E-08 |
| Smoking | rs9423279 | 10 | 125680419 | G | C | -0.011 | 0.002 | 9.64E-11 |
| Smoking | rs10444314 | 11 | 7951242 | G | T | -0.009 | 0.002 | 1.77E-08 |
| Smoking | rs35891966 | 11 | 20129311 | A | G | -0.019 | 0.003 | 6.52E-10 |
| Smoking | rs6265 | 11 | 27679916 | T | C | -0.020 | 0.002 | 1.86E-22 |
| Smoking | rs2939756 | 11 | 41436297 | A | G | -0.010 | 0.002 | 6.87E-11 |
| Smoking | rs72904370 | 11 | 46040015 | C | G | -0.011 | 0.002 | 1.59E-08 |
| Smoking | rs574835 | 11 | 64110668 | A | G | -0.010 | 0.002 | 2.33E-10 |
| Smoking | rs7947391 | 11 | 66186882 | G | A | 0.009 | 0.002 | 7.55E-09 |
| Smoking | rs4944844 | 11 | 73311705 | C | T | -0.013 | 0.002 | 2.33E-10 |
| Smoking | rs7929518 | 11 | 85980958 | G | A | 0.012 | 0.002 | 9.06E-10 |
| Smoking | rs7127712 | 11 | 112874603 | T | A | 0.027 | 0.002 | 2.19E-65 |
| Smoking | rs540860 | 11 | 121530888 | G | A | 0.012 | 0.002 | 3.18E-13 |
| Smoking | rs71491832 | 11 | 124611997 | G | C | -0.018 | 0.003 | 3.72E-10 |
| Smoking | rs540356 | 11 | 132203816 | A | C | 0.012 | 0.002 | 4.07E-13 |
| Smoking | rs551739 | 12 | 40414075 | T | A | 0.010 | 0.002 | 4.42E-09 |
| Smoking | rs2292239 | 12 | 56482180 | G | T | 0.011 | 0.002 | 1.03E-10 |
| Smoking | rs7969559 | 12 | 69655167 | G | A | -0.011 | 0.002 | 3.28E-10 |
| Smoking | rs1111578 | 12 | 117913256 | T | G | -0.013 | 0.002 | 8.22E-09 |
| Smoking | rs1971318 | 12 | 121389500 | T | C | 0.016 | 0.002 | 7.80E-13 |
| Smoking | rs404263 | 12 | 125801308 | T | C | -0.011 | 0.002 | 1.55E-10 |
| Smoking | rs7986094 | 13 | 31029931 | C | A | 0.010 | 0.002 | 5.99E-09 |
| Smoking | rs61959481 | 13 | 55834929 | A | G | -0.012 | 0.002 | 2.06E-10 |
| Smoking | rs9538536 | 13 | 60536321 | G | T | -0.009 | 0.002 | 4.28E-08 |
| Smoking | rs9529052 | 13 | 66940097 | A | T | -0.012 | 0.002 | 1.85E-13 |
| Smoking | rs9541499 | 13 | 69261072 | G | T | -0.017 | 0.003 | 2.16E-08 |
| Smoking | rs2783130 | 13 | 80170160 | G | A | -0.009 | 0.002 | 2.71E-08 |
| Smoking | rs8001839 | 13 | 97110073 | G | A | 0.009 | 0.002 | 3.74E-08 |
| Smoking | rs7333559 | 13 | 100546450 | A | G | -0.014 | 0.002 | 3.24E-13 |
| Smoking | rs7984311 | 13 | 100741752 | A | G | 0.009 | 0.002 | 1.77E-08 |
| Smoking | rs2022815 | 14 | 28356733 | A | G | 0.010 | 0.002 | 1.12E-09 |
| Smoking | rs66480876 | 14 | 32448189 | G | C | 0.009 | 0.002 | 1.38E-08 |
| Smoking | rs9323328 | 14 | 58653514 | G | A | -0.011 | 0.002 | 7.25E-12 |
| Smoking | rs17594561 | 14 | 79618750 | G | A | 0.010 | 0.002 | 1.27E-09 |
| Smoking | rs1381287 | 14 | 98597552 | T | C | 0.012 | 0.002 | 7.51E-15 |
| Smoking | rs11626595 | 14 | 104593623 | T | C | -0.015 | 0.002 | 2.73E-12 |
| Smoking | rs1435679 | 15 | 36399245 | A | G | 0.010 | 0.002 | 2.86E-10 |
| Smoking | rs34488670 | 15 | 47684936 | C | T | 0.018 | 0.002 | 8.73E-20 |
| Smoking | rs2711607 | 15 | 54117442 | T | G | 0.011 | 0.002 | 4.09E-08 |
| Smoking | rs2289791 | 15 | 67476952 | T | G | -0.013 | 0.002 | 2.87E-12 |
| Smoking | rs11632439 | 15 | 80987012 | G | A | 0.009 | 0.002 | 1.31E-08 |
| Smoking | rs12441907 | 15 | 83922387 | A | C | -0.015 | 0.002 | 6.09E-14 |
| Smoking | rs1155641 | 15 | 97502995 | A | G | 0.010 | 0.002 | 7.72E-10 |
| Smoking | rs6598539 | 15 | 99204483 | C | T | 0.011 | 0.002 | 5.76E-12 |
| Smoking | rs763053 | 16 | 735921 | C | T | -0.015 | 0.002 | 6.71E-16 |
| Smoking | rs11865123 | 16 | 5840465 | A | T | 0.011 | 0.002 | 4.10E-08 |
| Smoking | rs56225373 | 16 | 13755408 | G | T | 0.016 | 0.003 | 3.13E-08 |
| Smoking | rs12923427 | 16 | 17575065 | T | C | -0.014 | 0.002 | 4.96E-12 |
| Smoking | rs3867541 | 16 | 18293027 | A | G | -0.010 | 0.002 | 9.15E-09 |
| Smoking | rs6497840 | 16 | 25351633 | A | G | 0.013 | 0.002 | 1.13E-12 |
| Smoking | rs55942317 | 16 | 49626772 | A | G | 0.017 | 0.003 | 4.08E-11 |
| Smoking | rs4785187 | 16 | 49766772 | A | G | 0.012 | 0.002 | 2.03E-10 |
| Smoking | rs7205551 | 16 | 69605968 | A | G | 0.010 | 0.002 | 1.15E-09 |
| Smoking | rs114900182 | 16 | 72629056 | G | C | -0.021 | 0.003 | 1.45E-12 |
| Smoking | rs4888444 | 16 | 75690279 | G | A | -0.025 | 0.004 | 6.84E-10 |
| Smoking | rs1050847 | 16 | 87443734 | T | C | -0.011 | 0.002 | 3.71E-11 |
| Smoking | rs7195043 | 16 | 90020861 | T | C | -0.009 | 0.002 | 2.13E-08 |
| Smoking | rs8067305 | 17 | 1975657 | A | G | 0.011 | 0.002 | 4.95E-12 |
| Smoking | rs11078713 | 17 | 7795972 | G | A | -0.013 | 0.002 | 2.77E-15 |
| Smoking | rs7224742 | 17 | 30657058 | T | C | -0.011 | 0.002 | 1.02E-11 |
| Smoking | rs8069451 | 17 | 37504933 | C | T | 0.011 | 0.002 | 1.02E-09 |
| Smoking | rs11656151 | 17 | 44068492 | G | A | -0.013 | 0.002 | 5.61E-12 |
| Smoking | rs12600466 | 17 | 50306926 | T | A | 0.012 | 0.002 | 1.30E-11 |
| Smoking | rs745570 | 17 | 77781725 | G | A | -0.010 | 0.002 | 8.70E-11 |
| Smoking | rs12945403 | 17 | 80004094 | C | T | -0.009 | 0.002 | 1.79E-08 |
| Smoking | rs888292 | 18 | 25210972 | T | A | -0.011 | 0.002 | 2.03E-09 |
| Smoking | rs3110590 | 18 | 27808203 | A | C | 0.011 | 0.002 | 1.22E-09 |
| Smoking | rs16975171 | 18 | 39269650 | A | C | -0.017 | 0.003 | 5.29E-09 |
| Smoking | rs11873164 | 18 | 42659922 | T | C | -0.016 | 0.002 | 1.63E-12 |
| Smoking | rs1025910 | 18 | 49871340 | C | G | -0.012 | 0.002 | 4.83E-13 |
| Smoking | rs1945737 | 18 | 53744545 | C | T | -0.010 | 0.002 | 8.19E-10 |
| Smoking | rs3213876 | 18 | 73183978 | C | T | 0.012 | 0.002 | 5.08E-12 |
| Smoking | rs71367545 | 18 | 77576337 | A | G | 0.012 | 0.002 | 1.70E-10 |
| Smoking | rs76608582 | 19 | 4474725 | A | C | -0.027 | 0.004 | 2.76E-13 |
| Smoking | rs58547189 | 19 | 18462708 | A | G | -0.011 | 0.002 | 1.73E-08 |
| Smoking | rs172032 | 19 | 18633755 | C | T | 0.009 | 0.002 | 1.56E-08 |
| Smoking | rs11673452 | 19 | 33928001 | C | T | 0.015 | 0.003 | 3.40E-09 |
| Smoking | rs1126757 | 19 | 55879872 | T | C | 0.010 | 0.002 | 4.15E-11 |
| Smoking | rs192477591 | 20 | 19659362 | T | C | 0.012 | 0.002 | 1.73E-08 |
| Smoking | rs6141314 | 20 | 31093514 | A | G | 0.013 | 0.002 | 3.59E-13 |
| Smoking | rs6088618 | 20 | 33409350 | A | G | -0.011 | 0.002 | 5.89E-11 |
| Smoking | rs56820925 | 20 | 54387374 | T | C | -0.011 | 0.002 | 2.25E-10 |
| Smoking | rs78175438 | 21 | 40663255 | C | T | 0.016 | 0.002 | 2.02E-11 |
| Smoking | rs4819027 | 21 | 46495224 | G | C | -0.010 | 0.002 | 1.34E-08 |
| Smoking | rs9613472 | 22 | 27972479 | G | A | 0.009 | 0.002 | 4.02E-08 |
| Smoking | rs134529 | 22 | 28781758 | C | T | -0.012 | 0.002 | 8.86E-13 |
| Smoking | rs5751239 | 22 | 42592239 | T | C | -0.010 | 0.002 | 1.02E-10 |
| Smoking | rs9627272 | 22 | 46442288 | C | G | -0.010 | 0.002 | 1.28E-09 |
| Drinking | rs2093186 | 1 | 16000106 | T | C | -0.012 | 0.002 | 1.01E-09 |
| Drinking | rs12044012 | 1 | 33816726 | A | G | -0.012 | 0.002 | 1.34E-09 |
| Drinking | rs6698883 | 1 | 34368735 | T | C | -0.016 | 0.003 | 1.14E-08 |
| Drinking | rs12121630 | 1 | 50977279 | A | G | -0.014 | 0.002 | 1.59E-08 |
| Drinking | rs2310752 | 1 | 66392405 | A | G | -0.010 | 0.002 | 2.57E-08 |
| Drinking | rs10753661 | 1 | 165119792 | A | G | -0.011 | 0.002 | 1.25E-09 |
| Drinking | rs28680958 | 1 | 173848808 | A | G | -0.014 | 0.002 | 1.08E-10 |
| Drinking | rs823099 | 1 | 205669322 | A | C | 0.011 | 0.002 | 2.31E-09 |
| Drinking | rs6531148 | 2 | 16650233 | C | T | -0.012 | 0.002 | 7.58E-09 |
| Drinking | rs1260326 | 2 | 27730940 | C | T | 0.025 | 0.002 | 3.16E-46 |
| Drinking | rs75199129 | 2 | 44298775 | T | A | -0.028 | 0.004 | 8.23E-12 |
| Drinking | rs1004787 | 2 | 45159091 | A | G | 0.017 | 0.002 | 1.13E-22 |
| Drinking | rs68084872 | 2 | 58082205 | A | G | -0.011 | 0.002 | 6.40E-09 |
| Drinking | rs6739804 | 2 | 63269604 | C | T | -0.014 | 0.002 | 2.85E-13 |
| Drinking | rs828867 | 2 | 74334462 | A | G | 0.010 | 0.002 | 3.44E-09 |
| Drinking | rs11692435 | 2 | 98275354 | A | G | 0.019 | 0.003 | 7.20E-11 |
| Drinking | rs13024996 | 2 | 144225215 | A | C | -0.014 | 0.002 | 5.18E-14 |
| Drinking | rs3768650 | 2 | 152990932 | G | A | -0.011 | 0.002 | 1.67E-08 |
| Drinking | rs7588444 | 2 | 178185364 | C | T | 0.013 | 0.002 | 1.76E-09 |
| Drinking | rs60026303 | 3 | 81285635 | G | A | 0.012 | 0.002 | 3.58E-08 |
| Drinking | rs28732378 | 3 | 85403892 | G | A | -0.020 | 0.002 | 1.55E-23 |
| Drinking | rs147711594 | 3 | 100893355 | T | G | -0.030 | 0.005 | 2.91E-08 |
| Drinking | rs6787172 | 3 | 158187811 | G | T | -0.011 | 0.002 | 7.49E-10 |
| Drinking | rs9875033 | 3 | 184028428 | C | T | 0.012 | 0.002 | 7.90E-10 |
| Drinking | rs12646808 | 4 | 3249828 | C | T | -0.011 | 0.002 | 2.47E-09 |
| Drinking | rs111203819 | 4 | 18371143 | G | T | 0.010 | 0.002 | 2.41E-08 |
| Drinking | rs11940694 | 4 | 39414993 | G | A | 0.028 | 0.002 | 9.51E-56 |
| Drinking | rs16854020 | 4 | 42117559 | A | G | 0.019 | 0.003 | 6.28E-13 |
| Drinking | rs1229984 | 4 | 100239319 | C | T | 0.193 | 0.005 | 1.17E-283 |
| Drinking | rs62305763 | 4 | 100289405 | T | C | 0.030 | 0.003 | 1.15E-28 |
| Drinking | rs13107325 | 4 | 103188709 | T | C | -0.039 | 0.004 | 2.86E-28 |
| Drinking | rs2079106 | 4 | 113282950 | G | C | 0.010 | 0.002 | 3.38E-08 |
| Drinking | rs11943397 | 4 | 143617304 | C | T | 0.012 | 0.002 | 1.23E-11 |
| Drinking | rs28694391 | 4 | 174154311 | C | T | -0.013 | 0.002 | 3.81E-09 |
| Drinking | rs4481304 | 5 | 50759415 | A | G | -0.011 | 0.002 | 1.25E-09 |
| Drinking | rs4916723 | 5 | 87854395 | C | A | -0.011 | 0.002 | 2.74E-10 |
| Drinking | rs56353702 | 5 | 132248575 | G | A | -0.014 | 0.003 | 2.16E-08 |
| Drinking | rs6887908 | 5 | 144383685 | C | A | -0.010 | 0.002 | 4.47E-08 |
| Drinking | rs55872084 | 5 | 155902003 | T | G | 0.012 | 0.002 | 4.68E-09 |
| Drinking | rs6899302 | 5 | 166846529 | C | T | -0.010 | 0.002 | 1.70E-09 |
| Drinking | rs28361092 | 6 | 33443983 | A | G | -0.013 | 0.002 | 3.42E-09 |
| Drinking | rs1906252 | 6 | 98550289 | A | C | 0.010 | 0.002 | 2.05E-08 |
| Drinking | rs6962879 | 7 | 14295168 | G | C | 0.010 | 0.002 | 1.89E-08 |
| Drinking | rs11238438 | 7 | 51358762 | C | G | 0.010 | 0.002 | 3.73E-08 |
| Drinking | rs13236841 | 7 | 69389859 | G | A | -0.014 | 0.002 | 1.92E-12 |
| Drinking | rs10236149 | 7 | 98977515 | G | A | -0.016 | 0.003 | 4.40E-10 |
| Drinking | rs10276148 | 7 | 103733264 | A | G | 0.012 | 0.002 | 1.19E-11 |
| Drinking | rs322773 | 7 | 127762119 | G | A | 0.010 | 0.002 | 1.48E-08 |
| Drinking | rs2533126 | 7 | 153473086 | A | G | 0.013 | 0.002 | 1.98E-14 |
| Drinking | rs1838420 | 8 | 64960855 | T | G | 0.010 | 0.002 | 1.53E-08 |
| Drinking | rs10956823 | 8 | 93140909 | T | G | 0.012 | 0.002 | 8.38E-09 |
| Drinking | rs800578 | 8 | 116869477 | C | T | 0.011 | 0.002 | 4.51E-08 |
| Drinking | rs28601761 | 8 | 126500031 | G | C | 0.011 | 0.002 | 1.57E-10 |
| Drinking | rs55932213 | 9 | 108755622 | G | A | 0.013 | 0.002 | 1.61E-10 |
| Drinking | rs36123652 | 9 | 109338067 | G | A | -0.017 | 0.003 | 5.31E-10 |
| Drinking | rs13288470 | 9 | 129688559 | T | A | -0.016 | 0.003 | 4.05E-09 |
| Drinking | rs61873510 | 10 | 102626510 | T | G | -0.011 | 0.002 | 1.17E-08 |
| Drinking | rs6584893 | 10 | 110504315 | C | A | -0.011 | 0.002 | 3.17E-08 |
| Drinking | rs10743083 | 11 | 8636350 | G | A | -0.015 | 0.002 | 1.64E-10 |
| Drinking | rs2049045 | 11 | 27694241 | C | G | -0.015 | 0.002 | 2.24E-11 |
| Drinking | rs11039216 | 11 | 47406592 | T | C | 0.016 | 0.002 | 3.76E-19 |
| Drinking | rs4337071 | 11 | 113334100 | T | C | -0.015 | 0.002 | 5.59E-17 |
| Drinking | rs11607622 | 11 | 113526665 | T | C | 0.015 | 0.003 | 2.22E-08 |
| Drinking | rs530916 | 11 | 121549091 | G | A | 0.011 | 0.002 | 4.22E-10 |
| Drinking | rs56115085 | 12 | 6853615 | T | C | -0.013 | 0.002 | 4.14E-08 |
| Drinking | rs35011311 | 12 | 38616581 | T | G | -0.011 | 0.002 | 8.95E-09 |
| Drinking | rs4761961 | 12 | 51799968 | C | A | -0.011 | 0.002 | 5.19E-10 |
| Drinking | rs3809162 | 12 | 54674235 | G | A | 0.010 | 0.002 | 6.84E-09 |
| Drinking | rs61934664 | 12 | 81608058 | A | G | -0.012 | 0.002 | 8.96E-12 |
| Drinking | rs2087975 | 12 | 92074004 | G | A | -0.011 | 0.002 | 2.02E-09 |
| Drinking | rs34484751 | 12 | 123652527 | C | A | 0.031 | 0.005 | 7.86E-10 |
| Drinking | rs34704785 | 13 | 68117681 | T | C | -0.010 | 0.002 | 4.52E-09 |
| Drinking | rs8020892 | 14 | 58724089 | A | G | -0.013 | 0.002 | 2.06E-12 |
| Drinking | rs28929474 | 14 | 94844947 | T | C | -0.049 | 0.006 | 3.28E-14 |
| Drinking | rs9745238 | 15 | 49552601 | G | C | -0.013 | 0.002 | 1.38E-09 |
| Drinking | rs7162115 | 15 | 52291273 | T | G | 0.010 | 0.002 | 2.65E-08 |
| Drinking | rs35807116 | 15 | 74667953 | T | C | 0.011 | 0.002 | 2.65E-09 |
| Drinking | rs28616142 | 15 | 86867867 | T | C | 0.011 | 0.002 | 2.99E-10 |
| Drinking | rs72770409 | 16 | 24802331 | T | C | -0.024 | 0.004 | 3.16E-09 |
| Drinking | rs153106 | 16 | 28526897 | C | T | -0.016 | 0.002 | 9.38E-19 |
| Drinking | rs1558902 | 16 | 53803574 | A | T | -0.012 | 0.002 | 6.42E-11 |
| Drinking | rs11075711 | 16 | 69266456 | T | C | -0.013 | 0.002 | 1.31E-08 |
| Drinking | rs79616692 | 16 | 72338507 | C | G | 0.020 | 0.003 | 3.54E-12 |
| Drinking | rs11860773 | 16 | 73912503 | C | T | -0.016 | 0.002 | 1.04E-12 |
| Drinking | rs13332432 | 16 | 85721809 | G | C | 0.013 | 0.002 | 1.64E-11 |
| Drinking | rs11078696 | 17 | 7459299 | T | G | 0.020 | 0.003 | 4.29E-12 |
| Drinking | rs34121753 | 17 | 7733833 | G | A | 0.011 | 0.002 | 1.53E-09 |
| Drinking | rs1971157 | 17 | 27925343 | C | G | 0.010 | 0.002 | 2.74E-08 |
| Drinking | rs147431626 | 17 | 44335635 | A | G | -0.029 | 0.002 | 7.52E-32 |
| Drinking | rs148390057 | 18 | 37695780 | T | C | -0.011 | 0.002 | 2.91E-08 |
| Drinking | rs4890444 | 18 | 40747457 | G | C | 0.011 | 0.002 | 1.89E-09 |
| Drinking | rs1011392 | 18 | 53041903 | G | A | -0.010 | 0.002 | 1.78E-08 |
| Drinking | rs1942964 | 18 | 55027212 | G | T | -0.011 | 0.002 | 3.89E-09 |
| Drinking | rs838145 | 19 | 49248730 | A | G | -0.016 | 0.002 | 5.62E-21 |
| Drinking | rs55987845 | 20 | 18656930 | T | C | -0.010 | 0.002 | 3.41E-08 |
| Drinking | rs2424645 | 20 | 24720026 | G | A | -0.012 | 0.002 | 1.15E-10 |
| Drinking | rs7284839 | 22 | 42010902 | T | C | 0.016 | 0.003 | 3.66E-08 |
| Drinking | rs17884691 | 22 | 46481623 | A | G | -0.011 | 0.002 | 2.91E-08 |
| MVPA | rs6427178 | 1 | 169095082 | A | G | 0.023 | 0.004 | 1.71E-08 |
| MVPA | rs1160545 | 2 | 100832269 | T | C | 0.025 | 0.004 | 1.73E-09 |
| MVPA | rs336620 | 3 | 18628793 | C | G | 0.024 | 0.004 | 4.05E-08 |
| MVPA | rs7613360 | 3 | 49916710 | T | C | -0.025 | 0.004 | 2.77E-09 |
| MVPA | rs1691471 | 3 | 85011013 | T | C | 0.038 | 0.004 | 1.73E-19 |
| MVPA | rs2668196 | 3 | 165502709 | A | T | -0.023 | 0.004 | 2.09E-08 |
| MVPA | rs4865512 | 5 | 50661601 | A | G | 0.024 | 0.004 | 7.68E-09 |
| MVPA | rs4352559 | 5 | 60586625 | T | C | 0.018 | 0.003 | 1.65E-08 |
| MVPA | rs370935521 | 6 | 26770791 | T | C | 0.043 | 0.008 | 8.25E-09 |
| MVPA | rs13201721 | 6 | 141799534 | T | C | 0.026 | 0.004 | 1.83E-10 |
| MVPA | rs12357890 | 10 | 99762693 | A | G | 0.023 | 0.004 | 4.77E-08 |
| MVPA | rs1424751 | 11 | 57479732 | C | G | -0.025 | 0.004 | 2.53E-08 |
| MVPA | rs1625595 | 11 | 66078129 | T | C | -0.021 | 0.003 | 1.90E-11 |
| MVPA | rs568546 | 11 | 107321156 | T | C | 0.024 | 0.004 | 5.89E-09 |
| MVPA | rs385301 | 17 | 19806828 | T | C | -0.028 | 0.005 | 1.60E-09 |
| MVPA | rs9903845 | 17 | 50291181 | A | C | -0.020 | 0.003 | 6.05E-09 |

BMI, body mass index; EA, effect allele; MVPA, moderate-to-vigorous intensity physical activity; NEA, non-effect allele; SBP, systolic blood pressure; SE, standard error; SNP, single nucleotide polymorphism.
