# Supplementary material for: Factorial Mendelian randomization of lipoprotein (a) lowering, low-density lipoprotein cholesterol lowering, and lifestyle improvements: joint associations with cardiovascular risk
Source: Int J Epidemiol. 2025 Mar 10;54(2):dyaf020. doi: 10.1093/ije/dyaf020 (PMC11893152; doi:10.1093/ije/dyaf020)
Supplement: Supplementary file 1 [file dyaf020_supplementary_data.zip › ije-2024-03-0366-File008.pdf]

Supplementary Tables

Supplementary Table S1. Definitions of studied outcomes............................2

Supplementary Table S2. Genetic variants included in lipoprotein(a) [Lp(a)] genetic score..3

Supplementary Table S3. Drug target variants included in the genetic score and their associations with low-density lipoprotein cholesterol (LDL-C) levels in the Global Lipids Genetics Consortium...5

Supplementary Table S4. Genetic variants included in low-density lipoprotein cholesterol (LDL-C) genetic score..9

Supplementary Table S5. Detailed information about genetic instruments used to proxy body mass index (BMI), systolic blood pressure (SBP), and lifestyle factors..15

Supplementary Table S6. Genetic instruments used to calculate genetic scores for body mass index (BMI), systolic blood pressure (SBP), and lifestyle factors..16

Supplementary Table S1. Definitions of studied outcomes.

Outcome	ICD-9	ICD-10
Cardiovascular disease (CVD)	390-398, 410-417, 420-438, 440, 443, 444	I00-I09, I20-I70, I73-I74
Coronary artery disease (CAD)	410-415, 428, 429	I20-25
Peripheral arterial disease (PAD)	443	I731, I738, I739
Stroke	430, 431, 434, 436	I60-I63
Subarachnoid hemorrhage (SAH)	430	I60
Intracerebral hemorrhage (ICH)	431	I61
Ischemic stroke (IS)	434, 436	I63, I64
Atrial fibrillation (AF)	427.3	I48
Heart failure (HF)	428	I50
Venous thromboembolism (VTE)	415.1, 444	I26, I74
Cardiovascular-specific death	390-398, 410-417, 420-438, 440, 443, 444	I00-I09, I20-I70, I73-I74
3-point major adverse cardiovascular events (MACE)	410, 430, 431-434, 436	I00-I09, I20-I70, I73-I74
4-point major adverse cardiovascular events (MACE)	410, 428, 430, 431-434, 436	I00-I09, I20-I70, I73-I74

Supplementary Table S2. Genetic variants included in lipoprotein(a) [Lp(a)] genetic score.

SNP	Chr	Position	EA	NEA	Beta	SE
rs200144324	6	160493099	T	C	81.5	11.3
rs200684404	6	160543317	T	C	67.7	9.2
rs183815886	6	160720804	C	G	14.8	1.8
rs143365644	6	160825930	T	A	3.7	0.5
rs151135411	6	160831796	A	G	69.5	2.7
rs117446263	6	160847571	A	G	-5.2	0.6
rs6916433	6	160890350	T	A	-4.7	0.3
rs182443492	6	160891897	A	C	36.8	1.0
rs139609547	6	160899049	-	A	4.4	0.4
rs141834709	6	160909667	A	T	8.7	1.0
rs142126734	6	160942926	A	G	7.5	0.5
rs41266379	6	160953137	C	T	7.1	0.7
rs41267809	6	160953642	G	A	-6.6	0.6
rs143461353	6	160954800	T	C	13.1	1.0
rs139145675	6	160966559	A	G	-22.5	2.4
rs74617384	6	160997118	T	A	42.4	0.5
rs41267813	6	160998199	A	G	-58.8	2.9
rs200376184	6	161011999	C	G	17.5	2.7
rs9456551	6	161012805	C	T	3.6	0.2
rs140570886	6	161013013	C	T	80.2	0.8
rs73596816	6	161017363	A	G	19.2	0.6
rs41259144	6	161022107	T	C	-9.6	0.8
rs191690882	6	161031132	A	G	-13.2	1.9
rs369686024	6	161032800	A	G	19.2	0.8
rs41269876	6	161070653	A	C	-8.2	0.6
rs79246098	6	161078894	C	T	6.2	0.9
rs77337569	6	161087652	G	T	5.2	0.8
rs75274517	6	161088956	A	G	-6.5	1.0
rs56393506	6	161089307	T	C	12.4	0.4
rs139389770	6	161135746	G	T	-5.2	0.9
rs783147	6	161137990	A	G	-2.0	0.3
rs4252152	6	161159366	G	T	9.1	0.9
rs1835346	6	161162290	G	A	5.2	0.7
rs4252170	6	161162406	C	T	3.2	0.4
rs117534432	6	161177443	T	C	3.3	0.5
rs11753588	6	161189071	A	G	-2.4	0.3
rs186418835	6	161214526	A	G	-9.7	1.5
rs140606700	6	161250301	G	A	6.4	1.2
rs138491411	6	161251940	G	A	5.0	0.8

rs182349273	6	161255668	G	A	34.4	5.7
rs34371670	6	161257953	T	C	-8.4	0.7
rs4709474	6	161285760	G	A	1.7	0.2
rs145099029	6	161292838	C	A	17.8	1.8

EA, effect allele; NEA, non-effect allele; SE, standard error; SNP, single nucleotide polymorphism.

Supplementary Table S3. Drug target variants included in the genetic score and their associations with low-density lipoprotein cholesterol (LDL-C) levels in the Global Lipids Genetics Consortium.

Target	SNP	Chr	Position	EA	NEA	Beta	SE	P value
HMGCR	rs1308220	5	74537301	G	A	0.049	0.006	5.55E-14
HMGCR	rs11741997	5	74560542	G	A	-0.053	0.008	6.14E-09
HMGCR	rs2006760	5	74562029	G	C	0.048	0.002	4.33E-90
HMGCR	rs115845757	5	74563700	A	G	0.063	0.006	6.18E-19
HMGCR	rs147694838	5	74578124	T	A	0.042	0.006	7.62E-11
HMGCR	rs4703665	5	74602898	T	C	-0.034	0.003	2.55E-25
HMGCR	rs35122945	5	74610293	C	A	-0.043	0.004	1.85E-25
HMGCR	rs141642272	5	74615209	C	G	0.080	0.006	1.86E-33
HMGCR	rs111353455	5	74623949	A	G	0.019	0.003	1.28E-08
HMGCR	rs75240579	5	74624484	T	C	-0.048	0.005	1.27E-18
HMGCR	rs112672253	5	74628706	T	A	0.065	0.010	1.06E-08
HMGCR	rs17244939	5	74631096	C	A	-0.057	0.008	4.01E-09
HMGCR	rs115169875	5	74633264	A	G	-0.039	0.006	1.22E-09
HMGCR	rs12916	5	74656539	C	T	0.072	0.002	1.94E-314
HMGCR	rs151264833	5	74660755	G	C	0.036	0.006	1.11E-08
HMGCR	rs62366588	5	74664987	A	C	-0.043	0.004	6.84E-22
HMGCR	rs17562686	5	74668589	T	C	0.056	0.003	6.50E-73
HMGCR	rs16872526	5	74675717	G	T	0.036	0.003	4.86E-25
HMGCR	rs74695562	5	74675951	G	T	-0.039	0.005	9.18E-14
HMGCR	rs144083983	5	74683306	T	C	-0.048	0.004	1.02E-29
HMGCR	rs181668591	5	74689045	T	C	0.070	0.008	1.23E-13
HMGCR	rs114253542	5	74692810	C	T	0.079	0.011	4.47E-11
HMGCR	rs72633966	5	74701662	G	C	0.064	0.003	1.72E-113
HMGCR	rs180755046	5	74703989	A	C	-0.054	0.008	2.11E-08
HMGCR	rs182826525	5	74719210	G	A	0.104	0.009	2.20E-26
HMGCR	rs200823803	5	74753738	C	T	0.060	0.007	3.41E-14
NPC1L1	rs3187907	7	44552209	C	T	0.017	0.002	4.48E-13
NPC1L1	rs10260606	7	44584551	C	G	0.043	0.002	1.44E-71
NPC1L1	rs73107478	7	44596644	C	A	0.040	0.003	4.98E-28
NPC1L1	rs217381	7	44606217	G	C	-0.031	0.002	1.07E-58
NPC1L1	rs79854399	7	44640315	T	C	-0.047	0.006	2.19E-11
NPC1L1	rs2268310	7	44677098	T	C	0.030	0.003	3.87E-24
PCSK9	rs11580527	1	55411580	A	G	-0.186	0.007	4.10E-118
PCSK9	rs1863668	1	55411708	T	G	-0.013	0.002	2.68E-11
PCSK9	rs12043403	1	55431933	C	T	-0.028	0.003	2.79E-17
PCSK9	rs12409233	1	55436599	C	G	-0.025	0.003	5.78E-14
PCSK9	rs890576	1	55452897	G	C	-0.015	0.002	9.57E-11
PCSK9	rs146273942	1	55453841	A	G	-0.049	0.006	1.66E-12

PCSK9	rs145075626	1	55479043	G	C	-0.046	0.007	5.29E-10
PCSK9	rs2479396	1	55482091	G	A	-0.013	0.002	3.92E-09
PCSK9	rs146480899	1	55483477	G	A	-0.048	0.006	8.54E-12
PCSK9	rs77875082	1	55485042	A	G	0.045	0.005	7.20E-15
PCSK9	rs2479393	1	55488369	A	G	0.034	0.002	5.72E-58
PCSK9	rs34232196	1	55489542	T	C	-0.069	0.002	7.76E-215
PCSK9	rs374459115	1	55489968	A	G	0.074	0.008	2.41E-15
PCSK9	rs28775984	1	55491161	C	T	-0.019	0.003	2.19E-08
PCSK9	rs181331606	1	55493672	G	C	0.027	0.004	6.94E-11
PCSK9	rs187758512	1	55493674	G	A	0.026	0.002	3.99E-26
PCSK9	rs72909541	1	55494301	T	C	-0.059	0.004	3.53E-37
PCSK9	rs12739979	1	55496648	T	C	-0.032	0.002	1.56E-36
PCSK9	rs11810371	1	55496861	A	G	-0.046	0.004	2.18E-19
PCSK9	rs2094470	1	55497302	C	T	0.028	0.003	5.54E-14
PCSK9	rs72660548	1	55500978	G	C	0.074	0.006	1.47E-29
PCSK9	rs2479409	1	55504650	G	A	0.048	0.002	1.30E-124
PCSK9	rs11591147	1	55505647	T	G	-0.432	0.006	0.00E+00
PCSK9	rs11206513	1	55507649	C	T	-0.042	0.002	3.51E-97
PCSK9	rs74700387	1	55510015	T	C	-0.043	0.006	4.23E-09
PCSK9	rs7546522	1	55516713	T	C	-0.025	0.002	6.86E-20
PCSK9	rs572512	1	55517344	T	C	0.026	0.002	9.97E-34
PCSK9	rs41294825	1	55519068	T	A	-0.042	0.004	5.98E-20
PCSK9	rs45508296	1	55520547	G	A	-0.069	0.006	1.88E-22
PCSK9	rs150119739	1	55520938	A	G	0.067	0.004	6.48E-41
PCSK9	rs693668	1	55521109	G	A	-0.058	0.002	1.94E-192
PCSK9	rs7525503	1	55522558	T	G	0.065	0.006	8.31E-23
PCSK9	rs11206517	1	55526428	G	T	0.081	0.004	2.93E-62
PCSK9	rs41297885	1	55526840	G	C	-0.038	0.004	1.08E-13
PCSK9	rs28385715	1	55532142	G	T	0.050	0.006	3.07E-13
PCSK9	rs77011887	1	55534271	T	C	0.058	0.007	1.48E-12
PCSK9	rs142116310	1	55536386	A	G	0.057	0.009	1.90E-08
PCSK9	rs10493176	1	55538552	G	T	-0.077	0.003	7.82E-120
PCSK9	rs115465289	1	55580914	A	G	-0.047	0.005	1.19E-15
PCSK9	rs12031153	1	55601339	A	G	0.039	0.004	1.81E-15
PCSK9	rs55817205	1	55609056	A	G	0.069	0.009	1.76E-12
PCSK9	rs1165227	1	55616754	T	C	-0.023	0.002	1.82E-18
LDLR	rs12983316	19	11114352	G	A	0.043	0.002	6.32E-61
LDLR	rs13345127	19	11116266	T	C	0.018	0.003	9.01E-09
LDLR	rs116945003	19	11119233	A	G	0.077	0.011	1.93E-10
LDLR	rs148724334	19	11122019	C	G	0.055	0.007	5.59E-11
LDLR	rs73013166	19	11126160	C	T	-0.127	0.004	5.12E-202
LDLR	rs117210556	19	11126197	C	T	0.038	0.003	1.99E-23
LDLR	rs145033590	19	11135189	A	G	0.067	0.010	3.08E-09

LDLR	rs3786721	19	11146499	T	C	0.046	0.002	1.47E-130
LDLR	rs73013176	19	11147526	C	T	-0.217	0.008	1.31E-123
LDLR	rs144834811	19	11151125	G	A	0.042	0.004	3.55E-17
LDLR	rs144624209	19	11152888	A	G	-0.071	0.004	1.26E-62
LDLR	rs10420325	19	11158047	A	T	0.016	0.002	8.83E-10
LDLR	rs4804564	19	11158055	C	T	-0.047	0.004	1.46E-27
LDLR	rs36049922	19	11169305	C	T	0.043	0.005	8.97E-15
LDLR	rs56315738	19	11175823	T	C	-0.111	0.010	1.21E-21
LDLR	rs73015007	19	11183837	A	G	-0.073	0.002	2.06E-240
LDLR	rs10423733	19	11185919	C	T	-0.122	0.002	0.00E+00
LDLR	rs142274292	19	11191624	T	C	0.106	0.010	5.96E-22
LDLR	rs112159161	19	11192603	T	C	-0.179	0.007	1.76E-106
LDLR	rs146335137	19	11200514	T	C	0.066	0.010	2.04E-09
LDLR	rs17242353	19	11200806	T	C	0.106	0.005	3.22E-72
LDLR	rs17242367	19	11204627	T	C	0.023	0.003	9.49E-09
LDLR	rs17248748	19	11206040	T	C	-0.071	0.007	5.37E-21
LDLR	rs6511721	19	11206575	G	A	0.059	0.002	1.12E-198
LDLR	rs73015030	19	11207516	A	G	-0.134	0.005	6.43E-127
LDLR	rs28493980	19	11212179	G	C	0.054	0.004	6.17E-41
LDLR	rs116886614	19	11221951	G	A	0.067	0.008	1.56E-13
LDLR	rs2738447	19	11227480	A	C	-0.056	0.002	8.61E-190
LDLR	rs41301949	19	11231335	G	A	0.049	0.005	9.80E-18
LDLR	rs147223423	19	11232261	T	A	0.094	0.008	1.37E-23
LDLR	rs17249001	19	11241428	A	G	0.073	0.004	2.98E-68
LDLR	rs3826810	19	11242133	A	G	0.028	0.004	1.42E-09
LDLR	rs5742911	19	11243445	G	A	-0.057	0.002	1.06E-170
LDLR	rs147540853	19	11248104	A	G	-0.082	0.005	6.63E-46
LDLR	rs138059072	19	11250617	T	C	0.091	0.012	2.89E-11
LDLR	rs11557092	19	11257018	T	C	-0.022	0.002	1.48E-24
LDLR	rs141618758	19	11257499	C	T	0.058	0.006	8.50E-17
LDLR	rs62129138	19	11259528	A	G	0.034	0.002	1.06E-41
LDLR	rs140181075	19	11264618	T	C	0.060	0.004	6.88E-34
LDLR	rs189143685	19	11274168	T	C	0.056	0.008	1.38E-09
LDLR	rs4804573	19	11277232	A	G	-0.059	0.002	2.15E-200
LDLR	rs117339792	19	11282298	A	G	-0.081	0.008	1.01E-20
LDLR	rs146576912	19	11283222	T	C	-0.145	0.004	3.23E-198
LDLR	rs4804149	19	11284028	C	T	0.025	0.002	1.45E-28
LDLR	rs379309	19	11284302	C	T	0.027	0.002	1.45E-44
LDLR	rs139198665	19	11284392	T	C	0.069	0.006	1.58E-25
LDLR	rs34824923	19	11291592	A	G	0.029	0.004	2.29E-11
LDLR	rs34243815	19	11324965	T	C	-0.026	0.004	1.23E-10
LDLR	rs4804150	19	11327571	T	C	-0.019	0.002	1.52E-24
LDLR	rs66466742	19	11336444	T	C	-0.045	0.004	1.10E-22

LDLR	rs1433091	19	11336626	A	G	0.030	0.003	3.56E-17
LDLR	rs1560699	19	11337454	T	C	0.058	0.009	1.90E-08

EA, effect allele; HMGCR, 3-hydroxy-3-methylglutaryl-CoA reductase; LDLR, low density lipoprotein receptor; NEA, non-effect allele; NPC1L1, NPC1 like intracellular cholesterol transporter 1; PCSK9, proprotein convertase subtilisin/kexin type 9; SE, standard error; SNP, single nucleotide polymorphism.

Supplementary Table S4. Genetic variants included in low-density lipoprotein cholesterol (LDL-C) genetic score.

SNP	Chr	Position	EA	NEA	Beta	SE	P value
rs1123571	1	2326009	A	G	-0.011	0.002	7.21E-09
rs75907879	1	16170903	T	C	0.020	0.003	1.62E-11
rs10903129	1	25768937	A	G	-0.025	0.002	2.28E-40
rs79778025	1	26449337	G	A	-0.016	0.002	2.47E-09
rs79598313	1	27284913	T	C	0.074	0.005	1.47E-35
rs8681	1	45468606	A	G	-0.013	0.002	3.50E-09
rs2495496	1	55496449	T	A	0.019	0.003	8.93E-10
rs11591147	1	55505647	T	G	-0.432	0.006	0.00E+00
rs693668	1	55521109	G	A	-0.058	0.002	1.94E-192
rs11206517	1	55526428	G	T	0.081	0.004	2.93E-62
rs80098465	1	56323794	A	G	-0.043	0.005	4.32E-16
rs7534572	1	62999675	C	G	-0.040	0.002	5.10E-78
rs7515577	1	93009438	C	A	-0.025	0.002	1.49E-27
rs74896173	1	109167705	C	T	-0.061	0.010	1.96E-08
rs12746961	1	109800321	T	C	-0.128	0.003	0.00E+00
rs604349	1	109839392	A	G	-0.101	0.003	2.07E-222
rs116363925	1	109926599	T	G	0.037	0.004	4.04E-17
rs116056646	1	109938536	G	A	0.036	0.005	2.77E-09
rs267733	1	150958836	G	A	-0.019	0.002	4.31E-14
rs4390169	1	155106054	A	G	0.013	0.002	1.22E-11
rs6682862	1	177938437	A	G	-0.014	0.002	1.03E-08
rs1689801	1	182165484	A	G	0.015	0.002	8.66E-14
rs2296288	1	183072590	T	C	-0.011	0.002	2.82E-09
rs2642438	1	220970028	A	G	-0.025	0.002	1.81E-35
rs553427	1	234852760	C	T	-0.038	0.002	6.09E-91
rs10910522	1	235011725	A	G	-0.011	0.002	6.48E-09
rs3738622	1	235110859	T	G	-0.016	0.002	4.21E-12
rs3820897	2	3642361	T	C	0.016	0.002	6.97E-12
rs35633876	2	20363074	T	G	-0.011	0.002	1.74E-08
rs13392272	2	21217490	T	C	0.075	0.002	0.00E+00
rs72902590	2	21289068	A	G	-0.092	0.004	2.12E-79
rs13396400	2	21502727	G	A	0.031	0.002	1.32E-56
rs11683091	2	21942735	T	C	0.015	0.002	4.65E-12
rs2068834	2	27839539	C	T	0.014	0.002	9.39E-12
rs2374569	2	43885057	T	C	-0.011	0.002	1.47E-08
rs185263492	2	43996767	A	T	0.033	0.002	4.19E-32
rs4299376	2	44072576	G	T	0.065	0.002	9.36E-228
rs6709904	2	44080324	G	A	-0.045	0.003	8.79E-52
rs149233695	2	44839083	T	G	-0.044	0.006	1.48E-10

rs360804	2	62939397	A	G	-0.022	0.002	8.38E-28
rs10193634	2	64933985	C	A	-0.012	0.002	2.25E-10
rs2970902	2	88429183	C	G	0.012	0.002	1.37E-09
rs2465956	2	109065004	C	T	0.019	0.002	3.90E-16
rs150474434	2	118845121	A	G	-0.042	0.003	4.12E-39
rs17050272	2	121306440	A	G	-0.020	0.002	1.90E-24
rs1375131	2	135954797	C	T	0.021	0.002	5.03E-18
rs10184376	2	158442005	T	C	-0.027	0.003	1.22E-14
rs10184673	2	169827796	G	A	0.020	0.002	1.64E-24
rs10932008	2	204048840	G	A	-0.025	0.002	1.31E-22
rs1250259	2	216300482	T	A	-0.017	0.002	3.64E-16
rs887829	2	234668570	T	C	-0.016	0.002	1.20E-15
rs1177809	3	12466490	G	A	-0.024	0.002	3.90E-33
rs6792725	3	24520283	A	G	0.016	0.002	2.52E-14
rs9837622	3	32514647	A	T	-0.035	0.003	7.70E-22
rs71311871	3	58420613	G	A	-0.036	0.003	4.84E-27
rs55921103	3	69810294	G	T	-0.011	0.002	2.34E-08
rs10936349	3	100453859	T	C	0.011	0.002	4.24E-08
rs3732359	3	119536429	G	A	0.014	0.002	1.70E-10
rs12054451	3	122064369	G	T	0.015	0.002	1.13E-12
rs78946096	3	132188163	G	A	-0.041	0.004	5.82E-21
rs9653945	3	142660706	A	G	-0.011	0.002	5.38E-09
rs1584688	3	160267858	C	T	0.013	0.002	2.09E-11
rs56118251	3	171534525	G	A	0.015	0.002	1.16E-09
rs13108218	4	3443931	A	G	0.016	0.002	8.91E-17
rs34707604	4	69491456	C	T	0.030	0.002	4.66E-30
rs28497720	4	100487370	T	C	-0.018	0.002	2.29E-17
rs17617028	4	106064683	A	G	0.015	0.002	4.78E-11
rs793900	4	185253019	T	C	0.011	0.002	1.22E-08
rs77704739	5	52080909	C	T	-0.049	0.004	1.31E-22
rs3010266	5	71965007	A	G	-0.015	0.002	1.32E-09
rs11957820	5	74350090	T	C	0.025	0.003	1.51E-11
rs12916	5	74656539	C	T	0.072	0.002	1.94E-314
rs13189347	5	122828560	C	A	-0.017	0.002	1.55E-18
rs6894249	5	131797547	G	A	0.016	0.002	1.94E-16
rs13161656	5	141916867	C	T	0.022	0.003	8.88E-14
rs12657266	5	156396003	C	T	-0.033	0.002	2.50E-64
rs7746081	6	16126934	A	G	-0.031	0.002	4.94E-56
rs80215559	6	25918225	C	T	-0.058	0.003	1.42E-49
rs71536537	6	32523877	C	T	-0.026	0.004	5.28E-10
rs6689	6	32627700	G	A	0.041	0.002	1.07E-56
rs11754773	6	34577257	G	A	-0.027	0.003	2.33E-18
rs6940814	6	42924932	A	G	0.014	0.002	3.72E-13

rs17665178	6	52435243	G	C	-0.018	0.002	2.97E-18
rs6458949	6	53508265	T	G	0.013	0.002	3.99E-10
rs9496567	6	100602753	A	G	-0.019	0.002	2.10E-17
rs240762	6	100985574	C	T	0.013	0.002	1.23E-11
rs4946713	6	106374015	A	C	-0.011	0.002	1.22E-08
rs1556857	6	116325559	C	T	-0.015	0.002	2.96E-16
rs72971192	6	127088318	C	T	-0.013	0.002	1.80E-08
rs7758845	6	135428537	C	A	-0.025	0.002	8.64E-33
rs75393372	6	139187883	C	T	0.020	0.003	1.58E-08
rs112170089	6	160502019	A	G	0.055	0.008	1.86E-10
rs12208357	6	160543148	T	C	0.059	0.003	2.86E-61
rs146534110	6	160578069	T	G	0.075	0.008	7.28E-16
rs74907759	6	160857193	G	A	-0.059	0.009	1.05E-08
rs117733303	6	160922870	G	A	0.142	0.006	1.35E-86
rs10455872	6	161010118	G	A	0.112	0.003	1.24E-191
rs12055389	6	161091952	T	C	-0.028	0.003	8.15E-13
rs10263252	7	1049949	A	G	-0.021	0.002	1.38E-20
rs55696093	7	21605973	G	A	0.038	0.002	1.25E-62
rs896311	7	25934357	G	A	-0.018	0.002	5.28E-19
rs56001710	7	25983400	A	T	-0.018	0.002	1.94E-13
rs12533280	7	36171953	T	C	0.016	0.002	8.20E-12
rs10260606	7	44584551	C	G	0.043	0.002	1.44E-71
rs42122	7	72841823	A	G	0.015	0.002	9.42E-09
rs6967728	7	97915637	A	G	-0.016	0.002	1.26E-10
rs564449	7	100321138	T	G	0.027	0.003	2.51E-20
rs2911971	8	6607634	C	G	-0.012	0.002	3.48E-09
rs9987289	8	9183358	A	G	-0.057	0.003	2.20E-72
rs6601302	8	9239458	T	G	0.015	0.002	1.90E-12
rs900776	8	21918089	C	A	-0.019	0.002	1.60E-14
rs17526980	8	28950309	T	C	0.030	0.005	3.75E-08
rs28615248	8	55451193	C	T	0.025	0.002	1.98E-25
rs9297994	8	59392324	G	A	0.032	0.002	1.64E-58
rs62509311	8	74907295	T	A	-0.015	0.002	6.67E-13
rs2737252	8	116663898	A	G	-0.022	0.002	1.90E-26
rs28601761	8	126500031	G	C	-0.057	0.002	3.06E-198
rs11787335	8	145044104	T	C	0.024	0.002	1.32E-33
rs3780181	9	2640759	G	A	-0.035	0.003	3.34E-21
rs28498684	9	16900695	A	G	0.013	0.002	3.92E-11
rs12551960	9	19267440	T	C	0.033	0.003	9.38E-21
rs615552	9	22026077	C	T	0.011	0.002	9.53E-09
rs7864568	9	78212428	A	G	-0.016	0.002	1.97E-13
rs7046887	9	78736048	T	C	0.013	0.002	1.64E-12
rs9410207	9	91404799	C	T	-0.021	0.003	2.99E-08

rs2297400	9	107599481	C	T	0.016	0.003	4.90E-08
rs2740488	9	107661742	C	A	-0.021	0.002	1.32E-23
rs6478851	9	131561110	A	G	-0.015	0.002	1.25E-11
rs2519093	9	136141870	T	C	0.073	0.002	1.90E-217
rs11999532	9	139371405	C	G	-0.015	0.002	1.33E-12
rs7903259	10	17259642	G	C	0.017	0.002	8.12E-20
rs10761750	10	65128619	A	G	0.015	0.002	1.56E-15
rs2068888	10	94839642	A	G	-0.015	0.002	2.03E-16
rs61886346	10	96101364	T	C	-0.022	0.003	2.78E-08
rs603424	10	102075479	A	G	0.014	0.002	2.06E-08
rs2792751	10	113940329	T	C	0.023	0.002	3.40E-30
rs60847460	10	113983758	T	C	-0.018	0.002	9.75E-12
rs4751995	10	118397884	A	G	-0.016	0.002	9.54E-18
rs9423289	10	124704695	C	T	-0.022	0.002	1.66E-31
rs12271225	11	5690544	T	A	0.016	0.002	4.36E-09
rs11601507	11	5701074	A	C	0.040	0.003	3.37E-29
rs10832956	11	18645668	T	C	-0.021	0.002	1.75E-24
rs174546	11	61569830	T	C	-0.044	0.002	2.16E-111
rs642803	11	65560620	T	C	0.012	0.002	7.88E-11
rs2072560	11	116661826	T	C	0.044	0.003	5.75E-32
rs45505501	11	118405305	A	G	0.018	0.002	2.25E-11
rs76970536	11	126250680	A	G	0.059	0.003	1.77E-61
rs34019521	12	618790	C	G	0.019	0.002	4.29E-17
rs12320328	12	25408464	G	A	-0.026	0.003	3.17E-14
rs11175540	12	40586295	A	T	0.026	0.003	1.59E-11
rs2251024	12	51103633	T	C	-0.017	0.002	1.10E-17
rs9795910	12	51795623	G	A	-0.012	0.002	4.28E-10
rs11105294	12	89858839	A	G	0.012	0.002	6.31E-10
rs7955221	12	100850750	C	A	-0.012	0.002	2.00E-09
rs3184504	12	111884608	T	C	-0.025	0.002	1.23E-41
rs1169288	12	121416650	C	A	0.035	0.002	6.26E-71
rs11057841	12	125316743	T	C	0.021	0.002	2.55E-14
rs77502095	12	133119022	A	G	0.016	0.002	1.26E-08
rs7327867	13	32968591	G	A	0.019	0.002	8.66E-25
rs207637	13	33085469	G	T	0.011	0.002	2.87E-08
rs9805560	13	50076079	G	A	0.011	0.002	5.64E-09
rs9524538	13	95218192	A	G	0.017	0.002	1.45E-15
rs551473284	13	111038325	T	C	-0.015	0.002	1.83E-10
rs6602909	13	114551993	C	T	0.016	0.002	2.20E-15
rs12016920	13	114639025	C	T	-0.017	0.002	4.97E-12
rs11621792	14	24871926	T	C	0.021	0.002	2.21E-27
rs11846704	14	35186694	T	C	-0.012	0.002	3.93E-09
rs11620731	14	70817141	T	C	-0.025	0.002	4.15E-21

rs13379043	14	74250126	C	T	-0.016	0.002	1.18E-14
rs17776811	14	90066451	A	C	0.011	0.002	2.37E-08
rs28929474	14	94844947	T	C	0.065	0.006	3.45E-20
rs17580	14	94847262	A	T	0.054	0.005	7.31E-26
rs79391862	15	53739426	C	A	-0.065	0.006	3.95E-21
rs11635502	15	63771730	A	G	0.013	0.002	4.76E-10
rs11638576	15	75260387	A	G	0.015	0.002	2.73E-13
rs8041391	15	89102448	T	C	0.015	0.002	4.66E-08
rs12445804	16	11706100	A	G	0.029	0.003	6.13E-16
rs7184567	16	31021078	T	C	0.011	0.002	8.27E-09
rs247617	16	56990716	A	C	-0.035	0.002	3.16E-68
rs7206039	16	71645914	A	C	0.012	0.002	1.30E-09
rs12924886	16	72075593	T	A	0.054	0.002	3.96E-117
rs7202323	16	72217113	G	T	-0.021	0.002	9.11E-21
rs67890964	16	83979317	C	T	-0.019	0.002	3.27E-21
rs55714927	17	7080316	T	C	-0.035	0.002	2.11E-45
rs150688657	17	7505801	A	G	0.021	0.003	9.94E-12
rs704	17	26694861	A	G	0.020	0.002	6.89E-28
rs67777803	17	27323322	T	G	-0.015	0.002	5.40E-09
rs6505220	17	29271319	T	A	-0.014	0.002	1.13E-10
rs72836561	17	41926126	T	C	-0.037	0.005	1.03E-11
rs11870935	17	45732605	G	A	0.025	0.002	1.76E-40
rs2665404	17	57875396	C	T	-0.011	0.002	8.44E-09
rs1801689	17	64210580	C	A	0.095	0.005	1.83E-58
rs17647249	17	65195226	T	C	0.013	0.002	2.68E-12
rs77542162	17	67081278	G	A	0.169	0.006	3.34E-125
rs72631343	17	67191270	G	C	-0.041	0.002	1.32E-50
rs2125345	17	73782191	C	T	-0.018	0.002	1.31E-18
rs12451056	17	76396188	T	C	-0.020	0.002	8.72E-15
rs77960347	18	47109955	G	A	0.064	0.007	2.63E-14
rs4939883	18	47167214	T	C	-0.016	0.002	2.97E-11
rs141453190	19	10453122	T	C	-0.033	0.005	2.40E-08
rs76127343	19	11065457	T	C	0.038	0.005	1.58E-12
rs10423733	19	11185919	C	T	-0.122	0.002	0.00E+00
rs35878749	19	11229765	A	G	-0.043	0.002	3.17E-106
rs7250652	19	11302606	G	A	0.025	0.002	1.21E-39
rs58542926	19	19379549	T	C	-0.099	0.003	3.59E-174
rs7937	19	41302706	C	T	-0.011	0.002	6.79E-09
rs73048351	19	45160086	A	C	-0.260	0.009	1.39E-144
rs146842409	19	45193373	A	G	0.123	0.015	2.67E-13
rs11673631	19	45225423	C	G	0.052	0.004	7.06E-25
rs1551891	19	45231821	A	G	-0.164	0.003	0.00E+00
rs10406338	19	45326217	C	T	0.083	0.002	0.00E+00

rs375972689	19	45360488	G	T	0.182	0.008	3.40E-89
rs10420434	19	45451190	A	G	-0.040	0.004	3.32E-17
rs7247937	19	45827863	C	G	-0.016	0.002	1.13E-14
rs35313547	19	58352806	C	T	-0.015	0.002	7.82E-09
rs34503352	19	58651296	A	G	-0.029	0.002	3.32E-29
rs73066228	19	59013196	G	A	0.017	0.002	8.45E-12
rs6107650	20	5526113	G	A	-0.011	0.002	2.00E-08
rs438568	20	12958687	A	G	-0.013	0.002	3.35E-12
rs969075	20	17792323	T	C	-0.015	0.002	4.07E-14
rs2618566	20	17844684	G	T	0.040	0.002	1.55E-86
rs61016611	20	34212151	A	G	-0.032	0.003	1.06E-25
rs1883711	20	39179822	C	G	0.128	0.005	4.34E-113
rs6093446	20	39780932	A	G	0.024	0.002	3.96E-30
rs1800961	20	43042364	T	C	-0.049	0.004	5.76E-22
rs2295027	20	47582884	A	G	0.012	0.002	6.78E-09
rs35046559	20	62344480	G	A	0.012	0.002	2.85E-08
rs8121509	20	62712053	C	T	-0.014	0.002	4.16E-12
rs60417583	20	62890877	T	G	0.015	0.002	4.78E-08
rs16988435	21	33058571	T	C	0.032	0.004	8.42E-14
rs452377	21	40537183	C	T	-0.012	0.002	1.64E-09
rs5746498	22	18435794	C	T	0.014	0.002	1.42E-09
rs5752963	22	30203833	A	G	0.029	0.004	3.40E-09
rs4465	22	35708790	C	T	0.014	0.002	1.78E-13
rs138335	22	41227086	C	G	0.013	0.002	3.04E-10
rs3747207	22	44324855	A	G	-0.014	0.002	3.33E-10
rs13057311	22	50878196	A	G	-0.012	0.002	2.23E-08

EA, effect allele; NEA, non-effect allele; SE, standard error; SNP, single nucleotide polymorphism.

Supplementary Table S5. Detailed information about genetic instruments used to proxy body mass index (BMI), systolic blood pressure (SBP), and lifestyle factors.

**Modifiable factor**	**IVs**	**Unit**	**Participants**	**PubMed ID**
Body mass index (BMI)	62	SD	339,224 individuals of multi-ancestries	25673413
Systolic blood pressure (SBP)	455	10 mmHg	Up to 1,006,863 European-descent individuals	30224653
Smoking initiation	248	SD in prevalence of smoking initiation	1 ,232,091 European-descent individuals	30643251
Alcohol drinking	98	SD increase of log-transformed alcoholic drinks/week	941,280 European-descent individuals	30643251
Moderate-to-vigorous intensity physical activity	16	More than 30 min per week	606,820 individuals of multi-ancestries	36071172

IVs, instrumental variables; SD, standard deviation.

Supplementary Table S6. Genetic instruments used to calculate genetic scores for body mass index (BMI), systolic blood pressure (SBP), and lifestyle factors.

Modifiable factor	SNP	Chr	Position	EA	NEA	Beta	SE	P value
BMI	rs657452	1	49589847	A	G	0.023	0.003	5.48E-13
BMI	rs3101336	1	72751185	C	T	0.033	0.003	2.66E-26
BMI	rs12401738	1	78446761	A	G	0.021	0.003	1.15E-10
BMI	rs11165643	1	96924097	T	C	0.022	0.003	2.07E-12
BMI	rs17024393	1	110154688	C	T	0.066	0.009	7.03E-14
BMI	rs543874	1	177889480	G	A	0.048	0.004	2.62E-35
BMI	rs2820292	1	201784287	C	A	0.020	0.003	1.83E-10
BMI	rs13021737	2	632348	G	A	0.060	0.004	1.11E-50
BMI	rs10182181	2	25150296	G	A	0.031	0.003	8.78E-24
BMI	rs1016287	2	59305625	T	C	0.023	0.003	2.25E-11
BMI	rs2121279	2	143043285	T	C	0.025	0.004	2.31E-08
BMI	rs1528435	2	181550962	T	C	0.018	0.003	1.20E-08
BMI	rs7599312	2	213413231	G	A	0.022	0.003	1.17E-10
BMI	rs6804842	3	25106437	G	A	0.019	0.003	2.48E-09
BMI	rs2365389	3	61236462	C	T	0.020	0.003	1.63E-10
BMI	rs3849570	3	81792112	A	C	0.019	0.003	2.60E-08
BMI	rs13078960	3	85807590	G	T	0.030	0.004	1.74E-14
BMI	rs16851483	3	141275436	T	G	0.048	0.008	3.55E-10
BMI	rs1516725	3	185824004	C	T	0.045	0.005	1.89E-22
BMI	rs10938397	4	45182527	G	A	0.040	0.003	3.21E-38
BMI	rs17001654	4	77129568	G	C	0.031	0.005	7.76E-09
BMI	rs13107325	4	103188709	T	C	0.048	0.007	1.83E-12
BMI	rs11727676	4	145659064	T	C	0.036	0.006	2.55E-08
BMI	rs2112347	5	75015242	T	G	0.026	0.003	6.19E-17
BMI	rs205262	6	34563164	G	A	0.022	0.004	1.75E-10
BMI	rs2207139	6	50845490	G	A	0.045	0.004	4.13E-29
BMI	rs9400239	6	108977663	C	T	0.019	0.003	1.61E-08
BMI	rs13191362	6	163033350	A	G	0.028	0.005	7.34E-09
BMI	rs1167827	7	75163169	G	A	0.020	0.003	6.33E-10
BMI	rs2245368	7	76608143	C	T	0.032	0.006	3.19E-08
BMI	rs17405819	8	76806584	T	C	0.022	0.003	2.07E-11
BMI	rs2033732	8	85079709	C	T	0.019	0.004	4.89E-08
BMI	rs4740619	9	15634326	T	C	0.018	0.003	4.56E-09
BMI	rs10968576	9	28414339	G	A	0.025	0.003	6.61E-14
BMI	rs6477694	9	111932342	C	T	0.017	0.003	2.67E-08
BMI	rs1928295	9	120378483	T	C	0.019	0.003	7.91E-10
BMI	rs10733682	9	129460914	A	G	0.017	0.003	1.83E-08

BMI	rs7899106	10	87410904	G	A	0.040	0.007	2.96E-08
BMI	rs17094222	10	102395440	C	T	0.025	0.004	5.94E-11
BMI	rs7903146	10	114758349	C	T	0.023	0.003	1.11E-11
BMI	rs4256980	11	8673939	G	C	0.021	0.003	2.90E-11
BMI	rs11030104	11	27684517	A	G	0.041	0.004	5.56E-28
BMI	rs2176598	11	43864278	T	C	0.020	0.004	2.97E-08
BMI	rs3817334	11	47650993	T	C	0.026	0.003	5.15E-17
BMI	rs12286929	11	115022404	G	A	0.022	0.003	1.31E-12
BMI	rs7138803	12	50247468	A	G	0.032	0.003	8.15E-24
BMI	rs11057405	12	122781897	G	A	0.031	0.006	2.02E-08
BMI	rs12429545	13	54102206	A	G	0.033	0.005	1.09E-12
BMI	rs10132280	14	25928179	C	A	0.023	0.003	1.14E-11
BMI	rs7141420	14	79899454	T	C	0.024	0.003	1.23E-14
BMI	rs3736485	15	51748610	A	G	0.018	0.003	7.41E-09
BMI	rs16951275	15	68077168	T	C	0.031	0.004	1.91E-17
BMI	rs758747	16	3627358	T	C	0.023	0.004	7.47E-10
BMI	rs3888190	16	28889486	A	C	0.031	0.003	3.14E-23
BMI	rs1558902	16	53803574	A	T	0.082	0.003	7.51E-153
BMI	rs1000940	17	5283252	G	A	0.019	0.003	1.28E-08
BMI	rs12940622	17	78615571	G	A	0.018	0.003	2.49E-09
BMI	rs1808579	18	21104888	C	T	0.017	0.003	4.17E-08
BMI	rs6567160	18	57829135	C	T	0.056	0.004	3.93E-53
BMI	rs17724992	19	18454825	A	G	0.019	0.004	3.42E-08
BMI	rs29941	19	34309532	G	A	0.018	0.003	2.41E-08
BMI	rs2287019	19	46202172	C	T	0.036	0.004	4.59E-18
SBP	rs7796	1	1684169	C	G	0.339	0.031	5.00E-27
SBP	rs263532	1	2164116	T	C	0.180	0.031	4.72E-09
SBP	rs2493296	1	3327032	T	C	0.418	0.044	3.14E-21
SBP	rs2232460	1	6659505	A	G	-0.217	0.032	1.10E-11
SBP	rs488834	1	10767902	T	C	-0.380	0.037	2.35E-25
SBP	rs6699618	1	11881441	C	G	0.912	0.041	1.68E-109
SBP	rs75461554	1	15810172	T	C	-0.302	0.038	1.18E-15
SBP	rs1889785	1	16348729	A	G	0.178	0.030	4.35E-09
SBP	rs404100	1	25366987	T	C	0.194	0.030	1.68E-10
SBP	rs34079867	1	27407850	T	C	0.199	0.035	1.78E-08
SBP	rs4908348	1	28706949	T	G	0.237	0.033	8.07E-13
SBP	rs11210029	1	41865293	A	G	-0.203	0.031	8.92E-11
SBP	rs1408945	1	42364877	T	G	-0.320	0.030	8.33E-26
SBP	rs1209384	1	43765089	A	G	0.256	0.031	2.85E-16
SBP	rs778124	1	56606206	A	G	0.297	0.031	1.45E-21
SBP	rs61772592	1	56979681	A	G	-0.318	0.046	2.86E-12
SBP	rs12063372	1	59621911	A	G	0.199	0.032	3.86E-10
SBP	rs12136922	1	67007389	A	G	0.203	0.030	2.69E-11

SBP	rs658780	1	78555928	T	G	-0.203	0.035	5.29E-09
SBP	rs786923	1	89242954	T	C	-0.308	0.031	2.83E-23
SBP	rs7514579	1	94051350	A	C	0.224	0.036	5.45E-10
SBP	rs10776752	1	113044328	T	G	0.821	0.058	4.61E-46
SBP	rs59980837	1	115827266	T	G	1.100	0.116	3.32E-21
SBP	rs11585169	1	150572037	A	T	0.180	0.031	5.34E-09
SBP	rs76719272	1	156129796	T	C	-0.274	0.046	2.97E-09
SBP	rs12731646	1	169090660	T	C	-0.189	0.031	7.21E-10
SBP	rs1043069	1	180859368	T	G	0.234	0.031	5.26E-14
SBP	rs4651224	1	184585182	T	C	0.199	0.031	9.00E-11
SBP	rs12042924	1	197297417	T	C	-0.181	0.030	2.62E-09
SBP	rs11120093	1	207211326	T	C	-0.179	0.031	5.13E-09
SBP	rs2724377	1	207974818	A	G	0.194	0.030	1.29E-10
SBP	rs7555285	1	209970355	C	G	0.229	0.038	1.05E-09
SBP	rs68085857	1	217737629	T	C	0.274	0.036	1.68E-14
SBP	rs4595370	1	221298122	A	G	-0.209	0.033	1.73E-10
SBP	rs1745417	1	228204279	T	C	0.287	0.030	1.59E-21
SBP	rs699	1	230845794	A	G	-0.375	0.031	5.59E-34
SBP	rs1565440	1	243387788	A	G	0.175	0.031	1.94E-08
SBP	rs4926499	1	249155909	C	G	0.297	0.044	1.33E-11
SBP	rs17760259	2	19744462	T	C	-0.265	0.030	2.25E-18
SBP	rs2384063	2	25187115	T	C	0.327	0.036	6.33E-20
SBP	rs1275988	2	26914364	T	C	-0.541	0.031	4.42E-69
SBP	rs13420463	2	37517566	A	G	0.314	0.036	2.72E-18
SBP	rs4952609	2	40555733	A	G	0.212	0.035	9.60E-10
SBP	rs115262049	2	43196694	A	T	0.589	0.055	1.29E-26
SBP	rs12464602	2	43397614	A	G	-0.244	0.032	1.02E-14
SBP	rs13016772	2	55779476	T	C	0.252	0.036	1.23E-12
SBP	rs2249105	2	65287896	A	G	0.293	0.031	7.63E-21
SBP	rs10188003	2	66773469	T	C	0.188	0.031	8.80E-10
SBP	rs6731373	2	68503044	A	G	0.191	0.033	4.18E-09
SBP	rs6732123	2	69534650	C	G	-0.174	0.031	1.52E-08
SBP	rs4577304	2	73403040	T	C	-0.177	0.030	4.99E-09
SBP	rs72847885	2	86326717	A	G	0.241	0.032	3.08E-14
SBP	rs10207726	2	112744260	T	C	-0.214	0.033	8.06E-11
SBP	rs6737318	2	114083120	A	G	0.235	0.036	1.13E-10
SBP	rs2580350	2	121996007	A	G	0.177	0.031	8.39E-09
SBP	rs17257081	2	135630498	A	G	0.227	0.039	6.35E-09
SBP	rs55944332	2	145726621	A	G	-0.261	0.036	1.79E-13
SBP	rs62170470	2	146989797	T	C	0.197	0.032	7.69E-10
SBP	rs62187653	2	162469128	T	C	0.329	0.051	1.23E-10
SBP	rs4667454	2	164867726	A	G	0.264	0.032	2.63E-16
SBP	rs73029563	2	165008166	C	G	-0.514	0.030	4.20E-64

SBP	rs10048760	2	174977976	T	G	-0.186	0.030	6.56E-10
SBP	rs71421551	2	177053298	C	G	0.237	0.033	1.05E-12
SBP	rs17610485	2	177540601	A	T	0.174	0.030	1.06E-08
SBP	rs4894132	2	180738654	T	C	0.247	0.034	5.51E-13
SBP	rs12473915	2	182987704	A	G	-0.295	0.038	3.42E-15
SBP	rs13412750	2	191634958	A	G	-0.289	0.034	2.33E-17
SBP	rs12693982	2	204085635	T	C	0.258	0.031	7.49E-17
SBP	rs3845811	2	208521512	C	G	-0.294	0.031	1.88E-21
SBP	rs12694277	2	213188795	T	C	-0.202	0.034	1.80E-09
SBP	rs2161967	2	218680529	T	G	0.284	0.031	2.87E-20
SBP	rs3828282	2	218779144	C	G	0.186	0.032	5.29E-09
SBP	rs10804330	2	227185749	T	C	0.235	0.031	1.62E-14
SBP	rs1044822	2	230629138	T	C	-0.248	0.042	5.16E-09
SBP	rs3754944	2	231279616	A	C	0.177	0.031	9.30E-09
SBP	rs139354822	2	242344695	T	C	0.612	0.098	3.51E-10
SBP	rs9848170	3	11495983	C	G	0.323	0.031	7.01E-26
SBP	rs11925504	3	14943965	A	G	-0.290	0.031	1.78E-21
SBP	rs189267552	3	20073193	A	T	-0.866	0.139	4.55E-10
SBP	rs2643826	3	27562988	T	C	0.447	0.031	1.74E-48
SBP	rs68115553	3	27704702	A	G	-0.645	0.114	1.74E-08
SBP	rs743395	3	37598382	T	C	0.260	0.032	2.55E-16
SBP	rs6788984	3	41107173	A	G	0.300	0.043	3.81E-12
SBP	rs1052501	3	41925398	T	C	0.226	0.041	4.14E-08
SBP	rs6771917	3	48108442	T	C	-0.379	0.036	1.39E-26
SBP	rs7615099	3	53143901	A	G	0.189	0.032	3.90E-09
SBP	rs6445583	3	53562894	A	G	0.277	0.035	1.90E-15
SBP	rs3772219	3	56771251	A	C	0.273	0.032	3.10E-17
SBP	rs7618284	3	66422246	C	G	-0.189	0.033	1.10E-08
SBP	rs4499560	3	70920485	A	T	-0.220	0.033	1.46E-11
SBP	rs1375564	3	85656311	T	C	0.258	0.032	2.84E-16
SBP	rs12637573	3	121682388	A	G	-0.173	0.030	9.95E-09
SBP	rs6438857	3	124557643	T	C	0.274	0.031	3.13E-19
SBP	rs9880098	3	133949366	A	G	0.308	0.031	1.59E-23
SBP	rs1199330	3	138101529	A	G	-0.265	0.047	1.65E-08
SBP	rs9876694	3	141152017	T	C	0.471	0.065	4.64E-13
SBP	rs4408839	3	153729768	A	G	-0.230	0.035	2.43E-11
SBP	rs79539362	3	154680449	T	C	0.400	0.050	2.09E-15
SBP	rs17684859	3	158213841	T	C	-0.224	0.034	4.24E-11
SBP	rs3980686	3	168697602	T	G	-0.500	0.049	1.03E-24
SBP	rs1290784	3	169096900	T	C	0.412	0.030	2.97E-42
SBP	rs2111557	3	169325621	T	C	0.176	0.030	5.22E-09
SBP	rs4955575	3	169534538	A	C	0.216	0.035	5.63E-10
SBP	rs262986	3	183435713	A	G	-0.237	0.031	7.67E-15

SBP	rs13091418	3	185329756	C	G	-0.223	0.033	6.15E-12
SBP	rs9869437	3	196228360	A	C	-0.200	0.032	3.22E-10
SBP	rs34535756	4	2246927	T	C	0.478	0.079	1.18E-09
SBP	rs1290933	4	2668217	A	C	-0.285	0.033	3.17E-18
SBP	rs2498323	4	3451109	A	G	0.317	0.052	8.52E-10
SBP	rs2610990	4	18008232	A	G	-0.290	0.034	2.86E-17
SBP	rs55924432	4	26812737	T	C	0.265	0.032	5.70E-17
SBP	rs2291434	4	38387244	T	G	-0.262	0.030	5.10E-18
SBP	rs12511987	4	46595623	T	G	-0.233	0.040	5.39E-09
SBP	rs62309747	4	48713862	A	G	-0.224	0.030	1.59E-13
SBP	rs60991988	4	54801228	T	G	0.379	0.050	2.82E-14
SBP	rs13107261	4	63768826	A	G	-0.178	0.031	1.57E-08
SBP	rs5020545	4	77414988	T	C	-0.218	0.031	9.71E-13
SBP	rs12509595	4	81182554	T	C	-0.837	0.033	2.55E-138
SBP	rs6823199	4	83925895	T	C	0.209	0.035	1.72E-09
SBP	rs17010957	4	86719165	T	C	-0.534	0.043	1.78E-35
SBP	rs13149209	4	89750668	T	C	0.281	0.037	1.97E-14
SBP	rs13107325	4	103188709	T	C	-0.909	0.059	4.22E-53
SBP	rs11097909	4	106911321	T	C	-0.363	0.043	3.35E-17
SBP	rs1493132	4	108861082	T	C	-0.177	0.032	2.73E-08
SBP	rs1814951	4	111408718	A	G	-0.323	0.047	3.91E-12
SBP	rs4834792	4	120555696	A	T	0.197	0.030	7.24E-11
SBP	rs7439567	4	138464842	T	C	0.254	0.031	2.31E-16
SBP	rs72719160	4	144051276	A	T	-0.224	0.032	4.34E-12
SBP	rs2353940	4	145740898	T	C	-0.208	0.036	6.85E-09
SBP	rs73855810	4	148383424	A	G	0.273	0.043	3.04E-10
SBP	rs7683728	4	156402654	T	C	-0.365	0.030	2.43E-33
SBP	rs12643599	4	156639846	A	G	0.313	0.031	1.23E-23
SBP	rs17035181	4	157678511	T	G	0.307	0.043	7.61E-13
SBP	rs869396	4	169688000	A	C	-0.212	0.031	4.12E-12
SBP	rs4957026	5	361148	A	G	0.198	0.032	8.12E-10
SBP	rs10069690	5	1279790	T	C	0.310	0.037	4.47E-17
SBP	rs7725413	5	15695987	T	C	-0.199	0.036	3.07E-08
SBP	rs12656497	5	32831939	T	C	-0.638	0.031	7.14E-96
SBP	rs10941043	5	33194751	T	G	-0.259	0.033	6.42E-15
SBP	rs2113077	5	50799442	A	G	0.210	0.031	6.09E-12
SBP	rs1694068	5	53283630	A	T	0.266	0.031	1.18E-17
SBP	rs10043077	5	55692939	T	C	-0.193	0.032	2.52E-09
SBP	rs34496659	5	61798934	A	G	0.455	0.062	1.54E-13
SBP	rs6870654	5	63831964	T	C	0.214	0.035	7.58E-10
SBP	rs4286632	5	66291370	A	G	0.211	0.034	7.64E-10
SBP	rs7703560	5	67678506	A	G	-0.225	0.033	1.51E-11
SBP	rs246973	5	68007803	T	C	0.248	0.034	1.45E-13

SBP	rs6452769	5	87389027	A	G	-0.314	0.038	7.82E-17
SBP	rs76443575	5	96211594	C	G	-0.523	0.082	1.40E-10
SBP	rs1871190	5	97953719	T	G	0.195	0.032	1.66E-09
SBP	rs11241313	5	114428167	T	C	-0.207	0.033	2.23E-10
SBP	rs1624823	5	122475438	A	G	0.337	0.031	4.26E-27
SBP	rs9327297	5	122835051	C	G	0.275	0.032	8.07E-18
SBP	rs758179	5	127354424	C	G	-0.209	0.037	1.19E-08
SBP	rs6892983	5	127845030	A	C	0.343	0.031	7.11E-29
SBP	rs702395	5	140086677	T	C	0.232	0.031	3.24E-14
SBP	rs2913920	5	141726983	T	C	0.242	0.036	1.62E-11
SBP	rs1957563	5	157474590	T	C	0.363	0.034	2.32E-26
SBP	rs11960210	5	157817634	T	C	0.473	0.031	1.25E-51
SBP	rs13358657	5	157938070	A	G	-0.388	0.045	2.95E-18
SBP	rs3860770	5	173301427	A	G	-0.266	0.033	1.20E-15
SBP	rs12153395	5	179411477	A	G	-0.330	0.049	1.07E-11
SBP	rs2745599	6	1613686	A	G	0.216	0.032	8.96E-12
SBP	rs1575290	6	7715689	T	C	0.197	0.030	5.59E-11
SBP	rs1630736	6	12295987	T	C	-0.171	0.031	3.52E-08
SBP	rs9349379	6	12903957	A	G	0.266	0.031	1.31E-17
SBP	rs9368222	6	20686996	A	C	0.228	0.034	1.84E-11
SBP	rs2655445	6	22377623	A	G	-0.202	0.031	9.58E-11
SBP	rs79782817	6	25882678	T	G	0.532	0.050	1.43E-26
SBP	rs116025100	6	30935290	A	G	0.537	0.085	2.86E-10
SBP	rs2753960	6	31762844	T	G	0.447	0.031	2.66E-47
SBP	rs2815063	6	39262535	A	C	0.276	0.046	1.76E-09
SBP	rs7763558	6	43349215	A	G	0.336	0.032	1.17E-25
SBP	rs11967262	6	43760327	C	G	-0.172	0.031	3.43E-08
SBP	rs78648104	6	50683009	T	C	-0.429	0.054	2.37E-15
SBP	rs1984195	6	79657391	A	G	0.241	0.030	1.77E-15
SBP	rs9361836	6	82235408	T	C	0.220	0.032	1.25E-11
SBP	rs6921291	6	97066242	T	C	0.358	0.039	1.58E-20
SBP	rs9486916	6	109013930	T	C	0.266	0.039	5.42E-12
SBP	rs961764	6	117522156	C	G	-0.191	0.031	3.75E-10
SBP	rs10782230	6	126228512	A	G	0.211	0.030	2.91E-12
SBP	rs9401913	6	127159982	A	G	0.520	0.031	3.66E-65
SBP	rs2327429	6	134209837	T	C	0.200	0.034	3.16E-09
SBP	rs8180684	6	143200936	T	C	0.213	0.034	1.80E-10
SBP	rs7765526	6	147713764	A	G	0.201	0.031	5.88E-11
SBP	rs17080102	6	151004770	C	G	-0.809	0.059	3.52E-42
SBP	rs1293969	6	151959945	T	C	-0.199	0.035	1.03E-08
SBP	rs509833	6	159711515	A	G	0.329	0.044	7.08E-14
SBP	rs12661036	6	163737476	T	C	-0.210	0.037	1.82E-08
SBP	rs7744902	6	166176722	A	G	-0.409	0.059	5.64E-12

SBP	rs6959688	7	1966831	A	G	-0.234	0.031	4.22E-14
SBP	rs10282122	7	2529623	T	C	-0.302	0.033	2.46E-20
SBP	rs73049928	7	4669949	A	G	-0.238	0.039	1.20E-09
SBP	rs3807925	7	18543250	A	G	-0.186	0.032	5.39E-09
SBP	rs28688791	7	19039605	T	C	-0.322	0.038	2.34E-17
SBP	rs112509803	7	24735004	C	G	-0.264	0.048	3.18E-08
SBP	rs3735533	7	27245893	T	C	-0.910	0.058	5.29E-56
SBP	rs6961048	7	27328187	C	G	-0.530	0.050	1.43E-26
SBP	rs977184	7	28650761	T	C	-0.184	0.031	4.86E-09
SBP	rs11977526	7	46008110	A	G	-0.321	0.031	6.62E-25
SBP	rs12668436	7	47548893	T	C	-0.215	0.035	7.88E-10
SBP	rs848445	7	77572461	T	C	-0.203	0.034	2.28E-09
SBP	rs42377	7	92243672	A	G	-0.315	0.033	1.69E-21
SBP	rs2392929	7	106414069	T	G	-0.751	0.038	1.96E-87
SBP	rs34072724	7	130432469	A	G	-0.242	0.030	1.37E-15
SBP	rs35680304	7	130973495	T	C	0.269	0.031	3.76E-18
SBP	rs75672964	7	131321010	T	C	0.589	0.084	2.35E-12
SBP	rs73727605	7	149474622	A	G	0.362	0.062	6.60E-09
SBP	rs3918226	7	150690176	T	C	0.664	0.058	8.46E-31
SBP	rs10224210	7	151413194	T	C	-0.383	0.034	1.60E-29
SBP	rs1870735	7	155744303	C	G	0.206	0.031	3.61E-11
SBP	rs71499040	8	1711918	C	G	0.222	0.034	5.63E-11
SBP	rs1821002	8	10640065	C	G	0.379	0.031	5.19E-35
SBP	rs10866828	8	23401534	T	C	0.248	0.036	3.19E-12
SBP	rs7821832	8	25889446	T	G	0.422	0.035	6.67E-34
SBP	rs77375686	8	26043622	A	G	-0.347	0.049	8.38E-13
SBP	rs1906672	8	38130025	A	G	0.297	0.036	1.20E-16
SBP	rs4873492	8	51947549	T	C	0.343	0.040	1.61E-17
SBP	rs2354862	8	64501744	A	C	0.251	0.032	2.42E-15
SBP	rs13253358	8	68920135	T	C	0.213	0.033	1.13E-10
SBP	rs2126474	8	76878957	T	G	-0.260	0.031	1.87E-17
SBP	rs9918879	8	77681093	T	G	-0.298	0.050	2.28E-09
SBP	rs148401029	8	81386066	A	C	-0.462	0.085	4.97E-08
SBP	rs10091532	8	82853793	A	C	-0.207	0.031	1.33E-11
SBP	rs843093	8	92528310	A	G	-0.209	0.034	6.95E-10
SBP	rs2613203	8	95253197	A	T	-0.268	0.039	5.81E-12
SBP	rs79069610	8	105921209	T	C	-0.401	0.073	3.68E-08
SBP	rs35783704	8	105966258	A	G	-0.462	0.051	8.81E-20
SBP	rs7830607	8	110097287	A	G	-0.206	0.033	3.09E-10
SBP	rs2470004	8	120358445	T	C	-0.345	0.039	1.28E-18
SBP	rs4598218	8	129483956	T	C	0.191	0.031	1.00E-09
SBP	rs7012866	8	135616959	T	G	-0.233	0.030	1.21E-14
SBP	rs4440615	8	141057641	A	G	-0.220	0.031	1.87E-12

SBP	rs4961293	8	141812374	T	C	0.227	0.030	7.35E-14
SBP	rs7463212	8	143991858	A	T	-0.275	0.031	1.81E-19
SBP	rs60191654	9	753648	A	G	-0.238	0.039	5.88E-10
SBP	rs927315	9	4117713	T	C	0.169	0.030	2.44E-08
SBP	rs1332813	9	9350706	T	C	0.220	0.031	2.32E-12
SBP	rs9886665	9	22942770	T	C	0.205	0.034	2.47E-09
SBP	rs4553000	9	34223553	T	C	-0.204	0.030	1.09E-11
SBP	rs76452347	9	35906471	T	C	-0.297	0.040	7.13E-14
SBP	rs10746963	9	77238558	A	G	-0.218	0.039	2.05E-08
SBP	rs7045409	9	95201540	A	T	-0.186	0.031	2.55E-09
SBP	rs10980408	9	113249071	T	C	-0.761	0.083	3.83E-20
SBP	rs2900568	9	116689758	T	C	-0.189	0.030	2.96E-10
SBP	rs34025993	9	123516572	A	G	0.223	0.031	4.71E-13
SBP	rs7854147	9	125863350	A	G	0.306	0.046	3.29E-11
SBP	rs13289468	9	128180332	A	C	0.249	0.031	3.93E-16
SBP	rs6271	9	136522274	T	C	-0.555	0.061	1.18E-19
SBP	rs11145807	9	139520789	A	G	0.214	0.032	3.54E-11
SBP	rs11252324	10	4124568	T	G	-0.416	0.057	3.61E-13
SBP	rs1623474	10	18471794	T	C	0.383	0.032	7.66E-33
SBP	rs12258967	10	18727959	C	G	0.633	0.034	1.08E-78
SBP	rs3802517	10	28233469	A	T	0.253	0.030	4.65E-17
SBP	rs12264186	10	32289986	T	C	0.214	0.039	3.58E-08
SBP	rs4948643	10	45379759	T	C	0.226	0.034	2.40E-11
SBP	rs34130368	10	48411796	T	G	-0.302	0.050	1.28E-09
SBP	rs4245599	10	60365755	A	G	-0.179	0.031	4.04E-09
SBP	rs57946343	10	63499951	T	C	0.716	0.043	2.10E-63
SBP	rs2236295	10	64564892	T	G	-0.303	0.031	1.05E-22
SBP	rs2177843	10	75409877	T	C	0.439	0.043	2.80E-24
SBP	rs10749572	10	82136664	T	G	-0.203	0.030	1.88E-11
SBP	rs111866816	10	94441507	T	C	0.357	0.060	2.29E-09
SBP	rs2689690	10	95899706	T	C	-0.270	0.032	1.15E-17
SBP	rs2274224	10	96039597	C	G	-0.452	0.030	5.99E-50
SBP	rs1006545	10	102553647	T	G	0.685	0.048	3.50E-46
SBP	rs11191580	10	104906211	T	C	1.100	0.055	7.74E-89
SBP	rs117464403	10	107158054	A	G	0.864	0.120	5.80E-13
SBP	rs12255372	10	114808902	T	G	0.236	0.034	1.94E-12
SBP	rs1801253	10	115805056	C	G	0.463	0.034	2.84E-41
SBP	rs72842207	10	121433675	T	C	-0.203	0.037	3.14E-08
SBP	rs11592107	10	122968964	A	G	0.302	0.033	1.55E-20
SBP	rs7093894	10	124234880	A	C	0.236	0.043	3.16E-08
SBP	rs7912283	10	133773019	A	G	-0.214	0.032	2.94E-11
SBP	rs1133400	10	134459388	A	G	-0.298	0.038	2.53E-15
SBP	rs569550	11	1887068	T	G	-0.577	0.032	1.33E-73

SBP	rs74048190	11	2114221	T	C	-0.440	0.076	6.07E-09
SBP	rs360153	11	9762274	T	C	-0.345	0.031	1.73E-29
SBP	rs2014408	11	16365282	T	C	0.517	0.037	1.26E-43
SBP	rs7926335	11	16917869	T	C	0.314	0.034	2.52E-20
SBP	rs17762	11	22492454	A	G	0.412	0.057	5.60E-13
SBP	rs1382472	11	27273967	A	G	-0.192	0.031	4.47E-10
SBP	rs871004	11	28512458	A	G	0.234	0.032	1.65E-13
SBP	rs10501122	11	30192151	T	C	0.192	0.032	1.18E-09
SBP	rs11604310	11	45351420	T	C	-0.278	0.041	1.46E-11
SBP	rs7107356	11	47676170	A	G	-0.460	0.030	1.63E-52
SBP	rs2904315	11	48109948	A	G	-0.208	0.033	1.58E-10
SBP	rs4427587	11	58436648	T	C	0.206	0.031	4.28E-11
SBP	rs7125196	11	61272565	T	C	0.442	0.047	7.31E-21
SBP	rs2306363	11	65405600	T	G	-0.436	0.038	5.24E-31
SBP	rs7395791	11	69262916	A	G	-0.216	0.031	2.19E-12
SBP	rs10501410	11	72088806	A	G	0.412	0.061	1.10E-11
SBP	rs7927515	11	76125330	A	C	0.227	0.032	1.05E-12
SBP	rs2289124	11	89224477	A	G	-0.308	0.042	1.14E-13
SBP	rs604723	11	100610546	T	C	-0.655	0.034	2.55E-83
SBP	rs629864	11	100699139	T	C	-0.187	0.032	4.69E-09
SBP	rs7926110	11	107086143	T	G	0.260	0.032	5.71E-16
SBP	rs236916	11	117089628	A	G	0.317	0.045	1.31E-12
SBP	rs573455	11	117267884	A	G	0.199	0.030	4.77E-11
SBP	rs11222084	11	130273230	A	T	-0.336	0.032	1.80E-26
SBP	rs7944927	11	130490917	T	C	0.224	0.039	1.23E-08
SBP	rs78998485	12	434755	C	G	-0.245	0.035	1.48E-12
SBP	rs3819532	12	2436837	T	C	-0.188	0.031	9.44E-10
SBP	rs2024385	12	12888438	A	T	-0.264	0.031	5.88E-18
SBP	rs1010064	12	20000315	A	C	0.357	0.039	3.02E-20
SBP	rs73075659	12	20373541	A	G	0.396	0.032	5.52E-35
SBP	rs2129869	12	26457650	A	T	-0.264	0.036	2.44E-13
SBP	rs9651825	12	27159784	A	G	-0.204	0.034	1.93E-09
SBP	rs61917655	12	48210787	T	C	0.343	0.051	2.68E-11
SBP	rs12426261	12	50573037	A	G	0.378	0.031	2.31E-34
SBP	rs7134440	12	53450097	T	C	0.479	0.056	1.58E-17
SBP	rs7134677	12	54441498	T	C	-0.385	0.033	4.46E-31
SBP	rs7306710	12	66376091	T	C	-0.243	0.030	1.03E-15
SBP	rs4143175	12	67782397	T	C	0.219	0.035	5.10E-10
SBP	rs7963801	12	79685226	T	C	-0.236	0.031	2.87E-14
SBP	rs6539467	12	79955306	A	G	0.265	0.040	5.57E-11
SBP	rs17249754	12	90060586	A	G	-0.845	0.040	1.25E-97
SBP	rs10777213	12	90349999	A	G	-0.179	0.030	2.45E-09
SBP	rs5742643	12	102837863	T	C	-0.223	0.035	1.53E-10

SBP	rs7310615	12	111865049	C	G	0.585	0.031	1.32E-81
SBP	rs1896326	12	115342956	A	G	-0.280	0.037	4.41E-14
SBP	rs35444	12	115552437	A	G	0.437	0.031	3.47E-45
SBP	rs6490019	12	115920472	A	G	-0.290	0.031	6.61E-21
SBP	rs1169078	12	122416254	C	G	-0.197	0.033	1.68E-09
SBP	rs117206641	12	133086888	T	C	0.315	0.050	2.66E-10
SBP	rs483071	13	22294117	T	C	0.271	0.031	5.09E-18
SBP	rs9507885	13	27951090	T	C	-0.321	0.054	3.23E-09
SBP	rs9508495	13	30146201	T	C	-0.356	0.035	6.34E-24
SBP	rs2065498	13	41893105	T	G	-0.293	0.040	3.36E-13
SBP	rs7491248	13	47180671	A	G	0.216	0.036	2.38E-09
SBP	rs9526707	13	51489186	A	G	-0.204	0.032	2.77E-10
SBP	rs75961402	13	56398286	A	G	0.266	0.042	1.95E-10
SBP	rs17245822	13	73131694	A	C	-0.190	0.031	1.15E-09
SBP	rs78474310	13	73826901	A	G	-0.470	0.073	1.51E-10
SBP	rs6562778	13	74223828	A	G	0.178	0.030	4.96E-09
SBP	rs9549627	13	113652369	A	G	0.285	0.050	1.25E-08
SBP	rs7331680	13	115000650	T	G	0.410	0.042	3.35E-22
SBP	rs365990	14	23861811	A	G	0.225	0.031	5.95E-13
SBP	rs8904	14	35871217	A	G	0.306	0.031	1.71E-22
SBP	rs7493678	14	39400917	A	T	-0.189	0.032	2.31E-09
SBP	rs72683923	14	50735947	T	C	0.959	0.110	3.08E-18
SBP	rs35413927	14	53420358	A	G	-0.300	0.033	5.25E-20
SBP	rs57140819	14	68018247	C	G	0.242	0.040	1.30E-09
SBP	rs11847049	14	69259406	C	G	-0.227	0.036	4.44E-10
SBP	rs11159091	14	75074316	A	G	0.198	0.030	6.79E-11
SBP	rs7154723	14	98590629	A	G	0.253	0.031	2.72E-16
SBP	rs17562391	14	100133250	T	C	0.197	0.031	1.35E-10
SBP	rs75016974	14	100197940	T	C	-0.251	0.044	1.05E-08
SBP	rs12885878	14	104007555	A	G	-0.229	0.037	4.32E-10
SBP	rs8030856	15	40314967	C	G	-0.176	0.031	1.21E-08
SBP	rs28866311	15	41442195	T	G	-0.276	0.030	5.45E-20
SBP	rs4775769	15	48939888	T	G	-0.416	0.052	7.76E-16
SBP	rs3098186	15	50810621	T	C	-0.242	0.030	1.41E-15
SBP	rs2652812	15	63406170	T	C	-0.252	0.035	1.03E-12
SBP	rs28429256	15	66931617	A	G	0.215	0.033	3.89E-11
SBP	rs11636952	15	75114322	T	C	0.531	0.033	4.22E-59
SBP	rs2627313	15	81006712	T	C	0.321	0.030	3.55E-26
SBP	rs2046341	15	86040872	A	G	-0.254	0.038	2.74E-11
SBP	rs77032376	15	90010780	T	C	-0.273	0.043	2.35E-10
SBP	rs4932373	15	91429287	A	C	-0.635	0.033	2.49E-83
SBP	rs12906962	15	95312071	T	C	-0.265	0.033	3.28E-16
SBP	rs2589218	15	96785017	T	C	-0.226	0.034	2.54E-11

SBP	rs4606697	15	100087596	A	G	-0.320	0.052	9.71E-10
SBP	rs11641374	16	1347717	A	C	-0.194	0.031	3.26E-10
SBP	rs12596630	16	2065666	T	C	0.428	0.055	5.01E-15
SBP	rs72778133	16	3578718	T	C	-0.242	0.044	4.98E-08
SBP	rs111929315	16	4136871	A	G	0.315	0.049	8.60E-11
SBP	rs12446456	16	4922201	T	C	-0.300	0.030	2.97E-23
SBP	rs77924615	16	20392332	A	G	-0.408	0.039	1.12E-25
SBP	rs7186298	16	21088031	T	C	-0.232	0.030	1.88E-14
SBP	rs8044992	16	24811207	T	C	0.214	0.033	1.07E-10
SBP	rs34941092	16	50550137	A	G	-0.323	0.043	3.23E-14
SBP	rs4784541	16	51704452	T	C	-0.202	0.031	4.93E-11
SBP	rs2060664	16	60652439	T	C	0.216	0.035	4.06E-10
SBP	rs146550789	16	66781040	T	C	-0.482	0.078	5.64E-10
SBP	rs62047964	16	70729954	T	C	0.512	0.069	9.29E-14
SBP	rs1012089	16	74171973	C	G	-0.192	0.030	1.95E-10
SBP	rs4888408	16	75432824	A	G	0.365	0.031	1.42E-32
SBP	rs12926550	16	81510155	A	G	-0.255	0.032	3.43E-15
SBP	rs3950627	16	86436343	A	C	0.185	0.031	1.82E-09
SBP	rs6540119	16	87984477	A	T	0.202	0.032	3.93E-10
SBP	rs908951	16	89697625	T	C	-0.226	0.032	7.14E-13
SBP	rs9303175	17	1372987	T	G	-0.205	0.033	3.65E-10
SBP	rs11653927	17	2012094	T	C	-0.280	0.031	1.17E-19
SBP	rs113086489	17	7171356	T	C	0.325	0.031	3.80E-26
SBP	rs4511593	17	7455536	T	C	-0.288	0.032	1.28E-19
SBP	rs117285318	17	7870642	T	C	0.441	0.059	6.93E-14
SBP	rs4925159	17	18185510	A	G	0.217	0.031	9.66E-13
SBP	rs7218708	17	19926836	A	G	-0.178	0.030	4.38E-09
SBP	rs1551355	17	30032420	T	C	0.210	0.036	3.89E-09
SBP	rs9899540	17	30777924	A	T	0.201	0.032	1.87E-10
SBP	rs7213273	17	43155914	A	G	-0.400	0.032	6.24E-37
SBP	rs17608766	17	45013271	T	C	-0.690	0.043	2.48E-57
SBP	rs3764400	17	46123932	T	C	0.375	0.045	3.69E-17
SBP	rs9897429	17	47518378	A	G	0.265	0.032	1.19E-16
SBP	rs1000423	17	59475642	T	C	0.414	0.035	6.50E-33
SBP	rs56288724	17	60767135	A	G	-0.218	0.031	2.01E-12
SBP	rs62076622	17	61090958	A	G	0.236	0.038	3.79E-10
SBP	rs6504213	17	62381714	T	C	-0.298	0.031	1.25E-21
SBP	rs1436138	17	75316880	A	G	0.312	0.032	4.73E-23
SBP	rs9302885	17	76799898	A	G	0.224	0.030	1.03E-13
SBP	rs11655604	17	79365861	T	C	-0.203	0.033	1.09E-09
SBP	rs34413141	18	777282	A	T	-0.353	0.039	2.47E-19
SBP	rs62082230	18	22676071	A	T	-0.188	0.035	4.69E-08
SBP	rs1154214	18	24546824	T	G	-0.203	0.031	3.27E-11

SBP	rs56407827	18	42179819	T	C	0.360	0.034	2.78E-26
SBP	rs11874246	18	42596789	T	C	0.286	0.033	3.23E-18
SBP	rs7245140	18	43095231	T	C	-0.337	0.039	7.67E-18
SBP	rs1437649	18	48132646	A	G	-0.219	0.036	8.57E-10
SBP	rs665445	18	51842682	A	C	-0.191	0.033	1.15E-08
SBP	rs10048404	18	54578482	T	C	-0.261	0.032	1.91E-16
SBP	rs10460108	18	73034151	A	G	0.214	0.030	1.12E-12
SBP	rs698748	19	1424888	A	G	0.187	0.033	8.90E-09
SBP	rs149339216	19	2144046	T	C	-0.691	0.078	6.93E-19
SBP	rs68096471	19	5175709	A	G	-0.210	0.034	9.26E-10
SBP	rs12985940	19	7262734	T	C	0.464	0.043	1.08E-26
SBP	rs167479	19	11526765	T	G	-0.564	0.033	7.21E-67
SBP	rs1077795	19	17222584	A	G	0.251	0.034	3.33E-13
SBP	rs62112908	19	22213956	A	G	-0.239	0.042	1.25E-08
SBP	rs28572357	19	31867447	A	C	-0.273	0.031	6.34E-19
SBP	rs1433121	19	32591878	T	C	-0.228	0.033	2.66E-12
SBP	rs33836	19	34008600	T	C	0.177	0.030	6.56E-09
SBP	rs10420519	19	45298461	T	G	-0.492	0.089	2.86E-08
SBP	rs7255933	19	45766729	A	G	0.231	0.035	2.44E-11
SBP	rs11672660	19	46180184	T	C	0.221	0.038	6.32E-09
SBP	rs571689	19	49207554	T	C	0.228	0.030	6.77E-14
SBP	rs73046792	19	49605705	A	G	-0.355	0.043	7.23E-17
SBP	rs6054139	20	6327810	A	G	0.209	0.031	8.23E-12
SBP	rs2423514	20	10693337	A	G	0.301	0.030	1.77E-23
SBP	rs6108787	20	10967214	T	G	-0.427	0.030	5.38E-46
SBP	rs6078093	20	11168669	A	G	-0.185	0.030	1.20E-09
SBP	rs8125763	20	17883531	A	C	0.176	0.030	4.84E-09
SBP	rs17812022	20	19007099	T	C	-0.361	0.053	5.65E-12
SBP	rs6058088	20	30139886	T	G	0.283	0.042	1.14E-11
SBP	rs79384779	20	31214944	T	C	0.318	0.043	1.08E-13
SBP	rs6029756	20	40266681	A	G	-0.271	0.033	1.88E-16
SBP	rs6031431	20	42795152	A	G	-0.262	0.030	7.05E-18
SBP	rs2598	20	47241618	A	G	0.168	0.030	2.87E-08
SBP	rs6090907	20	47410231	A	G	-0.385	0.043	1.29E-19
SBP	rs234623	20	57488964	A	G	-0.180	0.030	2.43E-09
SBP	rs6026744	20	57742388	A	T	-0.713	0.046	7.00E-54
SBP	rs28374392	20	61189717	T	C	0.192	0.034	1.21E-08
SBP	rs6062324	20	62446351	A	G	-0.329	0.036	1.18E-19
SBP	rs2776037	21	16317933	T	C	-0.185	0.031	2.15E-09
SBP	rs1882961	21	16556367	T	C	0.244	0.033	6.69E-14
SBP	rs2833834	21	33814378	A	C	0.218	0.034	1.22E-10
SBP	rs12627651	21	44760603	A	G	0.350	0.034	1.02E-24
SBP	rs34487963	21	44838330	A	C	-0.882	0.124	1.35E-12

SBP	rs7278003	21	44966069	T	C	-0.188	0.030	6.63E-10
SBP	rs2238787	22	19976406	A	G	0.255	0.033	1.45E-14
SBP	rs12321	22	29453193	C	G	-0.229	0.030	3.81E-14
SBP	rs113264678	22	30135079	T	C	0.406	0.073	2.26E-08
SBP	rs8142376	22	32001037	T	C	0.168	0.030	2.20E-08
SBP	rs148140538	22	50228044	T	C	-0.325	0.056	7.39E-09
SBP	rs28578714	22	50727921	T	C	0.207	0.033	2.53E-10
Smoking	rs2708630	1	8447404	T	C	-0.010	0.002	9.99E-10
Smoking	rs56169608	1	18439991	A	G	0.009	0.002	1.00E-08
Smoking	rs72664906	1	32170141	C	T	0.014	0.002	1.85E-09
Smoking	rs12742446	1	33877057	T	G	0.012	0.002	2.74E-10
Smoking	rs951740	1	44011737	A	G	0.017	0.002	3.78E-26
Smoking	rs10458563	1	50612250	G	A	0.017	0.002	2.17E-18
Smoking	rs332827	1	61743160	A	G	-0.009	0.002	2.81E-08
Smoking	rs1937443	1	66469643	G	C	0.014	0.002	1.27E-18
Smoking	rs1084445	1	72996521	C	T	-0.012	0.002	1.80E-10
Smoking	rs11210229	1	73860028	G	A	-0.014	0.002	4.94E-18
Smoking	rs3895907	1	75006027	G	A	-0.013	0.002	1.56E-14
Smoking	rs11162976	1	80822282	C	A	0.011	0.002	6.47E-10
Smoking	rs11162019	1	87913176	T	C	-0.010	0.002	3.40E-09
Smoking	rs12133063	1	91214714	A	C	0.012	0.002	1.15E-12
Smoking	rs12036050	1	96917491	C	T	0.012	0.002	7.18E-11
Smoking	rs10745324	1	112708722	G	A	-0.010	0.002	1.97E-08
Smoking	rs12027999	1	154206358	C	T	-0.018	0.002	1.54E-13
Smoking	rs10753630	1	163790451	T	C	0.011	0.002	9.96E-11
Smoking	rs12079063	1	174079585	G	A	-0.009	0.002	3.88E-08
Smoking	rs147052174	1	179783167	T	G	0.034	0.006	1.31E-08
Smoking	rs12760908	1	190888339	T	C	0.011	0.002	3.90E-08
Smoking	rs55921136	1	210359333	C	T	-0.014	0.002	1.90E-12
Smoking	rs76132272	1	227456065	T	C	0.018	0.003	9.43E-09
Smoking	rs3905125	1	236864459	T	C	0.010	0.002	4.28E-11
Smoking	rs4659805	1	237859755	T	G	0.009	0.002	8.36E-09
Smoking	rs10927039	1	243721726	C	T	-0.013	0.002	2.39E-10
Smoking	rs62107261	2	422144	C	T	-0.026	0.004	2.13E-11
Smoking	rs6728726	2	623976	C	T	0.019	0.002	4.27E-19
Smoking	rs61533748	2	22582968	C	T	0.010	0.002	1.50E-10
Smoking	rs62135525	2	44299879	T	C	-0.022	0.004	4.61E-09
Smoking	rs1004787	2	45159091	A	G	0.016	0.002	3.91E-24
Smoking	rs2678903	2	58137930	G	A	0.010	0.002	1.25E-10
Smoking	rs143909875	2	59294197	C	T	0.018	0.003	3.74E-12
Smoking	rs7585579	2	60024857	G	C	0.013	0.002	1.29E-14
Smoking	rs17432775	2	63466463	T	C	-0.011	0.002	2.55E-08
Smoking	rs12714017	2	80999398	C	T	0.010	0.002	6.57E-10

Smoking	rs11695197	2	97711421	A	G	0.014	0.002	6.28E-09
Smoking	rs9679319	2	104134432	G	T	0.018	0.002	4.52E-23
Smoking	rs6705147	2	133196926	T	C	0.010	0.002	5.24E-09
Smoking	rs34367058	2	137513264	T	C	0.014	0.002	1.36E-13
Smoking	rs13030994	2	146143090	A	G	0.021	0.002	2.42E-39
Smoking	rs1994247	2	156023165	T	G	0.012	0.002	1.25E-14
Smoking	rs11693702	2	162802184	A	T	0.014	0.002	9.33E-19
Smoking	rs13009008	2	174043233	G	A	-0.010	0.002	6.92E-09
Smoking	rs4374330	2	182058432	T	C	0.013	0.002	3.73E-12
Smoking	rs62181771	2	201042954	A	G	0.010	0.002	4.47E-10
Smoking	rs13398418	2	203717108	A	T	0.012	0.002	2.09E-11
Smoking	rs2135160	2	213214467	C	T	0.016	0.003	8.23E-09
Smoking	rs2163413	2	226349200	G	A	-0.014	0.002	3.06E-11
Smoking	rs1485272	3	3727589	C	T	-0.009	0.002	4.07E-08
Smoking	rs4479577	3	5723818	T	C	0.009	0.002	2.11E-08
Smoking	rs7629352	3	16848835	G	A	0.010	0.002	7.82E-09
Smoking	rs11716705	3	34728753	G	A	0.012	0.002	1.40E-10
Smoking	rs12632110	3	50224225	G	A	-0.014	0.002	1.18E-16
Smoking	rs11130381	3	53850005	T	C	-0.009	0.002	4.83E-08
Smoking	rs11926232	3	55936417	G	A	0.021	0.004	1.16E-08
Smoking	rs11720703	3	71060640	T	C	0.011	0.002	5.58E-12
Smoking	rs62258903	3	75102433	G	C	-0.016	0.002	7.20E-12
Smoking	rs62254171	3	84624766	A	G	0.031	0.005	3.73E-09
Smoking	rs62250713	3	85513793	G	A	-0.019	0.002	1.63E-30
Smoking	rs66680800	3	85985324	T	G	-0.013	0.002	1.13E-14
Smoking	rs4857114	3	94213083	T	C	0.009	0.002	3.03E-08
Smoking	rs326341	3	107811142	A	G	-0.010	0.002	6.16E-10
Smoking	rs6438208	3	114170272	A	G	-0.010	0.002	7.33E-09
Smoking	rs1499982	3	117820386	T	C	0.022	0.002	3.01E-23
Smoking	rs62266876	3	128976451	G	C	0.015	0.003	3.06E-08
Smoking	rs34638471	3	146367076	A	G	-0.010	0.002	2.49E-08
Smoking	rs114050142	3	150805177	A	T	-0.023	0.003	2.56E-11
Smoking	rs963354	3	157393770	A	C	0.011	0.002	2.35E-10
Smoking	rs12485391	3	181205593	A	C	0.014	0.002	1.89E-08
Smoking	rs12642744	4	28027176	T	G	-0.012	0.002	5.98E-11
Smoking	rs58400863	4	31184484	A	G	-0.011	0.002	5.03E-12
Smoking	rs77307359	4	34839767	C	T	-0.011	0.002	2.07E-08
Smoking	rs7666804	4	57750148	C	T	-0.011	0.002	1.18E-08
Smoking	rs993700	4	67825894	C	T	-0.014	0.002	1.00E-13
Smoking	rs1503211	4	94079508	A	G	0.010	0.002	3.43E-10
Smoking	rs3934797	4	112467612	A	G	-0.015	0.002	1.90E-13
Smoking	rs13145728	4	140927812	C	G	-0.012	0.002	6.87E-14
Smoking	rs10001365	4	147797214	A	G	-0.015	0.002	4.32E-21

Smoking	rs1490683	4	155362876	C	T	-0.010	0.002	2.84E-08
Smoking	rs6852117	4	173076888	G	C	-0.012	0.002	1.97E-13
Smoking	rs13162305	5	12122698	T	A	0.010	0.002	1.15E-08
Smoking	rs6868892	5	22193780	T	C	0.010	0.002	1.92E-08
Smoking	rs1392446	5	30831387	T	C	0.010	0.002	5.60E-10
Smoking	rs71627577	5	43125795	G	A	-0.017	0.002	5.15E-12
Smoking	rs6861333	5	50703933	G	C	-0.011	0.002	3.12E-10
Smoking	rs9763225	5	60257450	G	A	0.014	0.002	5.38E-12
Smoking	rs10062607	5	79290634	A	C	0.010	0.002	2.28E-10
Smoking	rs4571506	5	87756918	T	C	-0.015	0.002	1.64E-21
Smoking	rs11742625	5	91396113	C	T	0.016	0.002	2.19E-10
Smoking	rs72789627	5	106826477	T	C	-0.017	0.002	1.54E-13
Smoking	rs288181	5	107349285	T	C	-0.009	0.002	4.11E-08
Smoking	rs2313500	5	154808532	T	C	0.013	0.002	1.99E-12
Smoking	rs4044321	5	166989513	G	A	-0.016	0.002	3.04E-23
Smoking	rs2173019	5	167614971	A	T	0.013	0.002	3.47E-10
Smoking	rs39784	5	170559066	A	C	0.012	0.002	6.41E-11
Smoking	rs853684	6	28294550	C	T	-0.012	0.002	8.55E-13
Smoking	rs12209775	6	37372370	G	C	-0.010	0.002	1.29E-10
Smoking	rs111861749	6	50889019	A	G	-0.018	0.003	3.16E-12
Smoking	rs160631	6	52895230	G	T	-0.011	0.002	3.56E-09
Smoking	rs16896199	6	65787895	T	A	0.012	0.002	5.61E-09
Smoking	rs146628116	6	67551428	A	T	0.014	0.002	1.43E-14
Smoking	rs62419578	6	84356959	A	T	0.013	0.002	1.08E-08
Smoking	rs9375371	6	98751680	A	G	0.013	0.002	1.41E-13
Smoking	rs11756490	6	100341774	A	T	-0.014	0.002	6.20E-09
Smoking	rs846781	6	101280434	C	T	-0.012	0.002	4.80E-11
Smoking	rs557544	6	109020332	C	T	-0.009	0.002	2.82E-08
Smoking	rs465646	6	111620758	A	G	-0.024	0.002	1.70E-28
Smoking	rs9402093	6	129353671	T	G	0.010	0.002	3.38E-09
Smoking	rs2876586	6	144862998	A	G	0.009	0.002	1.88E-08
Smoking	rs4401691	6	157720414	G	C	-0.010	0.002	9.42E-09
Smoking	rs10698713	6	158882320	A	G	-0.020	0.004	1.84E-08
Smoking	rs79222572	6	165108555	G	T	0.010	0.002	1.53E-08
Smoking	rs6464024	7	1688369	T	C	-0.012	0.002	3.38E-14
Smoking	rs10950410	7	1909086	G	C	-0.011	0.002	1.54E-12
Smoking	rs13246563	7	3438243	G	C	-0.013	0.002	3.99E-14
Smoking	rs2237303	7	21483605	A	G	-0.010	0.002	6.37E-10
Smoking	rs12112638	7	69735251	G	A	-0.012	0.002	8.74E-12
Smoking	rs7788527	7	70579487	C	T	0.010	0.002	1.58E-08
Smoking	rs2072155	7	77762457	C	T	0.013	0.002	1.16E-13
Smoking	rs3814994	7	88427385	T	G	0.010	0.002	7.44E-09
Smoking	rs3801289	7	96638267	C	A	-0.012	0.002	3.64E-12

Smoking	rs7804551	7	99119110	G	A	-0.015	0.002	1.09E-12
Smoking	rs6959670	7	110909169	T	C	0.010	0.002	7.29E-09
Smoking	rs6973168	7	114963829	G	C	-0.009	0.002	2.70E-09
Smoking	rs10233018	7	117523709	G	A	0.014	0.002	1.49E-18
Smoking	rs1899689	7	121964349	T	C	0.009	0.002	1.72E-08
Smoking	rs2402821	7	126297855	A	G	0.010	0.002	3.51E-09
Smoking	rs10279261	7	133589846	A	G	-0.012	0.002	6.87E-14
Smoking	rs7830359	8	10834258	T	C	-0.010	0.002	3.88E-09
Smoking	rs1565735	8	27426077	A	T	-0.023	0.002	7.49E-32
Smoking	rs7829715	8	59803836	C	T	-0.012	0.002	1.30E-13
Smoking	rs4579569	8	64913762	A	G	0.012	0.002	1.23E-14
Smoking	rs6472232	8	66792632	G	T	-0.009	0.002	1.43E-08
Smoking	rs13261725	8	91866297	C	G	0.013	0.002	3.43E-13
Smoking	rs9987376	8	93190014	G	T	-0.014	0.002	1.02E-17
Smoking	rs13255625	8	144269567	G	A	0.012	0.002	4.03E-12
Smoking	rs4543592	9	3014254	C	T	0.012	0.002	2.51E-14
Smoking	rs12685816	9	8290929	A	G	0.015	0.002	1.66E-10
Smoking	rs10121930	9	11175974	A	T	0.010	0.002	8.01E-10
Smoking	rs56348592	9	16749265	G	A	0.013	0.002	1.81E-08
Smoking	rs10119117	9	29740028	T	C	0.009	0.002	1.80E-08
Smoking	rs72733235	9	38275772	C	T	0.013	0.002	1.86E-09
Smoking	rs1246265	9	86761745	C	T	0.012	0.002	1.27E-11
Smoking	rs4837631	9	122061948	T	C	-0.009	0.002	3.54E-09
Smoking	rs13301073	9	128284378	A	G	0.011	0.002	3.13E-11
Smoking	rs11103667	9	137978360	T	C	0.013	0.002	1.47E-10
Smoking	rs1334557	10	8790819	T	C	0.013	0.002	1.51E-12
Smoking	rs7092291	10	10040004	T	C	-0.010	0.002	7.74E-10
Smoking	rs1291865	10	11082192	T	G	0.012	0.002	2.12E-13
Smoking	rs11258417	10	13533053	T	C	-0.010	0.002	3.83E-10
Smoking	rs11012726	10	21797272	C	T	0.014	0.002	4.25E-17
Smoking	rs1776631	10	31422577	C	T	-0.011	0.002	3.92E-08
Smoking	rs1733756	10	56700221	G	A	0.010	0.002	1.23E-10
Smoking	rs13377168	10	63675271	A	T	-0.015	0.002	2.16E-20
Smoking	rs60453921	10	87362579	A	T	0.013	0.002	1.06E-09
Smoking	rs3781295	10	104140602	A	G	-0.012	0.002	1.13E-12
Smoking	rs10786721	10	104654383	A	C	0.018	0.002	7.91E-27
Smoking	rs11192160	10	106453550	C	G	0.014	0.002	8.66E-10
Smoking	rs4751614	10	118696266	T	A	0.012	0.002	6.35E-11
Smoking	rs911781	10	123966989	G	A	-0.009	0.002	4.99E-08
Smoking	rs9423279	10	125680419	G	C	-0.011	0.002	9.64E-11
Smoking	rs10444314	11	7951242	G	T	-0.009	0.002	1.77E-08
Smoking	rs35891966	11	20129311	A	G	-0.019	0.003	6.52E-10
Smoking	rs6265	11	27679916	T	C	-0.020	0.002	1.86E-22

Smoking	rs2939756	11	41436297	A	G	-0.010	0.002	6.87E-11
Smoking	rs72904370	11	46040015	C	G	-0.011	0.002	1.59E-08
Smoking	rs574835	11	64110668	A	G	-0.010	0.002	2.33E-10
Smoking	rs7947391	11	66186882	G	A	0.009	0.002	7.55E-09
Smoking	rs4944844	11	73311705	C	T	-0.013	0.002	2.33E-10
Smoking	rs7929518	11	85980958	G	A	0.012	0.002	9.06E-10
Smoking	rs7127712	11	112874603	T	A	0.027	0.002	2.19E-65
Smoking	rs540860	11	121530888	G	A	0.012	0.002	3.18E-13
Smoking	rs71491832	11	124611997	G	C	-0.018	0.003	3.72E-10
Smoking	rs540356	11	132203816	A	C	0.012	0.002	4.07E-13
Smoking	rs551739	12	40414075	T	A	0.010	0.002	4.42E-09
Smoking	rs2292239	12	56482180	G	T	0.011	0.002	1.03E-10
Smoking	rs7969559	12	69655167	G	A	-0.011	0.002	3.28E-10
Smoking	rs1111578	12	117913256	T	G	-0.013	0.002	8.22E-09
Smoking	rs1971318	12	121389500	T	C	0.016	0.002	7.80E-13
Smoking	rs404263	12	125801308	T	C	-0.011	0.002	1.55E-10
Smoking	rs7986094	13	31029931	C	A	0.010	0.002	5.99E-09
Smoking	rs61959481	13	55834929	A	G	-0.012	0.002	2.06E-10
Smoking	rs9538536	13	60536321	G	T	-0.009	0.002	4.28E-08
Smoking	rs9529052	13	66940097	A	T	-0.012	0.002	1.85E-13
Smoking	rs9541499	13	69261072	G	T	-0.017	0.003	2.16E-08
Smoking	rs2783130	13	80170160	G	A	-0.009	0.002	2.71E-08
Smoking	rs8001839	13	97110073	G	A	0.009	0.002	3.74E-08
Smoking	rs7333559	13	100546450	A	G	-0.014	0.002	3.24E-13
Smoking	rs7984311	13	100741752	A	G	0.009	0.002	1.77E-08
Smoking	rs2022815	14	28356733	A	G	0.010	0.002	1.12E-09
Smoking	rs66480876	14	32448189	G	C	0.009	0.002	1.38E-08
Smoking	rs9323328	14	58653514	G	A	-0.011	0.002	7.25E-12
Smoking	rs17594561	14	79618750	G	A	0.010	0.002	1.27E-09
Smoking	rs1381287	14	98597552	T	C	0.012	0.002	7.51E-15
Smoking	rs11626595	14	104593623	T	C	-0.015	0.002	2.73E-12
Smoking	rs1435679	15	36399245	A	G	0.010	0.002	2.86E-10
Smoking	rs34488670	15	47684936	C	T	0.018	0.002	8.73E-20
Smoking	rs2711607	15	54117442	T	G	0.011	0.002	4.09E-08
Smoking	rs2289791	15	67476952	T	G	-0.013	0.002	2.87E-12
Smoking	rs11632439	15	80987012	G	A	0.009	0.002	1.31E-08
Smoking	rs12441907	15	83922387	A	C	-0.015	0.002	6.09E-14
Smoking	rs1155641	15	97502995	A	G	0.010	0.002	7.72E-10
Smoking	rs6598539	15	99204483	C	T	0.011	0.002	5.76E-12
Smoking	rs763053	16	735921	C	T	-0.015	0.002	6.71E-16
Smoking	rs11865123	16	5840465	A	T	0.011	0.002	4.10E-08
Smoking	rs56225373	16	13755408	G	T	0.016	0.003	3.13E-08
Smoking	rs12923427	16	17575065	T	C	-0.014	0.002	4.96E-12

Smoking	rs3867541	16	18293027	A	G	-0.010	0.002	9.15E-09
Smoking	rs6497840	16	25351633	A	G	0.013	0.002	1.13E-12
Smoking	rs55942317	16	49626772	A	G	0.017	0.003	4.08E-11
Smoking	rs4785187	16	49766772	A	G	0.012	0.002	2.03E-10
Smoking	rs7205551	16	69605968	A	G	0.010	0.002	1.15E-09
Smoking	rs114900182	16	72629056	G	C	-0.021	0.003	1.45E-12
Smoking	rs4888444	16	75690279	G	A	-0.025	0.004	6.84E-10
Smoking	rs1050847	16	87443734	T	C	-0.011	0.002	3.71E-11
Smoking	rs7195043	16	90020861	T	C	-0.009	0.002	2.13E-08
Smoking	rs8067305	17	1975657	A	G	0.011	0.002	4.95E-12
Smoking	rs11078713	17	7795972	G	A	-0.013	0.002	2.77E-15
Smoking	rs7224742	17	30657058	T	C	-0.011	0.002	1.02E-11
Smoking	rs8069451	17	37504933	C	T	0.011	0.002	1.02E-09
Smoking	rs11656151	17	44068492	G	A	-0.013	0.002	5.61E-12
Smoking	rs12600466	17	50306926	T	A	0.012	0.002	1.30E-11
Smoking	rs745570	17	77781725	G	A	-0.010	0.002	8.70E-11
Smoking	rs12945403	17	80004094	C	T	-0.009	0.002	1.79E-08
Smoking	rs888292	18	25210972	T	A	-0.011	0.002	2.03E-09
Smoking	rs3110590	18	27808203	A	C	0.011	0.002	1.22E-09
Smoking	rs16975171	18	39269650	A	C	-0.017	0.003	5.29E-09
Smoking	rs11873164	18	42659922	T	C	-0.016	0.002	1.63E-12
Smoking	rs1025910	18	49871340	C	G	-0.012	0.002	4.83E-13
Smoking	rs1945737	18	53744545	C	T	-0.010	0.002	8.19E-10
Smoking	rs3213876	18	73183978	C	T	0.012	0.002	5.08E-12
Smoking	rs71367545	18	77576337	A	G	0.012	0.002	1.70E-10
Smoking	rs76608582	19	4474725	A	C	-0.027	0.004	2.76E-13
Smoking	rs58547189	19	18462708	A	G	-0.011	0.002	1.73E-08
Smoking	rs172032	19	18633755	C	T	0.009	0.002	1.56E-08
Smoking	rs11673452	19	33928001	C	T	0.015	0.003	3.40E-09
Smoking	rs1126757	19	55879872	T	C	0.010	0.002	4.15E-11
Smoking	rs192477591	20	19659362	T	C	0.012	0.002	1.73E-08
Smoking	rs6141314	20	31093514	A	G	0.013	0.002	3.59E-13
Smoking	rs6088618	20	33409350	A	G	-0.011	0.002	5.89E-11
Smoking	rs56820925	20	54387374	T	C	-0.011	0.002	2.25E-10
Smoking	rs78175438	21	40663255	C	T	0.016	0.002	2.02E-11
Smoking	rs4819027	21	46495224	G	C	-0.010	0.002	1.34E-08
Smoking	rs9613472	22	27972479	G	A	0.009	0.002	4.02E-08
Smoking	rs134529	22	28781758	C	T	-0.012	0.002	8.86E-13
Smoking	rs5751239	22	42592239	T	C	-0.010	0.002	1.02E-10
Smoking	rs9627272	22	46442288	C	G	-0.010	0.002	1.28E-09
Drinking	rs2093186	1	16000106	T	C	-0.012	0.002	1.01E-09
Drinking	rs12044012	1	33816726	A	G	-0.012	0.002	1.34E-09
Drinking	rs6698883	1	34368735	T	C	-0.016	0.003	1.14E-08

Drinking	rs12121630	1	50977279	A	G	-0.014	0.002	1.59E-08
Drinking	rs2310752	1	66392405	A	G	-0.010	0.002	2.57E-08
Drinking	rs10753661	1	165119792	A	G	-0.011	0.002	1.25E-09
Drinking	rs28680958	1	173848808	A	G	-0.014	0.002	1.08E-10
Drinking	rs823099	1	205669322	A	C	0.011	0.002	2.31E-09
Drinking	rs6531148	2	16650233	C	T	-0.012	0.002	7.58E-09
Drinking	rs1260326	2	27730940	C	T	0.025	0.002	3.16E-46
Drinking	rs75199129	2	44298775	T	A	-0.028	0.004	8.23E-12
Drinking	rs1004787	2	45159091	A	G	0.017	0.002	1.13E-22
Drinking	rs68084872	2	58082205	A	G	-0.011	0.002	6.40E-09
Drinking	rs6739804	2	63269604	C	T	-0.014	0.002	2.85E-13
Drinking	rs828867	2	74334462	A	G	0.010	0.002	3.44E-09
Drinking	rs11692435	2	98275354	A	G	0.019	0.003	7.20E-11
Drinking	rs13024996	2	144225215	A	C	-0.014	0.002	5.18E-14
Drinking	rs3768650	2	152990932	G	A	-0.011	0.002	1.67E-08
Drinking	rs7588444	2	178185364	C	T	0.013	0.002	1.76E-09
Drinking	rs60026303	3	81285635	G	A	0.012	0.002	3.58E-08
Drinking	rs28732378	3	85403892	G	A	-0.020	0.002	1.55E-23
Drinking	rs147711594	3	100893355	T	G	-0.030	0.005	2.91E-08
Drinking	rs6787172	3	158187811	G	T	-0.011	0.002	7.49E-10
Drinking	rs9875033	3	184028428	C	T	0.012	0.002	7.90E-10
Drinking	rs12646808	4	3249828	C	T	-0.011	0.002	2.47E-09
Drinking	rs111203819	4	18371143	G	T	0.010	0.002	2.41E-08
Drinking	rs11940694	4	39414993	G	A	0.028	0.002	9.51E-56
Drinking	rs16854020	4	42117559	A	G	0.019	0.003	6.28E-13
Drinking	rs1229984	4	100239319	C	T	0.193	0.005	1.17E-283
Drinking	rs62305763	4	100289405	T	C	0.030	0.003	1.15E-28
Drinking	rs13107325	4	103188709	T	C	-0.039	0.004	2.86E-28
Drinking	rs2079106	4	113282950	G	C	0.010	0.002	3.38E-08
Drinking	rs11943397	4	143617304	C	T	0.012	0.002	1.23E-11
Drinking	rs28694391	4	174154311	C	T	-0.013	0.002	3.81E-09
Drinking	rs4481304	5	50759415	A	G	-0.011	0.002	1.25E-09
Drinking	rs4916723	5	87854395	C	A	-0.011	0.002	2.74E-10
Drinking	rs56353702	5	132248575	G	A	-0.014	0.003	2.16E-08
Drinking	rs6887908	5	144383685	C	A	-0.010	0.002	4.47E-08
Drinking	rs55872084	5	155902003	T	G	0.012	0.002	4.68E-09
Drinking	rs6899302	5	166846529	C	T	-0.010	0.002	1.70E-09
Drinking	rs28361092	6	33443983	A	G	-0.013	0.002	3.42E-09
Drinking	rs1906252	6	98550289	A	C	0.010	0.002	2.05E-08
Drinking	rs6962879	7	14295168	G	C	0.010	0.002	1.89E-08
Drinking	rs11238438	7	51358762	C	G	0.010	0.002	3.73E-08
Drinking	rs13236841	7	69389859	G	A	-0.014	0.002	1.92E-12
Drinking	rs10236149	7	98977515	G	A	-0.016	0.003	4.40E-10

Drinking	rs10276148	7	103733264	A	G	0.012	0.002	1.19E-11
Drinking	rs322773	7	127762119	G	A	0.010	0.002	1.48E-08
Drinking	rs2533126	7	153473086	A	G	0.013	0.002	1.98E-14
Drinking	rs1838420	8	64960855	T	G	0.010	0.002	1.53E-08
Drinking	rs10956823	8	93140909	T	G	0.012	0.002	8.38E-09
Drinking	rs800578	8	116869477	C	T	0.011	0.002	4.51E-08
Drinking	rs28601761	8	126500031	G	C	0.011	0.002	1.57E-10
Drinking	rs55932213	9	108755622	G	A	0.013	0.002	1.61E-10
Drinking	rs36123652	9	109338067	G	A	-0.017	0.003	5.31E-10
Drinking	rs13288470	9	129688559	T	A	-0.016	0.003	4.05E-09
Drinking	rs61873510	10	102626510	T	G	-0.011	0.002	1.17E-08
Drinking	rs6584893	10	110504315	C	A	-0.011	0.002	3.17E-08
Drinking	rs10743083	11	8636350	G	A	-0.015	0.002	1.64E-10
Drinking	rs2049045	11	27694241	C	G	-0.015	0.002	2.24E-11
Drinking	rs11039216	11	47406592	T	C	0.016	0.002	3.76E-19
Drinking	rs4337071	11	113334100	T	C	-0.015	0.002	5.59E-17
Drinking	rs11607622	11	113526665	T	C	0.015	0.003	2.22E-08
Drinking	rs530916	11	121549091	G	A	0.011	0.002	4.22E-10
Drinking	rs56115085	12	6853615	T	C	-0.013	0.002	4.14E-08
Drinking	rs35011311	12	38616581	T	G	-0.011	0.002	8.95E-09
Drinking	rs4761961	12	51799968	C	A	-0.011	0.002	5.19E-10
Drinking	rs3809162	12	54674235	G	A	0.010	0.002	6.84E-09
Drinking	rs61934664	12	81608058	A	G	-0.012	0.002	8.96E-12
Drinking	rs2087975	12	92074004	G	A	-0.011	0.002	2.02E-09
Drinking	rs34484751	12	123652527	C	A	0.031	0.005	7.86E-10
Drinking	rs34704785	13	68117681	T	C	-0.010	0.002	4.52E-09
Drinking	rs8020892	14	58724089	A	G	-0.013	0.002	2.06E-12
Drinking	rs28929474	14	94844947	T	C	-0.049	0.006	3.28E-14
Drinking	rs9745238	15	49552601	G	C	-0.013	0.002	1.38E-09
Drinking	rs7162115	15	52291273	T	G	0.010	0.002	2.65E-08
Drinking	rs35807116	15	74667953	T	C	0.011	0.002	2.65E-09
Drinking	rs28616142	15	86867867	T	C	0.011	0.002	2.99E-10
Drinking	rs72770409	16	24802331	T	C	-0.024	0.004	3.16E-09
Drinking	rs153106	16	28526897	C	T	-0.016	0.002	9.38E-19
Drinking	rs1558902	16	53803574	A	T	-0.012	0.002	6.42E-11
Drinking	rs11075711	16	69266456	T	C	-0.013	0.002	1.31E-08
Drinking	rs79616692	16	72338507	C	G	0.020	0.003	3.54E-12
Drinking	rs11860773	16	73912503	C	T	-0.016	0.002	1.04E-12
Drinking	rs13332432	16	85721809	G	C	0.013	0.002	1.64E-11
Drinking	rs11078696	17	7459299	T	G	0.020	0.003	4.29E-12
Drinking	rs34121753	17	7733833	G	A	0.011	0.002	1.53E-09
Drinking	rs1971157	17	27925343	C	G	0.010	0.002	2.74E-08
Drinking	rs147431626	17	44335635	A	G	-0.029	0.002	7.52E-32

Drinking	rs148390057	18	37695780	T	C	-0.011	0.002	2.91E-08
Drinking	rs4890444	18	40747457	G	C	0.011	0.002	1.89E-09
Drinking	rs1011392	18	53041903	G	A	-0.010	0.002	1.78E-08
Drinking	rs1942964	18	55027212	G	T	-0.011	0.002	3.89E-09
Drinking	rs838145	19	49248730	A	G	-0.016	0.002	5.62E-21
Drinking	rs55987845	20	18656930	T	C	-0.010	0.002	3.41E-08
Drinking	rs2424645	20	24720026	G	A	-0.012	0.002	1.15E-10
Drinking	rs7284839	22	42010902	T	C	0.016	0.003	3.66E-08
Drinking	rs17884691	22	46481623	A	G	-0.011	0.002	2.91E-08
MVPA	rs6427178	1	169095082	A	G	0.023	0.004	1.71E-08
MVPA	rs1160545	2	100832269	T	C	0.025	0.004	1.73E-09
MVPA	rs336620	3	18628793	C	G	0.024	0.004	4.05E-08
MVPA	rs7613360	3	49916710	T	C	-0.025	0.004	2.77E-09
MVPA	rs1691471	3	85011013	T	C	0.038	0.004	1.73E-19
MVPA	rs2668196	3	165502709	A	T	-0.023	0.004	2.09E-08
MVPA	rs4865512	5	50661601	A	G	0.024	0.004	7.68E-09
MVPA	rs4352559	5	60586625	T	C	0.018	0.003	1.65E-08
MVPA	rs370935521	6	26770791	T	C	0.043	0.008	8.25E-09
MVPA	rs13201721	6	141799534	T	C	0.026	0.004	1.83E-10
MVPA	rs12357890	10	99762693	A	G	0.023	0.004	4.77E-08
MVPA	rs1424751	11	57479732	C	G	-0.025	0.004	2.53E-08
MVPA	rs1625595	11	66078129	T	C	-0.021	0.003	1.90E-11
MVPA	rs568546	11	107321156	T	C	0.024	0.004	5.89E-09
MVPA	rs385301	17	19806828	T	C	-0.028	0.005	1.60E-09
MVPA	rs9903845	17	50291181	A	C	-0.020	0.003	6.05E-09

BMI, body mass index; EA, effect allele; MVPA, moderate-to-vigorous intensity physical activity; NEA, non-effect allele; SBP, systolic blood pressure; SE, standard error; SNP, single nucleotide polymorphism.
